# Identifying priority ecosystem services in tidal wetland restoration

**DOI:** 10.3389/fevo.2024.1260447

**Published:** 2024-07-07

**Authors:** Chloe A. Jackson, Connie L. Hernandez, Susan H. Yee, Maliha S. Nash, Heida L. Diefenderfer, Amy B. Borde, Matthew C. Harwell, Theodore H. DeWitt

**Affiliations:** 1 Oak Ridge Institute for Science and Education, Newport, OR, United States; 2 Gulf Ecosystem Measurement and Modeling Division, Office of Research and Development, Center for Environmental Measurement and Modeling, US Environmental Protection Agency, Gulf Breeze, FL, United States; 3 Pacific Coastal Ecology Branch, Pacific Ecological Systems Division, U.S. Environmental Protection Agency, Newport, OR, United States; 4 Coastal Ecosystems Team, Coastal Research Group, Coastal Sciences Division, Energy and Environment Directorate, Pacific Northwest National Laboratory, Sequim, WA, United States

**Keywords:** ecosystem services, tidal wetlands, restoration, document analysis, prioritization

## Abstract

Classification systems can be an important tool for identifying and quantifying the importance of relationships, assessing spatial patterns in a standardized way, and forecasting alternative decision scenarios to characterize the potential benefits (e.g., ecosystem services) from ecosystem restoration that improve human health and well-being. We present a top-down approach that systematically leverages ecosystem services classification systems to identify potential services relevant for ecosystem restoration decisions. We demonstrate this approach using the U.S. Environmental Protection Agency’s National Ecosystem Service Classification System Plus (NESCS Plus) to identify those ecosystem services that are relevant to restoration of tidal wetlands. We selected tidal wetland management documents from federal agencies, state agencies, wetland conservation organizations, and land stewards across three regions of the continental United States (northern Gulf of Mexico, Mid-Atlantic, and Pacific Northwest) to examine regional and organizational differences in identified potential benefits of tidal wetland restoration activities and the potential user groups who may benefit. We used an automated document analysis to quantify the frequencies at which different wetland types were mentioned in the management documents along with their associated beneficiary groups and the ecological end products (EEPs) those beneficiaries care about, as defined by NESCS Plus. Results showed that a top combination across all three regions, all four organizations, and all four tidal wetland types was the EEP naturalness paired with the beneficiary people who care (existence). Overall, the Mid-Atlantic region and the land steward organizations mentioned ecosystem services more than the others, and EEPs were mentioned in combination with tidal wetlands as a high-level, more general category than the other more specific tidal wetland types. Certain regional and organizations differences were statistically significant. Those results may be useful in identifying ecosystem services-related goals for tidal wetland restoration. This approach for identifying and comparing ecosystem service priorities is broadly transferrable to other ecosystems or decision-making contexts.

## 1 Introduction

Multiple reasons for conducting ecosystem restoration have been documented in the literature and in community-specific planning reports for decades, including improving coastal fisheries (Silva et al., 2019), mitigating climate change (Stickler et al., 2009), and supporting sustainable development goals of water conservation and food production (Adams et al., 2016; Gao and Bian, 2019). Although biodiversity, conservation, and ecological integrity are defined as primary goals of *ecological* restoration, they can also produce co-occurring benefits for people, and moreover, *ecosystem* restoration can aim solely at delivery of ecosystem services (Gann et al., 2019), defined as the outputs of nature that contribute to human well-being (Munns et al., 2015). In many cases, restoration plans have broadly stated goals to improve benefits to people interacting with the restoration site; yet those benefits are not explicitly defined or measured in most restoration monitoring programs (reviewed in Jackson et al., 2022). Instead, pre-restoration planning, monitoring, and restoration outcomes typically focus on the condition of ecological or biological structures or functions, as opposed to site uses or benefits to people. As a result, investments in restoration activities may fail to galvanize public support or achieve desired beneficial use outcomes (Benayas et al., 2009; Meli et al., 2014).

An essential first step to inform restoration planning and implementation at local to regional to national scales involves more explicit recognition of which ecosystem services are restoration priorities or likely to be affected by restoration activities. A consideration of ecosystem services can facilitate conversations about what benefits to people may be gained or lost due to changes in the ecological condition (i.e., to vegetation, fauna, soil, water quality, etc.) at an area of interest (DeWitt et al., 2020). Ecosystem services can inform prioritization of the ecosystem management (i.e., development, protection, restoration) of resources by revealing how people use and benefit from natural versus developed lands in those areas. Ecosystem services can also help site managers communicate progress in language relatable to stakeholders and the public. Given that restoration is expensive, those charged with managing and protecting natural resources (e.g., state and federal agencies, conservation organizations, land stewards) have to make difficult spending decisions that should include assessing and monitoring the benefits of the natural resources being protected or restored (Daoust et al., 2014). Therefore, in our view, ecosystem services should be a part of the ongoing discussion of ecosystem protection and restoration.

However, leaving relevant ecosystem services out of the discussion can lead to restoration decisions that neglect commonly shared benefits to people, are disconnected from what matters to gain public support, or undermine community or management goals (Sharpe et al., 2020). The first step of any effort to examine how a geographically specified site’s condition affects the production of ecosystem services, is to identify those services that could be (or are) produced at the site. Since that could be a long list, the second part of that effort is to prioritize which ecosystem services are of greatest interest to people who are managing, restoring, or using the site. In particular, a beneficiary-focused approach to identifying ecosystem services can help ensure direct relevance to people because these approaches explicitly connect biophysical attributes of nature to the people who use or care about them (DeWitt et al., 2020). The National Ecosystem Services Classification System Plus (NESCS Plus; Newcomer-Johnson et al., 2020) codifies this approach by classifying ecosystem services into three components (i.e., a triplet): an environment type (i.e., the classification of where the ecosystem service is produced); a beneficiary or user (i.e., a classification of the role(s) people take while interacting with nature, by which a benefit is obtained); and an ecological end product (EEP) (i.e., the attribute of nature from which the benefit is derived). For example, a wetland (environment type) provides fish (EEP) to recreational anglers (beneficiary).

Tidal wetland areas are inundated or saturated periodically by tidally driven waters at a frequency and duration sufficient to support, and that under normal circumstances do support, a prevalence of vegetation, fauna, soil types, and microbiota typically adapted for these hydrologic conditions (Cowardin et al., 1979). Tidal wetlands provide numerous ecosystem services to people living in coastal communities, including flood protection, and the loss or degradation of these habitats can diminish the production of ecosystem services and the health, economy, and well-being of coastal communities (Barbier et al., 2011; Engle, 2011; Gilby et al., 2020). Tidal wetlands have been subject to centuries of exploitation (e.g., for agriculture and materials extraction), development (e.g., commercial and home construction, roads and other infrastructure), and degradation (e.g., chemical contamination, nutrient runoff, invasion by non-native species), leading to substantial loss globally, nationally, and regionally (Dahl, 1990; Kennish, 2001; Brophy et al., 2019). Furthermore, rising sea levels and intense coastal storms driven by climate change also diminish the size and condition of tidal wetlands (Cherry and Battaglia, 2019) and these impacts are forecast to increase.

Organizations conducting wetland restoration mention the importance of ecosystem services (e.g., Mobile Bay National Estuary Program (MBNEP), 2013, South Slough National Estuarine Research Reserve (NERR), 2017, and Maryland Department of the Environment (MDE), 2018); however, often they do not include an explicit prioritization element (Diefenderfer et al., 2009) or connections to people or human well-being. Regional identities can exert a strong influence on local perceptions and restoration priorities, driven by shared social and recreational customs, shared histories of land use planning and political decisions, or economic and funding priorities (Cook et al., 2012; Borgstrom et al., 2016). Moreover, organizations operate under different mandates, authorities, jurisdictions, time scales, and constraints, but may share overarching goals (Jackson et al., 2022). To identify which ecosystem services are of greatest priority to the tidal wetland restoration community, we utilized a keyword-search approach using tidal wetland management documents to determine which ecosystem services were mentioned most frequently across documents, the organizations that published the documents, and the U.S. regions where the tidal wetlands occurred. We focused on using “gray literature” management documents as we believed these would more likely identify the multitude of ways that a given tidal wetland provide benefits to people using it, as understood by people managing the wetland. We assessed priority among EEPs, beneficiaries, and tidal wetland types by the frequency each was mentioned across documents. Because of the importance of tidal wetlands to coastal communities and the substantial management efforts to protect and restore these wetlands by local, regional, and federal organizations, the results of this analysis may inform restoration, conservation, or climate adaptation planning decisions. The results may be used by tidal wetland site managers to set management goals (i.e., which ecosystem services to restore or protect) and/or for selecting ecosystem services-based metrics for site condition monitoring and assessment. Moreover, the classification system and assessment approach are broadly transferrable and can be used to identify and compare ecosystem services priorities for other ecosystems or decision-making contexts.

This project presents a top-down, literature content-analysis approach to identify beneficiaries or users of tidal wetlands and prioritize ecosystem services of greatest relevance to them, based on extracting information from existing documents (e.g., Yee et al., 2019). Our specific objectives were to: 1) obtain tidal wetland management documents from the gray literature; 2) create a searchable list of ecosystem services keywords based on NESCS Plus (Newcomer-Johnson et al., 2020) and the National Wetland Inventory (Cowardin et al., 1979) as a consistent and objective means for classifying tidal wetland types, beneficiary groups, and EEPs in the documents; 3) quantify the frequency that ecosystem services triplets (i.e., tidal wetland type, beneficiary group, EEP) were mentioned across management documents using an automated document search to identify co-occurring keywords; and 4) compare the number of mentions of ecosystem services across regions, organizations, and tidal wetland types to identify patterns of priority ecosystem services.

## 2 Methods

The document analysis used in this project was based on an approach and automated search described in Yee et al. (2019) that focused on identifying ecosystem services triplets. Each of the triplet components had classes and subclasses that were defined by their own set of synonymous keywords. To be considered a positive hit for an ecosystem services triplet within a document, keyword matches to all three triplet components had to co-occur within a single sentence, also checking the prior 1–2 and following 1–2 sentences if necessary. The analysis produced a list of ecosystem services triplets, which were prioritized by frequency of occurrence across documents within the set of identified tidal wetland management literature. Those ecosystem services mentioned most frequently across documents were assumed to be of greatest general interest to the restoration community (i.e., wetland restoration managers charged with representing beneficiary interests) represented by the documents analyzed, and consequently given priority ecosystem services status.

The general identification and assessment approach follows four steps:

- Step 1. Obtain tidal wetland management documents from the gray literature;
- Step 2. Create a searchable list of ecosystem services keywords to classify document language by tidal wetland types, beneficiary groups, and EEPs based on NESCS Plus (Newcomer-Johnson et al., 2020) and the National Wetland Inventory (Cowardin et al., 1979);
- Step 3. Quantify the frequency that ecosystem services triplets were mentioned across management documents using an automated document search, developed in R (R Core Team, 2022), to identify co-occurring keywords; and
- Step 4. Compare the number of mentions of ecosystem services across regions, organizations, and tidal wetland types to infer priority ecosystem services.

### 2.1 Step 1: Obtain tidal wetland management documents

We sought to evaluate the degree to which ecosystem services provided by different types of tidal wetlands differed among coastal regions of the United States and among different management-organization categories. The focus of this project was on three coastal regions:

- **Pacific Northwest** (Oregon and Washington);
- **Mid-Atlantic** (Virginia Beach, Virginia, to Ocean City, New Jersey); and
- **Northern Gulf of Mexico** (Louisiana to Apalachicola Bay, Florida)

Within these three regions, a gray literature search was conducted to obtain management documents from organizations that have tidal wetland stewardship missions. The literature included conservation plans, restoration and/or monitoring plans, and property and/or habitat management plans. The compilation and analysis were conducted using gray literature because such reports would likely comprehensively discuss the ecosystem services valued by stakeholders or users of the tidal wetland properties. In contrast, scientific journal articles on tidal wetland conservation, restoration, or management typically focus on a subset of possible ecosystem services germane to addressing specific research questions and may reflect the interest of the investigators to a greater degree than those of stakeholders. It should be noted that the restoration documents reviewed typically did not specify whether community input was used when developing each restoration plan, so these documents may primarily reflect the managers’ priorities as representatives of the beneficiaries in their community. The four categories of organizations included:

- **Federal agencies** (i.e., agencies that manage or restore tidal wetlands on federal property);
- **State and local agencies** (i.e., agencies that manage or restore tidal wetlands on State, County, or City/Town/Township property);
- **Land stewards** (i.e., private and non-governmental organizations that own tidal wetland properties and manage them to sustain natural ecological structure and function); and
- **Wetland conservation organizations** (i.e., private or non-governmental organizations that promote or fund wetland conservation, restoration, or management).

A list of organizations involved with tidal wetland management within these regions and categories was created through expert knowledge of the members of the research team and expert knowledge of colleagues of team members (inside and outside their respective affiliations) located in each geographic region (see Supplementary Material Data Sheet 4 for a full list of organizations). Documents had to be publicly available and accessible on organizations’ websites. Searches within the websites involved two avenues: 1) a search using terms such as (“tidal” or “coastal”) and/or “wetland” and/or (“management” or “conservation” or “restoration”); and 2) reviewing the website contents for programs or departments related to environmental work. The full document list was reviewed by all co-authors to identify potential missing documents. From there, one document was chosen for each organization per coastal region in order to equally represent each organization in the analysis. Document choice was based first on maximizing consistency among branches of a given organization (e.g., the Comprehensive Conservation and Management Plan (CCMP) for the National Estuary Programs) and secondly on recency of publication date. A total of 141 documents were used in the literature analysis (Table 1).

### 2.2 Step 2: Create a searchable list of ecosystem services keywords

The list of tidal wetland classes and subclasses was derived from the National Wetland Inventory (Cowardin et al., 1979) (Table 2A). Beneficiary classes and subclasses were derived from NESCS Plus (Table 2B; Newcomer-Johnson et al., 2020). The EEP classes in NESCS Plus are fairly coarse (e.g., Flora, Fauna), so more detailed subclasses were derived from a tool closely related to NESCS Plus, the Final Ecosystem Goods and Services (FEGS) Scoping Tool (Table 2C; Sharpe et al., 2020).

To ensure consistency in management document review, an automated process using an R-script (see Supplementary Material Data Sheet 1 for code) was used to search each document for triplet components. Given that management documents did not always use the same terminology, particularly considering differences between the study regions, a list of synonymous keywords was developed (modified from Yee et al., 2019) for each triplet component class and subclass listed in Tables 2A–C (see Supplementary Material Data Sheet 3 for the full list of keywords). Keywords could be paired with companion words (Table 3) such that both the keyword and the companion word had to be found in the same sentence (i.e., “AND” as a Boolean operator) in order to have a positive hit for that class or subclass. In most cases, keywords were also paired with exclude words, such that co-occurrence of both the keyword and the exclude word in a single sentence would not be considered a positive hit for that class or subclass (i.e., “BUT NOT” as a Boolean operator). Keywords, companion words, and exclude words were developed through an iterative process of examining preliminary search results and determining when unrelated results were found (i.e., false hits) or anticipated matches based on manual reviews of document text were missing. A final manual review of a random selection of document text was compared to automated results as a final check of consistency.

### 2.3 Step 3: Quantify frequency of ecosystem services mentions across documents

Once the documents were gathered and the searchable list of keywords defined, the R-script was used to conduct an automated search for the ecosystem services triplet components in the documents. The R-script read each document and assigned any sentence to a particular class or subclass of tidal wetland, beneficiary, or EEP, if that sentence contained valid keywords representing that class or subclass. The next step was to identify possible triplets for each sentence by generating all possible combinations of tidal wetland, beneficiary, and EEP classes or subclasses assigned to that sentence. If one of the three components of a triplet was not assigned to a sentence, first one and then two prior or following sentences were also checked for the missing component. If a particular component could not be assigned to a class or subclass, it was assigned as unspecified or unknown. All three components were required for a sentence to be assigned an ecosystem services triplet, and a single sentence could be assigned multiple ecosystem services triplets. This process generated a list of all possible ecosystem services triplets derived from each document. From there, co-authors manually reviewed each ecosystem services triplet to generate a master list of valid triplets based on examining examples of document language and verifying whether the specific combination of tidal wetland, beneficiary, and EEP was indeed representative of document intent, and not just an arbitrary combination.

The quality control process for document analysis was an iterative process that checked: (1) for missing concepts that did not get assigned to a triplet component and needed to be added to the keyword list; (2) for false hits that could be minimized with additional paired companion words or exclude words; (3) that the most likely ecosystem services triplets assigned to each sentence were indeed applicable to that sentence; (4) that valid ecosystem services triplets were not being excluded; and (5) that invalid ecosystem services triplets (i.e., arising from non-sensical combinations of ecosystem services triplet elements) would be culled from the master list of ecosystem services triplets. An example of a non-sensical triplet is brackish or salt marsh, hunters, and fiber material quantity; this would be excluded because hunters do not target fiber material, whereas beneficiaries such as subsisters do target fiber material.

First, the code was run on a randomly selected set of 10 documents. The results were manually checked by reviewing at least two randomly selected example paragraphs per document containing the associated keyword. Any non-sensical hits (e.g., “currently” instead of “current” for the water movement EEP) were addressed with changes to the keyword list and the code was re-run on a new set of randomly selected 10 documents as needed until non-sensical hits were no longer returned.

Second, in an independent verification, automated results from the code were compared to results gathered by four people (two team members and two non-team members) familiar with ecosystem services concepts, using a random sample of documents and pages. The readers had access to the main class and subclass list but not the associated keywords. If the reading caught missing or invalid ecosystem services triplets, the keyword list was revised. Any necessary changes to the keyword list were made until any further iterations produced minimal changes to the final ecosystem services triplet counts across documents (i.e., <10% change in counts).

Once the keyword list was finalized, the code was run on the full set of 141 documents. Each combination of ecosystem services triplets was manually checked by reviewing at least two example paragraphs assigned to that triplet, randomly drawn from all documents. Any false hits, typically arising from multi-concept sentences (e.g., “hunting, agriculture, and fishing”) were excluded from the master ecosystem services triplets list.

### 2.4 Step 4: Compare ecosystem services among regions, organizations, and tidal wetland types

The document analysis was used to identify the frequency with which ecosystem services triplets were mentioned in association with different tidal wetland types for each region and organization. Because documents varied widely in length and structure, the analysis used a presence/absence approach to determine whether a particular ecosystem services triplet was mentioned anywhere within each document. The analysis did not assess the number of sentences in which an ecosystem services triplet was mentioned. Importance of each ecosystem services triplet was then quantified as the percent of documents mentioning that particular class or subclass. For purposes of the analysis, if an ecosystem services triplet was mentioned in a document, then it was assumed that the triplet was “important,” regardless of whether it was specifically identified as a component of a management goal.

The number of documents mentioning specific EEP and beneficiary subclasses, and EEP-by-beneficiary class combinations, was analyzed across regions, organizations, and tidal wetland types using the general linear model. For EEPs and beneficiaries, a generalized linear mixed model (GLMM; Proc GLIMMIX, SAS Institute Inc., 2023) analysis of variance (ANOVA) with multiple comparison of means was used to test differences between regions, organizations, and tidal wetland types. For EEP-by-beneficiary combinations, a similar GLMM (Proc GENMOD, SAS Institute Inc., 2023) was used to test the differences between regions, organizations, and tidal wetland types. Proc GENMOD was also used to assess whether higher priority assignments of EEPs or beneficiaries could be an artifact of document length (i.e., regions or organizations with longer documents were more likely to mention ecosystem services concepts than those with more concise documents).

## 3 Results

In total, more than 5,400 valid combinations of ecosystem services triplets were identified among the 141 documents. The search identified an additional 441 valid combinations where the beneficiary was unspecified (e.g., “potential to reach the local community”). The length of the documents ranged between 4 and 1,218 pages. Among organizations, the documents differed in length (p=0.0002) but a length difference did not occur among regions (p=0.553). See Supplementary Material Table 1 and Data Sheet 2 for summary results and data for all of the statistical tests performed. For each sub-section below, we pose a leading question to guide the analysis of the data.

### 3.1 Do EEPs differ among region, organization, or tidal wetland type?

**FIGURE 1:**
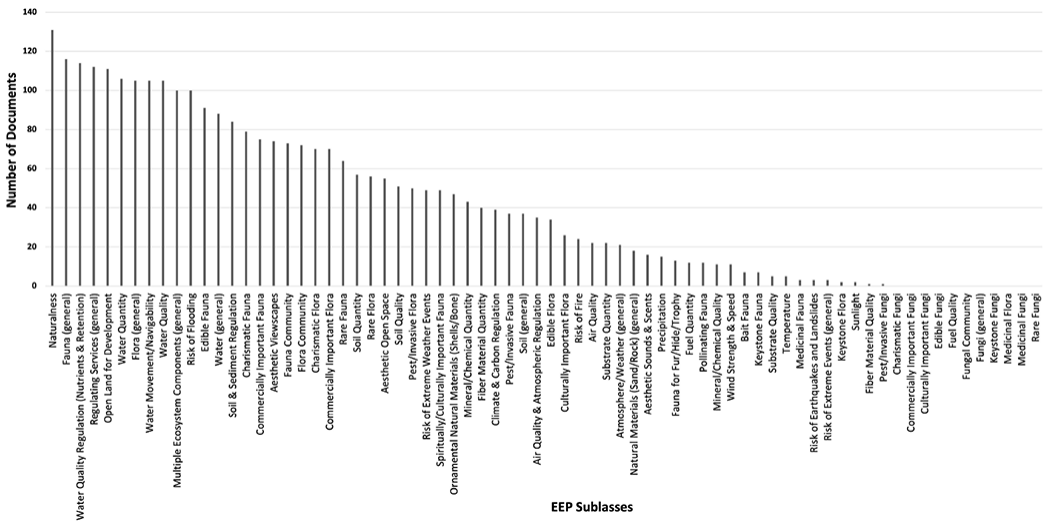
Number of documents mentioning each EEP subclass.

All but 11 of the 71 possible EEP subclasses were mentioned in at least one document (Figure 1); 10 of those not mentioned fell under the fungi class, which indicates that fungi were not a priority in tidal wetland management. The only fungi subclass mentioned was pest/invasive fungi paired with beneficiaries such as agricultural, researchers, and residential property owners. The example paragraphs mentioning pest/invasive fungi referenced mold and fungi killing and/or reducing plant growth. Fuel quality was also not mentioned in any documents; paragraphs containing hits for fuel quantity would mention exploiting forests for fuel and collecting firewood, indicating that perhaps the quantity of fuel is important but not necessarily the quality. The 10 most frequently mentioned EEPs fell under the multiple ecosystem components, fauna, regulating services, water, and flora classes.

Naturalness was the most mentioned EEP followed by fauna (general) in the top 25% of EEPs across regions and organizations (Table 4; refer to the Supplementary Material Table 2 for more detailed Tables 4–9, which show the percentage of documents linking specific EEPs, beneficiaries, and EEP-by-beneficiary combinations for regions, organizations, and tidal wetland types). When looking at regions across all of the documents, the Mid-Atlantic EEPs were statistically different than the Pacific Northwest (p<0.0001) and the Northern Gulf of Mexico (p=0.009). There were no significant differences between the Northern Gulf of Mexico and the Pacific Northwest EEPs (p=0.577). The Mid-Atlantic region tended to focus more on the water related EEPs than the other regions. For example, water quality, water quantity, and water quality regulation (nutrients & retention). The Mid-Atlantic also mentioned flora (general) and multiple ecosystem components (general) more while the Pacific Northwest mentioned water movement/navigability more and the Northern Gulf of Mexico mentioned edible fauna more. Collectively, the Mid-Atlantic region documents mentioned top 25% EEPs more often than the other regions; as stated at the beginning of the results, the regions did not differ significantly in length of documents and each region had a similar number of documents analyzed. Table 10 shows that, although the regions addressed almost the same number of EEPs in at least one document, the Mid-Atlantic region focused on them in more documents than the other regions.

When looking at organizations across all of the documents, land steward and state and local agency EEPs were each significantly different than the other organizations (p<0.0001), but federal agencies and wetland conservation organizations did not significantly differ (p=0.839). Federal agencies mentioned the second highest number of EEPs in at least one document, but they tended to focus on EEPs in the least number of documents. Collectively, land steward documents mentioned top 25% EEPs more than the other organization categories and they also mentioned the highest number of EEPs in at least one document. (The land steward category had more documents with more page numbers to use in the analysis and therefore more opportunities for EEPs to be mentioned).

Across tidal wetland types, naturalness was mentioned the most followed by fauna (general) when comparing the top 25% of EEPs (Table 5). The EEPs of the four tidal wetland classes were statistically different from each other across all of the documents (p<0.0001), with tidal wetlands (general) having the highest mean followed by emergent wetlands, forested wetlands, and then scrub-shrub wetlands. This could indicate that wetland managers focused on different benefits for different tidal wetlands types. Tidal wetlands (general) were mentioned in combination with EEPs in more documents; this could be because most documents focused on tidal wetlands (general) as opposed to a specific tidal wetland type because tidal wetlands (general) were also mentioned with the highest number of EEPs in at least one document. For tidal wetlands (general), regulating services (general) and water quality regulation (nutrients and retention) was frequently mentioned. Scrub-shrub wetlands were mentioned the least in combination with EEPs, both in terms of the total number of EEPs mentioned with scrub-shrub wetlands in at least one document and the number of documents mentioning this combination. This could be because scrub-shrub wetlands are often neglected or viewed as wasteland and only 41 of the 141 documents mentioned scrub-shrub wetlands by any keyword at all, those mentioned the most being fauna (general) and naturalness. Forested wetlands tended to focus on fauna community and water quality less than tidal wetlands (general) and emergent wetlands.

### 3.2 Do beneficiaries differ among region, organization, or tidal wetland type?

**FIGURE 2:**
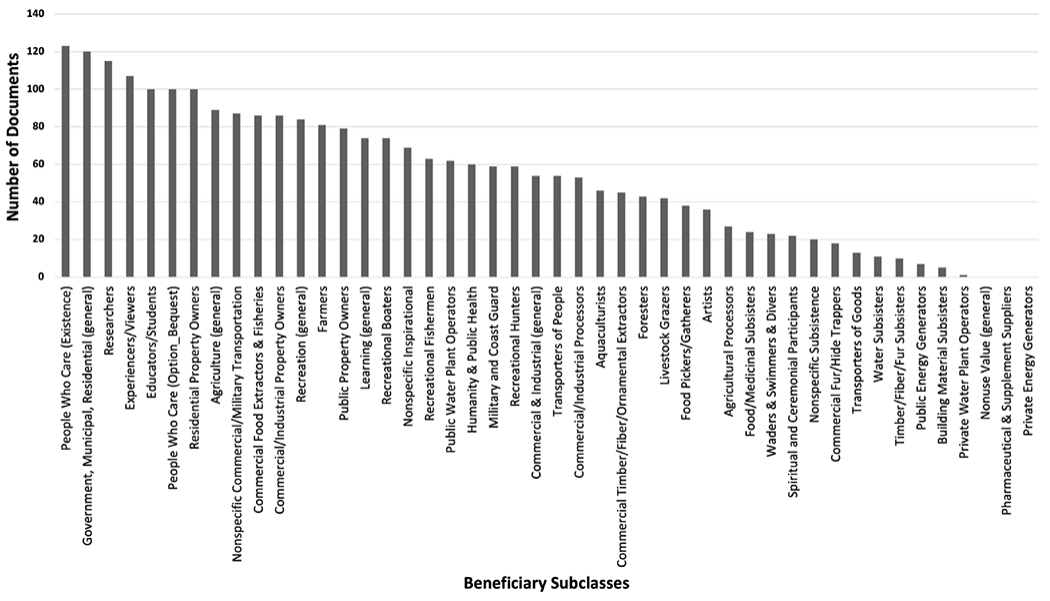
Number of documents mentioning each beneficiary subclass.

All but three beneficiary subclasses were mentioned in at least one document (Figure 2). Those not mentioned were nonuse value (general), pharmaceutical & supplement suppliers (e.g., utilizing tidal wetlands to research, develop, test, etc. medicine, vitamins, drugs, etc.), and private energy generators (or renewable energy sources on private property), which indicates that these were not priority beneficiaries in tidal wetland management. The lack of documents mentioning general nonuse values could be because organizations were more specific when discussing beneficiaries. For example, instead of saying “nonuse resources” a document might say “enhance, protect, and maintain salt marshes” in reference to people who care about salt marshes from an existence standpoint. The 10 most frequently mentioned beneficiaries spanned a wide variety of classes, including nonuse value, government/municipal/residential, learning, recreation, agriculture, and commercial/military transportation.

People who care (existence) was the most mentioned beneficiary followed by government, municipal, residential (general), researchers, and experiencers/viewers in the top 25% of beneficiaries across regions and organizations (Table 6). When looking at regions across all of the documents, the Mid-Atlantic beneficiaries were statistically different than the Pacific Northwest (p<0.0001) and the Northern Gulf of Mexico (p=0.008), as was the case for EEPs. There were no significant differences between the Northern Gulf of Mexico and the Pacific Northwest beneficiaries (p=0.148). Documents from the Northern Gulf of Mexico did not mention agriculture (general) as much as the Mid-Atlantic and the Pacific Northwest regions. Documents from the Northern Gulf of Mexico mentioned commercial food extractors and fisheries more than the Mid-Atlantic and the Pacific Northwest. However, all three regions have very active fishing and seafood harvest industries. Similar to the top 25% of EEPs, the Mid-Atlantic region collectively mentioned top 25% beneficiaries more often than the other regions. Even though the regions addressed almost the same number of beneficiaries in at least one document (Table 11), the Mid-Atlantic region mentioned them in more documents than the other regions.

When looking at organizations across all of the documents, there were similarities to the EEPs because the land steward and state and local agency beneficiaries were each significantly different than the other organizations (p<0.0001), but federal agencies and wetland conservation organizations did not significantly differ (p=0.973). Both federal agencies and wetland conservation organizations mentioned commercial/industrial property owners, residential property owners, and nonspecific commercial/military transportation less than the other organizations indicating that benefits related to property and transportation might be less of a priority for managers in these organizations. Land stewards mentioned commercial food extractors & fisheries, educators/students, and people who care (option/bequest) more than the other organizations. Federal agencies did not mention educators/students as much as the other organizations. This may suggest that local organizations focus on education and outreach programs in their wetland management publications more than other types of organizations. Collectively, land steward documents mentioned beneficiaries in total more often than other organizations and they also mentioned slightly more beneficiaries in at least one document than the other organizations. But, there were more land steward documents and pages used in this analysis, so there was more opportunity for beneficiaries to be mentioned.

Across tidal wetland types, people who care (existence), government, municipal, residential (general), and researchers were mentioned the most when comparing the top 25% of beneficiaries (Table 7). The beneficiaries of the four tidal wetland classes were statistically different from each other across all of the documents (p<0.0001), which lines up with the region and organization results. Tidal wetlands (general) had a higher mean than the others and almost twice as high as emergent wetlands. Recreational boaters were mentioned more for tidal wetlands (general) than the other wetland types and scrub-shrub wetlands was the only wetland type to not mention nonspecific commercial transportation. Similar to the top 25% of EEPs, tidal wetlands (general) were mentioned more with beneficiaries, but this could be because most of the documents focused on tidal wetlands (general) as opposed to a specific tidal wetland type because tidal wetlands (general) were also mentioned with the highest number of beneficiaries in at least one document, although only slightly higher than emergent wetlands. Scrub-shrub wetlands were mentioned the least when it came to beneficiaries, both in terms of number of documents mentioning a scrub-shrub and beneficiary combination, and in terms of the total number of beneficiaries mentioned with scrub-shrubs in at least one document.

### 3.3 Does the use of EEPs by beneficiaries differ among region, organization, or tidal wetland type?

Across all 141 documents, the EEP-by-beneficiary subclass combination most mentioned was naturalness paired with people who care (existence) which showed up in 86% of the documents (Table 8). For example, a document might mention “restoring and protecting rare and endangered species and habitat” because people care about the existence of all species and the prevention of any from going extinct. Naturalness was mentioned with most of the beneficiaries, and people who care benefited from the existence of most of the EEPs. Government, municipal, residential (general) along with researchers were beneficiaries of many of the EEPs and fauna (general) and regulating services (general) were important to most beneficiaries.

When looking at the EEP-by-beneficiary combinations across all documents for regions, all were statistically different from each other (p=0.033). The model did not converge when EEP-by-beneficiary combinations included fungi because the counts were so low, so any combination including fungi was excluded from statistical analyses. The statistical results were interesting because on their own, the Northern Gulf of Mexico and Pacific Northwest EEPs and beneficiaries were not statistically different from each other. Perhaps when making it explicit which beneficiary was being considered for which EEP, differences emerged. For example, in the Pacific Northwest, fauna (general) was a frequently found pair for people who care (existence) (e.g., “restore important habitat for fish”) and more so than the other regions, which focused on naturalness for people who care (option/bequest) more so than the Pacific Northwest (Table 9). An example of the combination naturalness and people who care (option/bequest) would be “protecting the native habitat and its species from future development” which focuses on the option of future generations to benefit from the natural habitat and species. Naturalness paired with people who care (existence) and government, municipal, residential (general) were top combinations across all three regions. An example of the combination naturalness paired with government, municipal, residential (general) would be “restore a key tidal wetland near the center of the city” which addresses natural wetland habitat in the city center without identifying a beneficiary more specific than the city as a whole. All three regions also frequently mentioned naturalness with researchers (e.g., “researchers monitor the ecological outcomes of restoration efforts”) and regulating services (general) with people who care (existence). An example of this combination would be “build resiliency against the effects of climate change and sea level rise” which addresses protecting a coastline against climate change without identifying a beneficiary more specific than people who care about coastal communities.

When looking at the EEP-by-beneficiary combinations across all of the documents for organizations, all were statistically different from each other (p<0.0001). These statistical results were also interesting because on their own, the federal agency and wetland conservation organization EEPs and beneficiaries were not statistically different from each other. Same with regions, this could be because differences emerged when making it explicit which beneficiary was being considered for which EEP. Open land for development, paired with people who care (existence) (e.g., “saving an area of land for potential future development”), was a top combination for land stewards and wetland conservation organizations more so than the other organizations. State and local agencies were the only organization that did not have fauna (general) for people who care (existence) as a top combination and wetland conservation organizations were the only organization to not have regulating services (general) for people who care (existence) as a top combination. As with the regions, naturalness paired with people who care (existence) was a top combination across all four organizations. For state and local agencies and land stewards, naturalness was also a top combination with government, municipal, residential (general). Federal agencies and wetland conservation organizations both frequently mentioned naturalness with researchers, which makes sense as many federal agencies and wetland conservation organizations focus on environmental-based research. For federal agencies, state and local agencies, and land stewards, regulating services (general) was frequently mentioned for people who care (existence). Overall, organizations might have differed in what EEP was important to each beneficiary but had similar top EEPs; so, entities can have similar priority EEPs, but the people using those EEPs may be different.

The EEP-by-beneficiary combinations were statistically different among all tidal wetland types across all of the documents (p<0.0001). These results were similar to the statistical results for EEPs and beneficiaries individually. Naturalness was a top EEP for people who care (existence) for all four tidal wetland types. Naturalness was also frequently mentioned with researchers for all tidal wetland types except scrub-shrub wetlands, showing that naturalness is of interest to those studying certain tidal wetlands. Similarly, scrub-shrub wetlands were the only type to not have naturalness frequently mentioned with the beneficiary government, municipal, residential (general), which could be because, as a whole, governments, municipalities, and residential areas care about the natural tidal wetlands in their communities. People who care (existence) was frequently mentioned with fauna (general) along with regulating services (general); so, in general, these management organizations think that people who care focus on the fauna in tidal wetlands and the ability for the tidal wetland to regulate water, air, etc. A more detailed look at the top 10 combinations of EEP-by-beneficiary subclass combinations across regions, organizations, and tidal wetland types is provided in the Supplementary Material.

## 4 Discussion

When looking at the trends in the priority EEPs across regions, organizations, and tidal wetland types, the documents frequently mentioned many of the same EEPs, indicating shared interests among wetland restoration managers in the different regions and organizations with regard to the tidal wetland types. The Pacific Northwest and the Northern Gulf of Mexico regions were more similar in EEP priorities than the Mid-Atlantic. Federal agencies and wetland conservation organizations each were more similar in EEP priorities than state and local agencies and land stewards. Each of the four tidal wetland types differed from the other three in EEP priorities. Naturalness was the top priority overall with fungi being the least prioritized. Fauna (general) and water quality regulation were also a top priority overall.

Priority beneficiary patterns across regions, organizations, and tidal wetland types showed that documents also frequently mentioned many of the same beneficiaries, indicating shared beneficiary interests among wetland restoration managers. Like priority EEPs, the Pacific Northwest and the Northern Gulf of Mexico documents were more similar in beneficiary priorities than documents from the Mid-Atlantic. Regarding beneficiaries, as with EEPs, federal agencies and wetland conservation organizations each were more similar in beneficiary priorities than state and local agencies and land stewards. Each of the four tidal wetland types differed from the other three in beneficiary priorities. People who care (existence) were the top priority overall and nonuse value (general), pharmaceutical and supplement suppliers, and private energy generators were the lowest priority.

Significant differences among regions suggests that priorities for EEPs and beneficiaries are not the same everywhere within the US. Likewise, differences among organizations in rankings of EEPs and beneficiaries suggests that there may be institutional differences associated with the entities responsible for managing a given tidal wetland. Thus, restoration practitioners should be mindful of these differences in priorities when either working with other wetland stakeholders to develop a restoration plan, or when using the literature to identify priority ecosystem services, EEPs, or beneficiaries. On the positive side, the considerable overlap in which EEPs or beneficiaries were included in the top 25% for each region or organization might suggest that wetland restoration practitioners could choose to start from the “long list” of EEPs or beneficiaries (i.e., Tables 4–7) when working with stakeholders to develop goals and plans.

It is important to acknowledge that ecosystem services include more than just the easily measurable, valued, or useable goods and services such as water quantity. People benefit from nature by simply knowing it exists, so NESCS Plus uses the ‘people who care’ beneficiary subclasses to capture this benefit. Both naturalness and people who care were strongly linked and frequently prioritized across regions, organizations, and tidal wetland types. These results show that wetland restoration managers believe that people who care are important beneficiaries, and the existence of nature is an important benefit to consider when designing restoration projects.

Livestock grazers were a top beneficiary in Pacific Northwest documents. The Pacific Northwest has much farmland in coastal watersheds (Horst, 2019), and coastal tidal wetlands of the Pacific Northwest have been used for farming, particularly cattle grazing, for over a century. Presently, many coastal communities are restoring wetlands that had been converted to farming. For example, Tillamook, Oregon suffers from flooding in winter exacerbated by diking and farming of former wetlands, and the community is currently restoring wetlands to reduce flooding and improve other ecosystem services (Hernandez et al., 2022). Dairy pastures are likewise being restored to wetlands on the Columbia River estuary for juvenile salmon habitat (Littles et al., 2022). Comparatively, there is less livestock grazing in coastal Northern Gulf of Mexico or the Mid-Atlantic regions (Karl et al., 2009), which could explain why livestock grazers did not show up as a top EEP for those two regions.

One limitation of this document analysis approach was the difference in number of pages among documents. To help alleviate this, a presence/absence approach was used to identify and classify an ecosystem services triplet, that is, if a document mentioned an ecosystem services triplet at all, it was assumed to be important to the organization. Another limitation of this approach was the difference in number of documents among management organization categories. For example, there were 56 documents in the land steward category but only 20 each in the federal agency and wetland conservation organization categories. To help alleviate this, the percentage of ecosystem services triplet counts was based on the number of documents in each category as opposed to the total number of documents. Still, the land steward category mentioned ecosystem services more than the other organizations and given that there was sufficient evidence to say that document lengths were different among organizations, the fact that there were more land steward documents could have been a contributing factor. Among regions, the Mid-Atlantic region mentioned about the same number of total EEPs and beneficiaries in at least one document as the other regions, but they mentioned ecosystem services as a whole in more documents than the other regions. There was not sufficient evidence to say that the regions differed in document length, so tidal wetland managers in the Mid-Atlantic region could just be more focused on ecosystem services than in the Northern Gulf of Mexico or Pacific Northwest.

Recent work has shown ecosystem services to be critical to restoration efforts, but infrequently reported by wetland restoration monitoring programs. Ecosystem services can help inform goal setting, project alternative evaluation (Daoust et al., 2014), monitoring and metrics development (Jackson et al., 2022), and engagement with stakeholders and the public (Hernandez et al., 2022). There are many tools available that can help incorporate ecosystem services into restoration (Jackson et al., 2022), many of them that work well with the approach used for this project (i.e., the FEGS Scoping Tool and NESCS Plus). This approach may also be used to complement other approaches for identifying priority ecosystem services. For example, the results from this project complement a more bottom-up approach using the FEGS Scoping Tool in a Tillamook River Wetlands project (Hernandez et al., 2022). In that project, EPA scientists worked with restoration managers on a wetland restoration project along the Tillamook River to prioritize stakeholders, beneficiaries, and environmental attributes of the project. The results from the Tillamook River Wetlands analysis, plus results from this literature-based analysis, can both be used to inform planning for project goals, metrics, and monitoring of the Tillamook River Wetlands restoration project. As stated in the methods, we recognize that the restoration documents reviewed were inconsistent in specifying whether community input was used when developing each restoration plan. Even when direct beneficiary and/or stakeholder outreach is done and welcomed by managers (Pindilli et al., 2018), it is still time consuming and can be difficult to accomplish. Another approach, therefore, is to ask stakeholders and managers to identify and prioritize ecosystem services (Sharpe et al., 2020). The type of document analysis approach used in this paper is the most feasible approach for digesting a large number of documents to draw big-picture conclusions and find cross-region, -organization, and -habitat comparisons. However, if possible, input from local communities and those impacted by a wetland restoration project would be a useful comparison to see how manager priorities compare to beneficiary priorities.

The type of approach used in this paper is well-suited for many circumstances. For example, it can: 1) provide managers with insight before engaging stakeholders; 2) inform managers which stakeholders need to be included (e.g., people who care) and why; 3) help check the results of a stakeholder-driven prioritization for consistency with other restoration goals within a region or tidal wetland type; and 4) provide an opportunity for stakeholders to have priorities presented in understandable language and be able to react/respond to them. Organizations might use this approach to assess whether their restoration or management goals consider the interests of all relevant beneficiaries, and in particular agencies might use it to identify whether management efforts by other organizations will contribute to regional goals, and to identify potential areas of conflict. For example, Yee et al. (2023) utilized this approach in the Massachusetts Bays National Estuary Partnership (MassBays) planning area and identified additional ecosystem services to be included in MassBays’ restoration targets for coastal habitats. This approach was also used in Rossi et al. (2022) to analyze documents for potentially relevant ecosystem services, beneficiaries, and local priorities.

## 5 Conclusion

This analysis provided relevant and prioritized lists of ecosystem services that can be used to inform restoration goal setting and development of monitoring metrics. Strong, yet unexpected commonalities were identified. For example, the EEP naturalness paired with the beneficiary people who care (existence) was a top combination across all three regions, all four organizations, and all four tidal wetland types, and naturalness was a main topic among researchers. The EEPs most frequently mentioned were naturalness and fauna (general).

Certain EEPs can be a top priority for a region or community, such as water quality regulation, but the way an EEP is used changes depending on the specific beneficiary. For example, if the goal of a restoration project is to improve water quality regulation, the regulations would need to be stricter for water being used by waders, swimmers, and divers compared to regulations for areas where people just enjoy the experience/view of the water. This was showcased with organizations where naturalness was a top EEP that was frequently mentioned with researchers for federal agencies and wetland conservation organizations, with residential property owners for state and local agencies, and with experiencer/viewers for land stewards. Each of these beneficiaries would care about naturalness in a different way so restoration and monitoring aims and methods could look different depending on which beneficiaries were of focus. Pinpointing exactly who will benefit from an EEP will help make metrics and the measurement of success more accurate and relevant. This can be important for ensuring restoration efforts are responsive to a full suite of stakeholders and help ensure that communities with different socio-economic backgrounds or interests are not overlooked in capturing priorities for a restoration project. The list of stakeholders for a given decision context may be lengthy but stakeholders need to be carefully considered; in our tidal wetland example, the beneficiaries most frequently mentioned were people who care (existence), government, municipal, residential (general), researchers, and experiencers/viewers.

Those not mentioned, and therefore likely not a known or recognized priority of wetland restoration managers, included the fungi EEP and the pharmaceutical & supplement suppliers and private energy generators beneficiaries. Overall, the Mid-Atlantic region and the land steward organizations mentioned ecosystem services more than the other regions and organizations, and tidal wetlands (general) were mentioned in combination with EEPs and beneficiaries more than the other more specific tidal wetland types.

These methods are transferable to other types of ecosystems, locations, and environmental management problems where there is a need to link site ecological condition to the production of ecosystem services. The power of this approach is that the ecosystem services were prioritized based on the interests described by organizations within these regions that are charged with stewarding and restoring wetlands and informed by their stakeholders. Such lists can be used in future restoration projects to inform: 1) goal setting by identifying socially relevant restoration goals; 2) metrics identification and development based on these goals; and 3) stakeholder engagement and communication with restoration practitioners.

## Supplementary Material

Supplement1

## Figures and Tables

**TABLE 1:** Number of documents analyzed in the literature analysis per organizational category per region.

Organization Categories	Pacific Northwest	Mid-Atlantic	Northern Gulf of Mexico	Total
Federal Agencies	5	5	10	20
State and Local Agencies	13	18	14	45
Land Stewards	17	23	16	56
Wetland Conservation Organizations	10	4	6	20
**Total**	45	50	46	**141**

**TABLE 2A:** Tidal wetland classes and subclasses.

Tidal Wetland Classes	Tidal Wetland Subclasses
Tidal wetland	Tidal wetland unspecified
Emergent wetland	Emergent wetland unspecified
	Brackish or salt marsh
	Emergent tidal fresh wetland
Forested wetland	Forested tidal wetland
	Mangrove
Scrub-shrub wetland	Scrub-shrub wetland

**TABLE 2B:** Beneficiary classes and subclasses.

Beneficiary Classes	Beneficiary Subclasses
Agricultural	Agriculture (general)
	Livestock Grazers
	Agricultural Processors
	Aquaculturists
	Farmers
	Foresters
Commercial and Industrial	Commercial & Industrial (general)
	Commercial Food Extractors & Fisheries
	Commercial Timber/Fiber/Ornamental Extractors
	Commercial/Industrial Processors
	Pharmaceutical & Supplement Suppliers
	Commercial Fur/Hide Trappers
	Private Energy Generators
	Private Water Plant Operators
	Commercial/Industrial Property Owners
Government, Municipal, Residential	Government, Municipal, Residential (general)
	Public Water Plant Operators
	Residential Property Owners
	Military & Coast Guard
	Public Energy Generators
	Public Property Owners
Commercial/Military Transportation	Nonspecific Commercial/Military Transportation
	Transporters of Goods
	Transporters of People
Subsistence	Nonspecific Subsistence
	Water Subsisters
	Timber/Fiber/Fur Subsisters
	Building Material Subsisters
	Food/Medicinal Subsisters
Recreational	Recreation (general)
	Experiencers/Viewers
	Food Pickers/Gatherers
	Recreational Hunters
	Recreational Fishermen
	Waders & Swimmers & Divers
	Recreational Boaters
Inspirational	Nonspecific Inspirational
	Spiritual & Ceremonial Participants
	Artists
Nonuse Value	Nonuse Value (general)
	People Who Care (Existence)
	People Who Care (Option^1^/Bequest)
Humanity	Humanity & Public Health

1 The option for future generations to use, enjoy, or appreciate the existence of the good or service (Newcomer-Johnson et al. 2020).

**TABLE 2C:** EEP classes and subclasses.

EEP Classes	EEP Subclasses
Flora	Flora (general)
	Flora Community
	Edible Flora
	Medicinal Flora
	Keystone Flora
	Charismatic Flora
	Rare Flora
	Commercially Important Flora
	Culturally Important Flora
	Pest/Invasive Flora
Fungi	Fungi (general)
	Fungal Community
	Edible Fungi
	Medicinal Fungi
	Keystone Fungi
	Charismatic Fungi
	Rare Fungi
	Commercially Important Fungi
	Culturally Important Fungi
	Pest/Invasive Fungi
Fauna	Fauna (general)
	Bait Fauna
	Charismatic Fauna
	Commercially Important Fauna
	Edible Fauna
	Fauna Community
	Fauna for Fur/Hide/Trophy
	Keystone Fauna
	Medicinal Fauna
	Pest/Invasive Fauna
	Pollinating Fauna
	Rare Fauna
	Spiritually/Culturally Important Fauna
Soil	Soil (general)
	Soil Quality
	Soil Quantity
	Substrate Quality
	Substrate Quantity
Water	Water (general)
	Water Quality
	Water Quantity
	Water Movement/Navigability
Atmosphere/Weather	Atmosphere/Weather (general)
	Air Quality
	Wind Strength & Speed
	Precipitation
	Sunlight
	Temperature
Natural Materials	Natural Materials (Sand/Rock) (general)
	Fuel Quality
	Fuel Quantity
	Fiber Material Quality
	Fiber Material Quantity
	Mineral/Chemical Quality
	Mineral/Chemical Quantity
	Ornamental Natural Materials (Shells/Bone)
Multiple Ecosystem Components	Multiple Ecosystem Components (general)
	Aesthetic Sounds & Scents
	Aesthetic Viewscapes
	Naturalness
	Aesthetic Open Space
Regulating Services	Regulating Services (general)
	Climate & Carbon Regulation
	Air Quality & Atmospheric Regulation
	Water Quality Regulation (Nutrients & Retention)
	Soil & Sediment Regulation
Risk of Extreme Events	Risk of Extreme Events (general)
	Risk of Flooding
	Risk of Fire
	Risk of Extreme Weather Events
	Risk of Earthquakes & Landslides

**TABLE 3:** Examples of tidal wetland subclass, beneficiary class, and EEP class keywords, companion words, and exclude words from the search term list.

Class/Subclass	Keywords	Companion Words	Exclude Words
Tidal Wetland Subclass	Keywords	Companion Words	Exclude Words
Tidal Wetlands Unspecified	tidal wetland; tidal marsh; tidal swamp	N/A	nontidal; subtidal; inland; freshwater; lake; river; stream
Forested Tidal Wetland	forested wetland; wooded swamp; hammock; woodland	tidal; coast; coastal; salt; saline; estuary	nontidal; subtidal; inland; freshwater; lake; river; stream; beach; dune
Mangrove	mangrove; mangroves	N/A	nontidal; subtidal; inland; freshwater; lake; river; stream
Emergent Wetland Unspecified	emergent wetland; emergent marsh; marshland	tidal; coast; coastal; salt; saline; estuary; bay; brackish	nontidal; subtidal; inland; freshwater; lake; river; stream
Brackish or Salt Marsh	salt marsh; salt hay; brackish marsh	N/A	N/A
Emergent Tidal Fresh Wetland	tidal fresh wetland; tidal river; tidal freshwater marsh; tidal fen	N/A	nontidal; subtidal; inland; freshwater; lake; river; stream; beach
Scrub-shrub Wetland	scrub shrub; shrub swamp; willow; saltbush; orach	tidal; coast; coastal; salt; saline; estuary; bay; brackish	nontidal; subtidal; inland; freshwater; lake; river; stream
Beneficiary Class	Keywords	Companion Words	Exclude Words
Agricultural	agriculture; fertilizer; pesticide; fungicide; herbicide; insecticide	N/A	agriculture department; non-agricultural
Commercial & Industrial	commercial; industrial; business; commerce	N/A	commercial fish; commercial pilot; commercial vessel; recreational industry; noncommercial
Government and Municipal and Residential	government; municipal; village; county; town; city; public use	N/A	legend; capacity; publicity; publication
Commercial/Military Transportation	ship; vessel; air; rail; pilot; captain; navigation; train; navigability	transport; commercial	relationship; sediment transport; shipping; freight; commodity; container
Subsistence	subsistence; tribal; indigenous people; sustenance; traditional use	resource; use; survive	because; horticulture; agriculture; alternative; non-native
Recreational	recreation; vacation; amenities; visitor; tourist	N/A	research; science; student; teach; visiting wildlife; flower-visiting
Inspirational	inspire; cultural significance; cherish; treasure; wonder; beauty	N/A	treasurer; meadow beauty; spring beauty; beautyberry
Learning	learn; museum; visitor center	N/A	lessons learned
Nonuse	non-use; nonuse; non use	resource; opportunity; value	N/A
Humanity	humanity; everyone; humankind; all ages; all people	N/A	activit
EEP Class	Keywords	Companion Words	Exclude Words
Flora	flora; plant; flower; grass; kelp; seaweed; algae; algal; vegetation	benefit; bequest; children; comfort; goods; conserve	processing plant; aguirre plant; planting; grassroot; grass-root
Fungi	fungus; fungi; mushroom	benefit; bequest; children; comfort; goods; conserve	fungicide; chemical; oak death; mushroomed; agency; pickerel; by-product
Fauna	animal; fauna; wildlife; mammal; bird; reptile; amphibian; fish; insect	benefit; bequest; children; comfort; goods; conserve	toad flax; goosefoot; snowbird; geoduck; domestic animal; production of animal
Soil	sediment; soil; dirt; mud; clay; loam; stones; rocks; peat; substrate	accommodate; activity; aesthetic; allure; appeal; amaze; amenity	infertile; unproductive; disturbed; anaerobic; drainfield; retention; buffer; infiltration; runoff
Water	Water	accommodate; activity; aesthetic; allure; appeal; amaze; amenity	agency; pickerel; by-product; especial; laboratory observ; field
Atmosphere	atmosphere; weather; climate; cloud; summer; fall; winter; spring	allure; amaze; appreciate; beauty; comfort; desired	rural atmosphere; changing climate; welcoming atmosphere; cloud berry
Other Natural Components	natural material; natural object; aquatic material	accommodate; amenity; benefit; buy; collect; commodities	clay brook; sand dollar; stone lab; fossil fuel; sand barrier; sticker
Composite	nature; environment; ecosystem; coastal; landscape; grass; farmland	accommodate; amenity; benefit; buy; collect; commodities	landfill; product; goods; environmentally; orthophotograph; bay scallop; agency
Regulating or Buffering	nitrogen; carbon; air; atmosphere; water; sediment; erosion; aquatic	sink; replenish; sequestration; remove; improve; filter; buffer	pollut; total nitrogen loading; goal; plan; implement; problem; impact; challenge
Extreme Events	buffer; filter; control; protect; retention; attenuate; mitigate	extreme event; natural disaster; natural hazard	control survey

Keywords that did not need companion or exclude words are marked with N/A.

**TABLE 4:** The top 25% of EEPs across regions and organizations.

EEP Class	EEP Subclass	Regions			Organizations			
		Pacific Northwest	Northern Gulf of Mexico	Mid-Atlantic	Federal Agencies	State & Local Agencies	Wetland Conservation Organizations	Land Stewards
Flora	Flora (general)							
	Commercially Important Flora							
Fauna	Fauna (general)							
	Charismatic Fauna							
	Commercially Important Fauna							
	Edible Fauna							
	Fauna Community							
Water	Water (general)							
	Water Quality							
	Water Quantity							
	Water Movement/Navigability							
Multiple Ecosystem Components	Multiple Ecosystem Components (general)							
	Aesthetic Viewscapes							
	Naturalness							
	Open Land for Development							
Regulating Services	Regulating Services (general)							
	Water Quality Regulation (Nutrients & Retention)							
	Soil & Sediment Regulation							
Risk of Extreme Events	Risk of Flooding							

Regions		Organizations	
Percentiles	Color	Percentiles	Color
Less than 25% threshold		Less than 25% threshold	
0–25%		0–25%	
25.1–50%		25.1–50%	
50.1–75%		50.1–75%	
75.1–95%		75.1–95%	
95.1–100%		95.1–100%	

The colors in the cells represent the percentile thresholds for the top 25% of EEPs within regions and organizations independently. For regions, the top 25% was based on the average frequency of each EEP subclass across the three regions. For organizations, the top 25% was based on the average frequency of each EEP subclass across the four organizations. The cells that are unhighlighted represent EEP subclasses that did not meet the top 25% threshold for either the regions or the organizations but did for the other.

**TABLE 5:** The top 25% of EEPs across tidal wetland types.

EEP Class	EEP Subclass	Tidal Wetlands (general)	Emergent Wetlands (marsh)	Forested Wetlands	Scrub-Shrub Wetlands
Flora	Flora (general)				
	Flora Community				
	Charismatic Flora				
Fauna	Fauna (general)				
	Charismatic Fauna				
	Commercially Important Fauna				
	Edible Fauna				
	Fauna Community				
Water	Water (general)				
	Water Quality				
	Water Quantity				
	Water Movement/Navigability				
Multiple Ecosystem Components	Multiple Ecosystem Components (general)				
	Naturalness				
	Open Land for Development*				
Regulating Services	Regulating Services (general)				
	Water Quality Regulation (Nutrients & Retention)				
Risk of Extreme Events	Risk of Flooding				

Percentiles	Color
0%	
0.01–25%	
25.1–50%	
50.1–75%	
75.1–95%	
95.1–100%	

* In general, for wetlands, an ecosystem service of open land for development may not be practical or allowable.

The colors in the cells represent the percentile thresholds for the top 25% of EEPs within tidal wetland types.

**TABLE 6:** The top 25% of beneficiaries across regions and organizations.

Beneficiary Class	Beneficiary Subclass	Regions			Organizations			
		Pacific Northwest	Northern Gulf of Mexico	Mid-Atlantic	Federal Agencies	State & Local Agencies	Wetland Conservation Organizations	Land Stewards
Agricultural	Agriculture (general)							
Commercial & Industrial	Commercial Food Extractors & Fisheries							
	Commercial/Industrial Property Owners							
Government, Municipal, Residential	Government, Municipal, Residential (general)							
	Residential Property Owners							
	Public Property Owners							
Commercial/Military Transportation	Nonspecific Commercial/Military Transportation							
Recreational	Recreation (general)							
	Experiencers/Viewers							
Learning	Educators/Students							
	Researchers							
Nonuse Value	People Who Care (Existence)							
	People Who Care (Option/Bequest)							

Regions		Organizations	
Percentiles	Color	Percentiles	Color
Less than 25% threshold		Less than 25% threshold	
0–25%		0–25%	
25.1–50%		25.1–50%	
50.1–75%		50.1–75%	
75.1–95%		75.1–95%	
95.1–100%		95.1–100%	

The colors in the cells represent the percentile thresholds for the top 25% of beneficiaries within regions and organizations independently. For regions, the top 25% was based on the average frequency of each beneficiary subclass across the three regions. For organizations, the top 25% was based on the average frequency of each beneficiary subclass across the four organizations. The cells that are unhighlighted represent beneficiary subclasses that did not meet the top 25% threshold for either the regions or the organizations but did for the other.

**TABLE 7:** The top 25% of beneficiaries across tidal wetland types.

Beneficiary Class	Beneficiary Subclass	Tidal Wetlands (general)	Emergent Wetlands (marsh)	Forested Wetlands	Scrub-Shrub Wetlands
Agricultural	Agriculture (general)				
Commercial & Industrial	Commercial Food Extractors & Fisheries				
Government, Municipal, Residential	Government, Municipal, Residential (general)				
	Residential Property Owners				
Commercial/Military Transportation	Nonspecific Commercial Transportation				
Recreational	Recreational (general)				
	Experiencers/Viewers				
	Recreational Boaters				
Learning	Educators/Students				
	Researchers				
Nonuse Value	People Who Care (Existence)				
	People Who Care (Option/Bequest)				

Percentiles	Color
0%	
0.01–25%	
25.1–50%	
50.1–75%	
75.1–95%	
95.1–100%	

The colors in the cells represent the percentile thresholds for the top 25% of EEPs within tidal wetland types.

**TABLE 8:** Top 50% of EEP-by-beneficiary subclass combinations for all documents.

EEP Subclass/Beneficiary Subclass	People Who Care (Existence)	Government, Municipal, Residential (general)	Researchers	People Who Care (Option/Bequest)	Experiencers/Viewers	Educators/Students	Residential Property Owners	Recreation (general)	Commercial/Industrial Property Owners	Agriculture (general)	Nonspecific Commercial/Military Transportation	Commercial Food Extractors Fisheries	Learning (general)	Recreational Boaters	Public Property Owners	Farmers	Recreational Fishermen	Nonspecific Inspirational	Transporters of People	Recreational Hunters	Humanity & Public Health	Commercial & Industrial (general)	Military & Coast Guard
Naturalness																							
Fauna (general)																							
Regulating Services (general)																							
Open Land for Development																							
Water Movement/Navigability																							
Risk of Flooding																							
Water Quality Regulation (Nutrients & Retention)																							
Flora (general)																							
Water Quantity																							
Multiple Ecosystem Components (general)																							
Edible Fauna																							
Water Quality																							
Water (general)																							
Charismatic Fauna																							
Commercially Important Fauna																							
Aesthetic Open Space																							
Fauna Community																							
Flora Community																							
Aesthetic Viewscapes																							
Soil & Sediment Regulation																							
Charismatic Flora																							
Commercially Important Flora																							
Rare Fauna																							
Risk of Extreme Weather Events																							
Soil Quality																							
Soil Quantity																							
Spiritually/Culturally Important Fauna																							
Pest/Invasive Flora																							
Pest/Invasive Fauna																							
Mineral/Chemical Quantity																							
Climate & Carbon Regulation																							
Soil (general)																							
Air Quality & Atmospheric Regulation																							
Culturally Important Flora				■																			
Natural Materials (Sand/Rock) (general)																							
Ornamental Natural Materials (Shells/Bone)																							

Percentiles	Color
0%	
0.01–25%	
25.1–50%	
50.1–75%	
75.1–95%	
95.1–100%	

The colors in the cells represent the percentage of documents that the EEP-by-beneficiary combination showed up in. The percentiles are based on all of the documents.

**TABLE 9:** Comparison of top 5 EEP-by-beneficiary subclass combinations across regions, organizations, and tidal wetland types.

Beneficiary Subclass/EEP Subclass	Fauna (general)	Naturalness	Open Land for Development	Regulating Services (general)	Water Movement/Navigability	Fauna (general)	Naturalness	Open Land for Development	Regulating Services (general)	Water Movement/Navigability	Fauna (general)	Naturalness	Open Land for Development	Regulating Services (general)	Water Movement/Navigability
	Mid-Atlantic					Federal Agencies					Tidal Wetlands (general)				
Experiencers/Viewers															
Government, Municipal, Residential (general)															
People Who Care (Existence)															
People Who Care (Option/Bequest)															
Researchers															
	Gulf of Mexico					State and Local Agencies					Emergent Wetlands				
Experiencers/Viewers															
Government, Municipal, Residential (general)															
People Who Care (Existence)															
People Who Care (Option/Bequest)															
Researchers															
	Pacific Northwest					Land Stewards					Forested Wetlands				
Experiencers/Viewers															
Government, Municipal, Residential (general)															
People Who Care (Existence)															
People Who Care (Option/Bequest)															
Researchers															
						Wetland Conservation Orgs					Scrub-Shrub Wetlands				
Experiencers/Viewers															
Government, Municipal, Residential (general)															
People Who Care (Existence)															
People Who Care (Option/Bequest)															
Researchers															

Regions		Organizations		Tidal Wetland Types	
Percentiles	Color	Percentiles	Color	Percentiles	Color
0%		0%		0%	
0.1–25%		0.1–25%		0.1–25%	
25.1–50%		25.1–50%		25.1–50%	
50.1–75%		50.1–75%		50.1–75%	
75.1–95%		75.1–95%		75.1–95%	
95.1–100%		95.1–100%		95.1–100%	

The colors in the cells represent the percentile thresholds for the top 5 combinations (based on the number of documents a particular combination was mentioned in) within regions, organizations, and tidal wetland types independently.

**TABLE 10:** Out of 72 total EEPs, this table provides the number of EEPs mentioned in at least one document for each region, organization, and tidal wetland type.

Categories	Pacific Northwest	Northern Gulf of Mexico	Mid-Atlantic	Federal Agencies	State and Local Agencies	Wetland Conservation Organizations	Land Stewards	Tidal Wetlands (general)	Emergent Wetlands (marsh)	Forested Wetlands	Scrub-Shrub Wetlands
**Number of EEPs**	58	57	58	53	49	48	59	60	57	49	38

**TABLE 11:** Out of 46 total beneficiaries, this table provides the number of beneficiaries mentioned in at least one document for each region, organization, and tidal wetland type.

Categories	Pacific Northwest	Northern Gulf of Mexico	Mid-Atlantic	Federal Agencies	State and Local Agencies	Wetland Conservation Organizations	Land Stewards	Tidal Wetlands (general)	Emergent Wetlands (marsh)	Forested Wetlands	Scrub-Shrub Wetlands
**Number of beneficiaries**	43	42	42	40	40	40	43	43	42	37	22

## Data Availability

The raw data supporting the conclusions of this article will be made available by the authors, without undue reservation.
