# Supplementary material for: Identifying priority ecosystem services in tidal wetland restoration
Source: Front Ecol Evol. Author manuscript; Available in PMC 2025 Jul 7. (PMC11457110; doi:10.3389/fevo.2024.1260447)
Supplement: Supplement1 [file NIHMS2010490-supplement-Supplement1.zip › Data Sheet 1_Identifying priority ecosystem services in tidal wetland restoration.pdf]

```
directory <- "/Users/chloejackson/Documents/Priority_FEGS/R/"
##pdffolder <-
"C:\\Users\\rojof\\Desktop\\Susan\\WorkStuff\\MassBays\\NEP_FEGS\\ReportsToRun\\"
pdffolder <- "/Users/chloejackson/Documents/Priority_FEGS/R/documents/"

###This is the table of keyword synonyms
###First column is FEGS category, 2nd is name of class, 3rd column is subclass, all additional
columns are synonyms
x <- read.table( file = paste( directory,"Synonyms.csv",sep=""),sep=",",header=TRUE,as.is=TRUE)
names(x)
head(x)

###These are all the plans#####
###First convert the pdfs file to a text file, using Save As in Adobe, they need to be in the same
directory as this code & synonyms list####
filenames<-list.files(path = pdffolder, pattern = "txt", all.files = FALSE,full.names = FALSE,
recursive = FALSE)
filenames

####Remove this to run all files
##random_docs<-as.integer(runif(10,1,NROW(filenames)))   ####draw 10 random documents,
can change 10 to more or fewer
##random_docs=18
##filenames<-filenames[random_docs]

###These are the output files#########
####This will overwrite any existing file, so rename if it matters to you
###output_all_synonyms<-
"SearchOutput\\MassBays\\Embayments\\FEGS_ALL_Synonym_Hits.csv"  ###skipping this
because gets too big; matches to individual keyword for each synonym, lots of duplicates
output_lines_doc<-"outputs/FEGS_Doc_Hits.csv"
output_triplets_subclass<-"outputs/Triplets_SubClass.csv"
output_triplets_subclass_extras<-"outputs/Triplets_SubClass_Possible_FEGS.csv"
output_triplets_subclass_NEPs<-"outputs/Triplets_SubClass_NEP_Count.csv"


###write headings for the output###
###write.table(cbind("Planning
Doc","Category","Class","SubClass","Synonym","Near","Exclude","Line Number","Matched
Words","Text from Planning Doc"),file=paste(directory,output_all_synonyms,sep=""), sep = ",",
row.names=FALSE,col.names=FALSE,append=FALSE)
write.table(cbind("Planning Doc","Category","Class","SubClass","Line Number","Text from
Planning Doc"),file=paste(directory,output_lines_doc,sep=""), sep = ",",
row.names=FALSE,col.names=FALSE,append=FALSE)
```

```
for(k in 1:NROW(filenames)){   ##cycle through all the planning docs
  y_by_lines <- readLines(paste(pdffolder,filenames[k],sep=""))
  y_by_lines<-gsub("(\")","",y_by_lines)
  y_by_lines<-gsub("[\f]"," ",y_by_lines)
  y_by_lines<-gsub("[\u008f]",". ",y_by_lines)
  y_by_lines<-gsub("[\u0081]",". ",y_by_lines)
  y_by_lines<-gsub("[•]",". ",y_by_lines)
  y_by_lines<-gsub(" o ",". ",y_by_lines)
  y_by_lines<-gsub("^- ",". ",y_by_lines)
  y_by_lines<-gsub("^[0-9][,]",". ",y_by_lines)
  y_by_lines<-gsub("^[0-9][.]",". ",y_by_lines)
  y_by_lines<-gsub("^[0-9][ ]",". ",y_by_lines)
  y_by_lines<-gsub("^[0-9][0-9][,]",". ",y_by_lines)
  y_by_lines<-gsub("^[0-9][0-9][.]",". ",y_by_lines)
  y_by_lines<-gsub("^[0-9][0-9][ ]",". ",y_by_lines)
  y_by_lines<-gsub("^[0-9][0-9][0-9][,]",". ",y_by_lines)
  y_by_lines<-gsub("^[0-9][0-9][0-9][.]",". ",y_by_lines)
  y_by_lines<-gsub("^[0-9][0-9][0-9][ ]",". ",y_by_lines)
  y_by_lines<-gsub("[+]",". ",y_by_lines)
  y_by_lines<-gsub(" HAB-",". HAB-",y_by_lines)
  y_by_lines<-gsub(" WAQ-",". WAQ-",y_by_lines)
  y_by_lines<-gsub(" EDU-",". EDU-",y_by_lines)

  y_by_lines[y_by_lines==""]<-". "

  y_whole<-paste(y_by_lines,collapse=' ')

  y_whole<-gsub("[.][0-9].acres","x acres",y_whole,ignore.case=TRUE)
  y_whole<-gsub("[.][0-9].hectare","x hectare",y_whole,ignore.case=TRUE)
  y_whole<-gsub("[.][0-9].million","x million",y_whole,ignore.case=TRUE)
  y_whole<-gsub("[.][0-9].billion","x billion",y_whole,ignore.case=TRUE)
  y_whole<-gsub("[.][0-9].trillion","x trillion",y_whole,ignore.case=TRUE)
  y_whole<-gsub("[.][0-9].feet","x feet",y_whole,ignore.case=TRUE)
  y_whole<-gsub("[.][0-9].meters","x meters",y_whole,ignore.case=TRUE)
  y_whole<-gsub("[.][0-9].milli","x milli",y_whole,ignore.case=TRUE)
  y_whole<-gsub("[.][0-9].mg","x mg",y_whole,ignore.case=TRUE)
  y_whole<-gsub("[.][0-9].mile","x mile",y_whole,ignore.case=TRUE)
  y_whole<-gsub("[.][0-9].m[)]","x m)",y_whole,ignore.case=TRUE)
  y_whole<-gsub("[.][0-9].m[,]","x m,",y_whole,ignore.case=TRUE)
  y_whole<-gsub("[.][0-9].m[.]","x m ",y_whole,ignore.case=TRUE)
  y_whole<-gsub("[.][0-9].m[/]","x m/",y_whole,ignore.case=TRUE)
  y_whole<-gsub("[.][0-9]..m[/]","x m/",y_whole,ignore.case=TRUE)
```

```
y_whole<-gsub("[.][0-9].mi[.]","x mi ",y_whole,ignore.case=TRUE)
y_whole<-gsub("[.][0-9].ac[.]","x ac ",y_whole,ignore.case=TRUE)
y_whole<-gsub("[.][0-9].sq[.]","x sq ",y_whole,ignore.case=TRUE)
y_whole<-gsub("[.][0-9].mi[ ]","x mi ",y_whole,ignore.case=TRUE)
y_whole<-gsub("[.][0-9].m[ ]","x m ",y_whole,ignore.case=TRUE)
y_whole<-gsub("[.][0-9][ ]to ","x to ",y_whole,ignore.case=TRUE)
y_whole<-gsub("[.][0-9].[ ]to ","x to ",y_whole,ignore.case=TRUE)
y_whole<-gsub("[.][0-9][ ]and ","x and ",y_whole,ignore.case=TRUE)
y_whole<-gsub("[.][0-9].[ ]and ","x and ",y_whole,ignore.case=TRUE)
y_whole<-gsub("[.][0-9][%]","x percent ",y_whole,ignore.case=TRUE)
y_whole<-gsub("[.][0-9].[%]","x percent ",y_whole,ignore.case=TRUE)
y_whole<-gsub("[.][0-9]..[%]","x percent ",y_whole,ignore.case=TRUE)
y_whole<-gsub("[.][0-9].ac[.]","x ac ",y_whole,ignore.case=TRUE)
y_whole<-gsub("[.][0-9].ft","x ft",y_whole,ignore.case=TRUE)
y_whole<-gsub("[.][0-9].kg","x kg",y_whole,ignore.case=TRUE)
y_whole<-gsub("[.][0-9].km","x km",y_whole,ignore.case=TRUE)
y_whole<-gsub("[.][0-9].cm","x cm",y_whole,ignore.case=TRUE)
y_whole<-gsub("[.][0-9].mm","x mm",y_whole,ignore.case=TRUE)
y_whole<-gsub("[.][0-9].person","x person",y_whole,ignore.case=TRUE)
y_whole<-gsub("[.][0-9].month","x month",y_whole,ignore.case=TRUE)
y_whole<-gsub("[.][0-9].year","x year",y_whole,ignore.case=TRUE)
y_whole<-gsub("[.][0-9].ton","x ton",y_whole,ignore.case=TRUE)
y_whole<-gsub("[.][0-9].square","x square",y_whole,ignore.case=TRUE)
y_whole<-gsub("[.][0-9][\u00b0]C","x C)",y_whole,ignore.case=TRUE)
y_whole<-gsub("[.][0-9].[\u00b0]C","x C)",y_whole,ignore.case=TRUE)
y_whole<-gsub("[.][0-9].[\u00b5]","x micro",y_whole,ignore.case=TRUE)
y_whole<-gsub("[.][0-9]..acres","x acres",y_whole,ignore.case=TRUE)
y_whole<-gsub("[.][0-9]..hectare","x hectare",y_whole,ignore.case=TRUE)
y_whole<-gsub("[.][0-9]..million","x million",y_whole,ignore.case=TRUE)
y_whole<-gsub("[.][0-9]..billion","x billion",y_whole,ignore.case=TRUE)
y_whole<-gsub("[.][0-9]..trillion","x trillion",y_whole,ignore.case=TRUE)
y_whole<-gsub("[.][0-9]..feet","x feet",y_whole,ignore.case=TRUE)
y_whole<-gsub("[.][0-9]..meters","x meters",y_whole,ignore.case=TRUE)
y_whole<-gsub("[.][0-9]..milli","x milli",y_whole,ignore.case=TRUE)
y_whole<-gsub("[.][0-9]..mg","x mg",y_whole,ignore.case=TRUE)
y_whole<-gsub("[.][0-9]..mile","x mile",y_whole,ignore.case=TRUE)
y_whole<-gsub("[.][0-9]..m[)]","x m)",y_whole,ignore.case=TRUE)
y_whole<-gsub("[.][0-9]..m[,]","x m,",y_whole,ignore.case=TRUE)
y_whole<-gsub("[.][0-9]..m[.]","x m ",y_whole,ignore.case=TRUE)
y_whole<-gsub("[.][0-9]..mi[.]","x mi ",y_whole,ignore.case=TRUE)
y_whole<-gsub("[.][0-9]..ac[.]","x ac ",y_whole,ignore.case=TRUE)
y_whole<-gsub("[.][0-9]..sq[.]","x sq ",y_whole,ignore.case=TRUE)
y_whole<-gsub("[.][0-9]..mi[ ]","x mi ",y_whole,ignore.case=TRUE)
y_whole<-gsub("[.][0-9]..m[ ]","x m ",y_whole,ignore.case=TRUE)
```

```
y_whole<-gsub("[.][0-9]..ft","x ft",y_whole,ignore.case=TRUE)
y_whole<-gsub("[.][0-9]..kg","x kg",y_whole,ignore.case=TRUE)
y_whole<-gsub("[.][0-9]..km","x km",y_whole,ignore.case=TRUE)
y_whole<-gsub("[.][0-9]..cm","x cm",y_whole,ignore.case=TRUE)
y_whole<-gsub("[.][0-9]..mm","x mm",y_whole,ignore.case=TRUE)
y_whole<-gsub("[.][0-9]..person","x person",y_whole,ignore.case=TRUE)
y_whole<-gsub("[.][0-9]..month","x month",y_whole,ignore.case=TRUE)
y_whole<-gsub("[.][0-9]..year","x year",y_whole,ignore.case=TRUE)
y_whole<-gsub("[.][0-9]..ton","x ton",y_whole,ignore.case=TRUE)
y_whole<-gsub("[.][0-9]..square","x square",y_whole,ignore.case=TRUE)
y_whole<-gsub("[.][0-9]..[\u00b0]C","x C)",y_whole,ignore.case=TRUE)
y_whole<-gsub("[.][0-9]..[\u00b5]","x micro",y_whole,ignore.case=TRUE)
y_whole<-gsub("e[.]g[.]","eg",y_whole,ignore.case=TRUE)
y_whole<-gsub("i[.]e[.]","ie",y_whole,ignore.case=TRUE)
y_whole<-gsub("[(]ie[.]","(ie",y_whole,ignore.case=TRUE)
y_whole<-gsub("N[.]J[.]A[.]C[.]","NJAC",y_whole,ignore.case=TRUE)
y_whole<-gsub("F[.]A[.]C[.]","FAC",y_whole,ignore.case=TRUE)
y_whole<-gsub("R[.]I[.]","RI",y_whole,ignore.case=TRUE)
y_whole<-gsub("B[.]C[.]","BC",y_whole,ignore.case=TRUE)
y_whole<-gsub("N[.]J[.]","NJ",y_whole,ignore.case=TRUE)
y_whole<-gsub("N[.]C[.]","NC",y_whole,ignore.case=TRUE)
y_whole<-gsub("U[.]S[.]C[.]","USC",y_whole,ignore.case=TRUE)
y_whole<-gsub("S[.]C[.]","SC",y_whole,ignore.case=TRUE)
y_whole<-gsub("F[.]S[.]","FS",y_whole,ignore.case=TRUE)
y_whole<-gsub(" etc[.]"," etc",y_whole,ignore.case=TRUE)
y_whole<-gsub(" est[.]"," est",y_whole,ignore.case=TRUE)
y_whole<-gsub(" st[.]"," st",y_whole,ignore.case=TRUE)
y_whole<-gsub(" sp[.]"," sp",y_whole,ignore.case=TRUE)
y_whole<-gsub(" rd[.]"," rd",y_whole,ignore.case=TRUE)
y_whole<-gsub(" spp[.]"," spp",y_whole,ignore.case=TRUE)
y_whole<-gsub(" et al[.]"," et al",y_whole,ignore.case=TRUE)
y_whole<-gsub(" ac[.]"," ac",y_whole,ignore.case=TRUE)
y_whole<-gsub(" m[.]"," m",y_whole,ignore.case=TRUE)
y_whole<-gsub(" mi[.]"," mi",y_whole,ignore.case=TRUE)
y_whole<-gsub(" sq[.]"," sq",y_whole,ignore.case=TRUE)
y_whole<-gsub(" ft[.]"," ft",y_whole,ignore.case=TRUE)
y_whole<-gsub(" inc[.]"," inc",y_whole,ignore.case=TRUE)
y_whole<-gsub(" Dept[.]"," Dept",y_whole,ignore.case=TRUE)
y_whole<-gsub(" Dr[.]"," Dr",y_whole,ignore.case=TRUE)
y_whole<-gsub("[(]etc[.]","(etc",y_whole,ignore.case=TRUE)
y_whole<-gsub("[(]est[.]","(est",y_whole,ignore.case=TRUE)
y_whole<-gsub("[(]st[.]","(st",y_whole,ignore.case=TRUE)
y_whole<-gsub("[(]St[.]","(st",y_whole,ignore.case=TRUE)
y_whole<-gsub("[(]sp[.]","(sp",y_whole,ignore.case=TRUE)
```

```
  y_whole<-gsub("[(]spp[.]","(spp",y_whole,ignore.case=TRUE)
  y_whole<-gsub("[(]Dept[.]","(Dept",y_whole,ignore.case=TRUE)
  y_whole<-gsub("[(]Dr[.]","(Dr",y_whole,ignore.case=TRUE)
  y_whole<-gsub("[-][ ]","",y_whole,ignore.case=TRUE)

  y<-unlist(strsplit(y_whole,"[.]|[;]|\\s[x]\\s|\\s[X]\\s|[:]\\s|\\s[0-9][)]|[(][0-
9][)]|[\u2022]|[\u00B7]"))
  y<-gsub("  "," ",y)
  y<-gsub("^ ","",y)
  y<-gsub("$"," ",y)   ##add a space at the end of sentence
  y<-unique(y)

  for(j in 1:NROW(x)){    ####cycle over each subclass indicator
    ind_hits<-integer(0)
    if(x[j,4]=="include"){
      for(i in 5:NCOL(x)){   ###cycle over each synonym (skipping first four cols)
        if(!(is.na(x[j,i])==TRUE | x[j,i]=="")){  ## if synonym is not blank or NA then proceed
          hits_split<-NA

          near_word=""; excl_word="";

          hits<-grep(x[j,i],y,ignore.case=TRUE)


if(x[min(j+1,NROW(x)),4]=="near"){if(x[j,3]==x[min(j+1,NROW(x)),3]){if(x[min(j+1,NROW(x)),i]!=
""){
           hits<-intersect(hits,grep(x[j+1,i],y,ignore.case=TRUE))
           ##near_word=x[j+1,i];
          }}}

if(x[min(j+2,NROW(x)),4]=="near"){if(x[j,3]==x[min(j+2,NROW(x)),3]){if(x[min(j+2,NROW(x)),i]!=
""){
           hits<-intersect(hits,grep(x[j+2,i],y,ignore.case=TRUE))
           ##near_word=x[j+2,i];
          }}}


if(x[min(j+1,NROW(x)),4]=="exclude"){if(x[j,3]==x[min(j+1,NROW(x)),3]){if(x[min(j+1,NROW(x)),i
]!=""){
           hits<-setdiff(hits,intersect(hits,grep(x[j+1,i],y,ignore.case=TRUE)))
           ##excl_word=x[j+1,i];
          }}}
```

```
if(x[min(j+2,NROW(x)),4]=="exclude"){if(x[j,3]==x[min(j+2,NROW(x)),3]){if(x[min(j+2,NROW(x)),i
]!=""){
          hits<-setdiff(hits,intersect(hits,grep(x[j+2,i],y,ignore.case=TRUE)))
          ##excl_word=x[j+2,i];
        }}}


        ###find which words were hits
        ###if(NROW(hits)>0){
        ###  hits_split<-rep(0,NROW(hits))
        ###  y_split<-strsplit(y[hits]," ")
        ###  for(w in 1:NROW(hits)){
        ###    if(!(near_word=="")){hits_split[w]<-paste(y_split[[w]][grep(gsub(" ","|",gsub("
$","",gsub("^ ","",gsub(" \\|","|",gsub("\\|
","|",paste(x[j,i],"|",near_word,sep=""))))),y_split[[w]],ignore.case=TRUE)],sep="",collapse=",
")}
        ###    if(near_word==""){hits_split[w]<-paste(y_split[[w]][grep(gsub(" ","|",gsub("
$","",gsub("^ ","",gsub(" \\|","|",gsub("\\|
","|",x[j,i]))))),y_split[[w]],ignore.case=TRUE)],sep="",collapse=", ")}
        ###  }
        ###}

        ### this writes ALL matches for ALL synonynms, comment out using ### if not needed

###write.table(cbind(filenames[k],x[j,1],x[j,2],x[j,3],x[j,i],near_word,excl_word,hits,hits_split,y[h
its]),file=paste(directory,output_all_synonyms,sep=""), sep = ",",
row.names=FALSE,col.names=FALSE,append=TRUE,qmethod="double")

        ###remove duplicates for the indicator i
        if(i==5){ind_hits<-hits;}
        if(i>5){ind_hits<-union(ind_hits,hits);}
      }
    }
    ###this writes the lines associated with an indicator (ignores which synonym they came
from)

write.table(cbind(filenames[k],x[j,1],x[j,2],x[j,3],ind_hits,y[ind_hits]),file=paste(directory,output
_lines_doc,sep=""), sep = ",",
row.names=FALSE,col.names=FALSE,append=TRUE,qmethod="double")
   }
  }
}
```

```
#########for testing only######### sorts to random order
###WordMatches <- read.table( file = paste(
directory,output_lines_doc,sep=""),sep=",",header=TRUE,as.is=TRUE,fill=TRUE)
###neword<-runif(nrow(WordMatches),1,20000)
###write.table(WordMatches[order(neword),],file=paste(directory,output_lines_doc,sep=""),
sep = ",", row.names=FALSE,col.names=FALSE,append=FALSE,qmethod="double")

#############################Combine into triplets######################

WordMatches <- read.table( file = paste(
directory,output_lines_doc,sep=""),sep=",",header=TRUE,as.is=TRUE,fill=TRUE)
names(WordMatches)

filenames<-list.files(path = pdffolder, pattern = "txt", all.files = FALSE,full.names = FALSE,
recursive = FALSE)
filenames

#########remove this to run all documents
##filenames<-filenames[random_docs]##########subset to a random 10;

##bensclass<-unique(x$Class[x$Category=="Beneficiary"])
##ecoclass<-unique(x$Class[x$Category=="Environment"])

for(i in 1:NROW(filenames)){

  doc_wm<-WordMatches[WordMatches$Planning.Doc==filenames[i],]
  uniq_lines<-sort(unique(doc_wm$Line.Number))

  for(j in 1:NROW(uniq_lines)){
    paragraph=0 ###set a flag whether or not paragraph was considered
    doc_wm_line <- doc_wm[doc_wm$Line.Number==uniq_lines[j],]
    doc_wm_line <-doc_wm_line[is.na(doc_wm_line$Line.Number)==FALSE,]
    doc_wm_line

    ###if not match also look at 2 prior, 2 following sentences
    doc_wm_paragraph1 <- doc_wm[doc_wm$Line.Number %in% c(uniq_lines[j]-
1,uniq_lines[j]+1),]; doc_wm_paragraph1 <-
doc_wm_paragraph1[is.na(doc_wm_paragraph1$Line.Number)==FALSE,]
    doc_wm_paragraph2 <- doc_wm[doc_wm$Line.Number %in% c(uniq_lines[j]-
2,uniq_lines[j]+2),]; doc_wm_paragraph2 <-
doc_wm_paragraph2[is.na(doc_wm_paragraph2$Line.Number)==FALSE,]

    bens_subclass<- unique(doc_wm_line$SubClass[doc_wm_line$Category=="Beneficiary"])
    eco_subclass<- unique(doc_wm_line$SubClass[doc_wm_line$Category=="Environment"])
```

```
    fegs_subclass<- unique(doc_wm_line$SubClass[doc_wm_line$Category=="FEGS"])


####if no environment, substitute prior sentences if possible     flag as 1 or 2 if came from -1+1,
-2+2 sentence, in order EBF
    if(NROW(eco_subclass)==0){eco_subclass<-
unique(doc_wm_paragraph1$SubClass[doc_wm_paragraph1$Category=="Environment"]);
if(NROW(eco_subclass)!=0){paragraph=1;}}
    if(NROW(eco_subclass)==0){eco_subclass<-
unique(doc_wm_paragraph2$SubClass[doc_wm_paragraph2$Category=="Environment"]);
if(NROW(eco_subclass)!=0){paragraph=2;}}
    if(NROW(bens_subclass)==0){bens_subclass<-
unique(doc_wm_paragraph1$SubClass[doc_wm_paragraph1$Category=="Beneficiary"]);
if(NROW(bens_subclass)!=0){paragraph=paragraph+10;}}
    if(NROW(bens_subclass)==0){bens_subclass<-
unique(doc_wm_paragraph2$SubClass[doc_wm_paragraph2$Category=="Beneficiary"]);
if(NROW(bens_subclass)!=0){paragraph=paragraph+20;}}
    if(NROW(fegs_subclass)==0){fegs_subclass<-
unique(doc_wm_paragraph1$SubClass[doc_wm_paragraph1$Category=="FEGS"]);
if(NROW(fegs_subclass)!=0){paragraph=paragraph+100;}}
    if(NROW(fegs_subclass)==0){fegs_subclass<-
unique(doc_wm_paragraph2$SubClass[doc_wm_paragraph2$Category=="FEGS"]);
if(NROW(fegs_subclass)!=0){paragraph=paragraph+200;}}



    if(NROW(bens_subclass)==0){bens_subclass="Unknown Beneficiary";}
    if(NROW(fegs_subclass)==0){fegs_subclass="blank";}
    if(NROW(eco_subclass)==0){eco_subclass="Environment";}


    if(paragraph==0){paragraph_text<-doc_wm_line$Text.from.Planning.Doc[1]}
    if(paragraph>0){paragraph_text<-
paste(doc_wm_paragraph1$Text.from.Planning.Doc[doc_wm_paragraph1$Line.Number==(uniq
_lines[j]-1)][1],". ",doc_wm_line$Text.from.Planning.Doc[1],".
",doc_wm_paragraph1$Text.from.Planning.Doc[doc_wm_paragraph1$Line.Number==(uniq_lin
es[j]+1)][1])}
    if(paragraph %in% c(2,12,20,21,22,102,120,200,201,202,210,220)){paragraph_text<-
paste(doc_wm_paragraph2$Text.from.Planning.Doc[doc_wm_paragraph2$Line.Number==(uniq
_lines[j]-2)][1],". ",paragraph_text,".
",doc_wm_paragraph2$Text.from.Planning.Doc[doc_wm_paragraph2$Line.Number==(uniq_lin
es[j]+2)][1])}
```

```
####remove higher order category if more detailed subcategory is available
####remove higher order category if more detailed subcategory is available
fegs_class<-x$Class[x$SubClass %in% fegs_subclass & x$Word_type=="include"]
fegs_subclassNA<-fegs_subclass
if(sum(fegs_class==fegs_subclass)>0){  ###contains atleast one higher order category
  for(kk in 1:NROW(fegs_class)){if(fegs_class[kk]==fegs_subclass[kk]){if(fegs_class[kk] %in% fegs_class[-kk]){fegs_subclassNA[kk]<-"NA";}}}
}
fegs_subclass<-fegs_subclass[fegs_subclassNA!="NA"]
bens_class<-x$Class[x$SubClass %in% bens_subclass & x$Word_type=="include"]
bens_subclassNA<-bens_subclass
if(sum(bens_class==bens_subclass)>0){  ###contains atleast one higher order category
  for(kk in 1:NROW(bens_class)){if(bens_class[kk]==bens_subclass[kk]){if(bens_class[kk] %in% bens_class[-kk]){bens_subclassNA[kk]<-"NA";}}}
}
bens_subclass<-bens_subclass[bens_subclassNA!="NA"]
eco_class<-x$Class[x$SubClass %in% eco_subclass & x$Word_type=="include"]
eco_subclassNA<-eco_subclass
if(sum(eco_class==eco_subclass)>0){  ###contains atleast one higher order category
  for(kk in 1:NROW(eco_class)){if(eco_class[kk]==eco_subclass[kk]){if(eco_class[kk] %in% eco_class[-kk]){eco_subclassNA[kk]<-"NA";}}}
}
eco_subclass<-eco_subclass[eco_subclassNA!="NA"]


######combine fauna taxa with fauna category  ###note depends on Fauna taxa being labelled "Fauna_" in keyword synonym list
taxa<-grep("fauna_",fegs_subclass,ignore.case=TRUE)
if(NROW(taxa)>0){
 fauna<-grep("fauna",fegs_subclass,ignore.case=TRUE)
 combined_fauna<-paste(fegs_subclass[fauna[(fauna %in% taxa)==FALSE]],fegs_subclass[taxa],sep=":")
 combined_fauna<-paste(rep(fegs_subclass[fauna[(fauna %in% taxa)==FALSE]],each=NROW(fegs_subclass[taxa])),fegs_subclass[taxa],sep=":")
 fegs_subclass<-c(fegs_subclass[-c(fauna)],combined_fauna)
}
fegs_subclass<-gsub("^:","",fegs_subclass)  ###for non-combined, get rid of :


B<-rep(bens_subclass,NROW(fegs_subclass))
F<-rep(fegs_subclass,each=NROW(bens_subclass))
BE<-rep(B,NROW(eco_subclass))
FE<-rep(F,NROW(eco_subclass))
```

```
   E<-rep(eco_subclass,each=NROW(B))
   BEF<-cbind(BE,E,FE)

   triplet_sc<-
cbind(BEF,rep(doc_wm_line$Line.Number[1],NROW(BEF)),filenames[i],rep(doc_wm_line$Text.f
rom.Planning.Doc[1],NROW(BEF)),rep(paragraph,NROW(BEF)),rep(paragraph_text,NROW(BEF)))
   if((i==1)&&(j==1)){Triplets_sc<-triplet_sc}
   if((i!=1)||(j!=1)){Triplets_sc<-rbind(Triplets_sc,triplet_sc)}
  }
}


Triplets_sc<-as.data.frame(Triplets_sc)
names(Triplets_sc)<-
c("Beneficiary_SubClass","Ecosystem_SubClass","FEGS_SubClass","Line_Number","Document",
"Sentence","Consider_Paragraph","Paragraph_Text")
Triplets_sc<-Triplets_sc[is.na(Triplets_sc$Line_Number)==FALSE,]

Triplets_sc<-
Triplets_sc[order(paste(Triplets_sc$FEGS_SubClass,Triplets_sc$Line_Number,Triplets_sc$Ecosyst
em_SubClass,Triplets_sc$Beneficiary_SubClass)),]

write.table(Triplets_sc,file=paste(directory,output_triplets_subclass,sep=""), sep = ",",
row.names=FALSE,col.names=TRUE,append=FALSE)


####counts across estuaries###
####Triplets_sc<- read.table( file = paste(
directory,"SearchOutput\\NERR\\Triplets_SubClass.csv",sep=""),sep=",",header=TRUE,as.is=TRU
E,fill=TRUE)

trips_est<-
aggregate(Triplets_sc$Line_Number,by=list(Triplets_sc$Beneficiary_SubClass,Triplets_sc$Ecosys
tem_SubClass,Triplets_sc$FEGS_SubClass,Triplets_sc$Document),FUN=length)
names(trips_est)<-
c("Beneficiary_Class","Ecosystem_Class","FEGS_Class","Document","Line_Number")
triplets_estuary<-
aggregate(trips_est$Line_Number,by=list(trips_est$Beneficiary_Class,trips_est$Ecosystem_Clas
s,trips_est$FEGS_Class),FUN=length)
triplets_estuary_total<-
aggregate(trips_est$Line_Number,by=list(trips_est$Beneficiary_Class,trips_est$Ecosystem_Clas
s,trips_est$FEGS_Class),FUN=sum)
triplets_estuary<-cbind(triplets_estuary,triplets_estuary_total$x)
```

```
names(triplets_estuary)<-
c("Beneficiary_SubClass","Ecosystem_SubClass","FEGS_SubClass","Count","Hits")

triplets_estuary$example<-triplets_estuary$Hits

for(i in 1:nrow(triplets_estuary)){
	triplet_matches<-
Triplets_sc[Triplets_sc$Beneficiary_SubClass==triplets_estuary$Beneficiary_SubClass[i],]
	triplet_matches<-
triplet_matches[triplet_matches$Ecosystem_SubClass==triplets_estuary$Ecosystem_SubClass[i]
,]
	triplet_matches<-
triplet_matches[triplet_matches$FEGS_SubClass==triplets_estuary$FEGS_SubClass[i],]
	triplets_estuary$example[i]<-
paste(triplet_matches$Paragraph_Text[floor(runif(1,1,nrow(triplet_matches)+1))])
}

triplets_estuary<-
triplets_estuary[order(paste(triplets_estuary$FEGS_SubClass,triplets_estuary$Ecosystem_SubCl
ass)),]
write.table(triplets_estuary,file=paste(directory,output_triplets_subclass_NEPs,sep=""), sep =
",", row.names=FALSE,col.names=TRUE,append=FALSE)

####First, generate a table of what the triplets are#####
directory <- "/Users/chloejackson/Documents/Priority_FEGS/R/outputs/"


#########Read in the complete list of counts across all documents for each triplet
AllTriplets_Counts<- read.csv( file = paste(
directory,"Triplets_SubClass_NEP_Count.csv",sep=""),sep=",",header=TRUE,as.is=TRUE,fill=FALS
E)
AllTriplets_Counts<-AllTriplets_Counts[AllTriplets_Counts$FEGS_SubClass!="blank",]

AllTriplets_Counts$Count[is.na(AllTriplets_Counts$Count)]<-0
AllTriplets_Counts$Hits[is.na(AllTriplets_Counts$Hits)]<-0
head(AllTriplets_Counts)

#########Join the total counts to the individual sentences in each document  - the total counts
give a rough measure of 'importance' for that triplet
AllTriplets<- read.csv( file = paste(
directory,"Triplets_SubClass.csv",sep=""),sep=",",header=TRUE,as.is=TRUE,fill=FALSE)

########################IF YOU WANT TO SUBSET ALL TRIPLETS, Do IT
HERE#################################################################
```

```
####IF YOU WANT TO SUBSET ALL TRIPLETS, Do IT HERE
####IF YOU WANT TO SUBSET ALL TRIPLETS, Do IT HERE
AllTriplets<-AllTriplets[(AllTriplets$Consider_Paragraph %in%
AllTriplets$Consider_Paragraph[grep(2,AllTriplets$Consider_Paragraph)])==FALSE,]
####Remove paragraph searches of plus/minus TWO sentences
##AllTriplets<-AllTriplets[AllTriplets$Consider_Paragraph==0,]   ####Keep sentences only

###Join the sentences to the Counts
AllTriplets<-
merge(AllTriplets,AllTriplets_Counts[,c("Beneficiary_SubClass","Ecosystem_SubClass","FEGS_Su
bClass","Count","Hits")],by.x=c("Beneficiary_SubClass","Ecosystem_SubClass","FEGS_SubClass")
,by.y=c("Beneficiary_SubClass","Ecosystem_SubClass","FEGS_SubClass"))

###pull out unique sentences
sentences<-
unique(data.frame("Document"=AllTriplets$Document,"Sentence"=AllTriplets$Sentence))

AllTriplets$keep<-rep(0,NROW(AllTriplets))

head(AllTriplets)

###This next step is NOT Essential - it was just a way to help with the QC of the triplets####
###BUT it still needs to be RUN as a step to generate the Triplets Master file, but you can
choose whether or not to consider the "keep" values as you QC or just ignore them
###A sentence might get multiple hits to different Triplets, some real, some false positives
###For each Sentence, assign a "keep" score based on the most likely triplet(s) for each
###the idea here is to assign a likelihood that a given triplet is 'true' for that sentence (keep>=1)
or not true (keep=0), to help with the QC process
###The most likely triplet is assigned based on the total number of counts for all triplets across
all documents
### for each beneficiary, it is based on the most likely Eco/Fegs combinations   (i.e., for this
beneficiary, is this a valid FEG provided by this ecosystem?)
### for each ecosystem, it is based on the most likely Ben/Fegs combinations  (i.e., for this
ecosystem, is this a valid beneficiary using this FEG?)
### for each FEG, it is based on the most likely Ben/eco combinations (i.e., for this FEG, is this a
valid beneficiary likely to use this ecosystem?)
###if all three are true, gets assigned keep=1, if some are true then keep>1, if none are true
then keep=0
### a keep score of zero does not mean a sentence gets dropped, at this point only assigning a
general likelihood

for(i in 1:NROW(sentences)){

  ##i=as.integer(runif(1,1,NROW(sentences)))
```

```
  cc<-AllTriplets[AllTriplets$Document==sentences$Document[i] &
AllTriplets$Sentence==sentences$Sentence[i],]
  unq<-
max(NROW(unique(cc$FEGS_SubClass)),NROW(unique(cc$Beneficiary_SubClass)),NROW(uniqu
e(cc$Ecosystem_SubClass)))

  if(NROW(cc)>unq){
    for(b in 1:NROW(unique(cc$Beneficiary_SubClass))){
      Ben<-cc[cc$Beneficiary_SubClass==unique(cc$Beneficiary_SubClass)[b],]
      unknownF<-
AllTriplets_Counts[AllTriplets_Counts$Beneficiary_SubClass==unique(cc$Beneficiary_SubClass)[
b] & AllTriplets_Counts$FEGS_SubClass=="FEGS" & AllTriplets_Counts$Ecosystem_SubClass
%in% unique(cc$Ecosystem_SubClass),]
      unknownE<-
AllTriplets_Counts[AllTriplets_Counts$Beneficiary_SubClass==unique(cc$Beneficiary_SubClass)[
b] & AllTriplets_Counts$Ecosystem_SubClass=="Environment" &
AllTriplets_Counts$FEGS_SubClass %in% unique(cc$FEGS_SubClass),]
      BenFE<-
rbind(Ben[,c("Beneficiary_SubClass","Ecosystem_SubClass","FEGS_SubClass","Count")],unknow
nF[,c("Beneficiary_SubClass","Ecosystem_SubClass","FEGS_SubClass","Count")],unknownE[,c("B
eneficiary_SubClass","Ecosystem_SubClass","FEGS_SubClass","Count")])
      best<-BenFE$Count[order(BenFE$Count,decreasing=TRUE)[1]]
      AllTriplets$keep[as.integer(row.names(Ben[Ben$Count>=best,]))]<-1
      AllTriplets$keep[as.integer(row.names(Ben[Ben$Count==best-1,]))]<-
ifelse(AllTriplets$keep[as.integer(row.names(Ben[Ben$Count==best-
1,]))]==1,1,ifelse(AllTriplets$keep[as.integer(row.names(Ben[Ben$Count==best-1,]))]==2,2,3))
      BenFE<-Ben
      best<-BenFE$Count[order(BenFE$Count,decreasing=TRUE)[1]]
      AllTriplets$keep[as.integer(row.names(Ben[Ben$Count>=best,]))]<-
ifelse(AllTriplets$keep[as.integer(row.names(Ben[Ben$Count>=best,]))]==1,1,2)
      AllTriplets$keep[as.integer(row.names(Ben[Ben$Count==best-1,]))]<-
ifelse(AllTriplets$keep[as.integer(row.names(Ben[Ben$Count==best-
1,]))]==1,1,ifelse(AllTriplets$keep[as.integer(row.names(Ben[Ben$Count==best-1,]))]==2,2,3))
      if(unique(cc$Beneficiary_SubClass)[b]!="Unknown Beneficiary"){
        BenFE<-Ben[Ben$Ecosystem_SubClass!="Environment" & Ben$FEGS_SubClass!="FEGS",]
        best<-BenFE$Count[order(BenFE$Count,decreasing=TRUE)[1]]
        AllTriplets$keep[as.integer(row.names(BenFE[BenFE$Count>=best,]))]<-
ifelse(AllTriplets$keep[as.integer(row.names(BenFE[BenFE$Count>=best,]))]==1,1,ifelse(AllTriple
ts$keep[as.integer(row.names(BenFE[BenFE$Count>=best,]))]==2,2,ifelse(AllTriplets$keep[as.int
eger(row.names(BenFE[BenFE$Count>=best,]))]==3,3,4)))
        AllTriplets$keep[as.integer(row.names(BenFE[BenFE$Count>=best-1,]))]<-
ifelse(AllTriplets$keep[as.integer(row.names(BenFE[BenFE$Count>=best-
1,]))]==1,1,ifelse(AllTriplets$keep[as.integer(row.names(BenFE[BenFE$Count>=best-
1,]))]==2,2,ifelse(AllTriplets$keep[as.integer(row.names(BenFE[BenFE$Count>=best-
```

```
1,]))]==3,3,ifelse(AllTriplets$keep[as.integer(row.names(BenFE[BenFE$Count>=best-
1,]))]==4,4,5))))
    }
  }

  for(f in 1:NROW(unique(cc$FEGS_SubClass))){
    feg<-cc[cc$FEGS_SubClass==unique(cc$FEGS_SubClass)[f],]
    unknownB<-
AllTriplets_Counts[AllTriplets_Counts$FEGS_SubClass==unique(cc$FEGS_SubClass)[f] &
AllTriplets_Counts$Beneficiary_SubClass=="Unknown Beneficiary" &
AllTriplets_Counts$Ecosystem_SubClass %in% unique(cc$Ecosystem_SubClass),]
    unknownE<-
AllTriplets_Counts[AllTriplets_Counts$FEGS_SubClass==unique(cc$FEGS_SubClass)[f] &
AllTriplets_Counts$Ecosystem_SubClass=="Environment" &
AllTriplets_Counts$Beneficiary_SubClass %in% unique(cc$Beneficiary_SubClass),]
    fegBE<-
rbind(feg[,c("Beneficiary_SubClass","Ecosystem_SubClass","FEGS_SubClass","Count")],unknown
B[,c("Beneficiary_SubClass","Ecosystem_SubClass","FEGS_SubClass","Count")],unknownE[,c("Be
neficiary_SubClass","Ecosystem_SubClass","FEGS_SubClass","Count")])
    best<-fegBE$Count[order(fegBE$Count,decreasing=TRUE)[1]]
    AllTriplets$keep[as.integer(row.names(feg[feg$Count>=best,]))]<-1
    AllTriplets$keep[as.integer(row.names(feg[feg$Count==best-1,]))]<-
ifelse(AllTriplets$keep[as.integer(row.names(feg[feg$Count==best-
1,]))]==1,1,ifelse(AllTriplets$keep[as.integer(row.names(feg[feg$Count==best-1,]))]==2,2,3))
    fegBE<-feg
    best<-fegBE$Count[order(fegBE$Count,decreasing=TRUE)[1]]
    AllTriplets$keep[as.integer(row.names(feg[feg$Count>=best,]))]<-
ifelse(AllTriplets$keep[as.integer(row.names(feg[feg$Count>=best,]))]==1,1,2)
    AllTriplets$keep[as.integer(row.names(feg[feg$Count==best-1,]))]<-
ifelse(AllTriplets$keep[as.integer(row.names(feg[feg$Count==best-
1,]))]==1,1,ifelse(AllTriplets$keep[as.integer(row.names(feg[feg$Count==best-1,]))]==2,2,3))
    if(unique(cc$FEGS_SubClass)[f]!="FEGS"){
      fegBE<-feg[feg$Beneficiary_SubClass!="Unknown Beneficiary" &
feg$Ecosystem_SubClass!="Environment",]
      best<-fegBE$Count[order(fegBE$Count,decreasing=TRUE)[1]]
      AllTriplets$keep[as.integer(row.names(fegBE[fegBE$Count>=best,]))]<-
ifelse(AllTriplets$keep[as.integer(row.names(fegBE[fegBE$Count>=best,]))]==1,1,ifelse(AllTriplet
s$keep[as.integer(row.names(fegBE[fegBE$Count>=best,]))]==2,2,ifelse(AllTriplets$keep[as.inte
ger(row.names(fegBE[fegBE$Count>=best,]))]==3,3,4)))
      AllTriplets$keep[as.integer(row.names(fegBE[fegBE$Count>=best-1,]))]<-
ifelse(AllTriplets$keep[as.integer(row.names(fegBE[fegBE$Count>=best-
1,]))]==1,1,ifelse(AllTriplets$keep[as.integer(row.names(fegBE[fegBE$Count>=best-
1,]))]==2,2,ifelse(AllTriplets$keep[as.integer(row.names(fegBE[fegBE$Count>=best-
```

```
1,]))]==3,3,ifelse(AllTriplets$keep[as.integer(row.names(fegBE[fegBE$Count>=best-
1,]))]==4,4,5))))
    }
  }

  for(e in 1:NROW(unique(cc$Ecosystem_SubClass))){
    eco<-cc[cc$Ecosystem_SubClass==unique(cc$Ecosystem_SubClass)[e],]
    unknownF<-
AllTriplets_Counts[AllTriplets_Counts$Ecosystem_SubClass==unique(cc$Ecosystem_SubClass)[e]
& AllTriplets_Counts$FEGS_SubClass=="FEGS" & AllTriplets_Counts$Beneficiary_SubClass %in%
unique(cc$Beneficiary_SubClass),]
    unknownB<-
AllTriplets_Counts[AllTriplets_Counts$Ecosystem_SubClass==unique(cc$Ecosystem_SubClass)[e]
& AllTriplets_Counts$Beneficiary_SubClass=="Unknown Beneficiary" &
AllTriplets_Counts$FEGS_SubClass %in% unique(cc$FEGS_SubClass),]
    ecoFB<-
rbind(eco[,c("Beneficiary_SubClass","Ecosystem_SubClass","FEGS_SubClass","Count")],unknow
nF[,c("Beneficiary_SubClass","Ecosystem_SubClass","FEGS_SubClass","Count")],unknownB[,c("B
eneficiary_SubClass","Ecosystem_SubClass","FEGS_SubClass","Count")])
    best<-ecoFB$Count[order(ecoFB$Count,decreasing=TRUE)[1]]
    AllTriplets$keep[as.integer(row.names(eco[eco$Count>=best,]))]<-1
    AllTriplets$keep[as.integer(row.names(eco[eco$Count==best-1,]))]<-
ifelse(AllTriplets$keep[as.integer(row.names(eco[eco$Count==best-
1,]))]==1,1,ifelse(AllTriplets$keep[as.integer(row.names(eco[eco$Count==best-1,]))]==2,2,3))
    ecoFB<-eco
    best<-ecoFB$Count[order(ecoFB$Count,decreasing=TRUE)[1]]
    AllTriplets$keep[as.integer(row.names(eco[eco$Count>=best,]))]<-
ifelse(AllTriplets$keep[as.integer(row.names(eco[eco$Count>=best,]))]==1,1,2)
    AllTriplets$keep[as.integer(row.names(eco[eco$Count==best-1,]))]<-
ifelse(AllTriplets$keep[as.integer(row.names(eco[eco$Count==best-
1,]))]==1,1,ifelse(AllTriplets$keep[as.integer(row.names(eco[eco$Count==best-1,]))]==2,2,3))
    if(unique(cc$Ecosystem_SubClass)[e]!="Environment"){
     ecoFB<-eco[eco$Beneficiary_SubClass!="Unknown Beneficiary" &
eco$FEGS_SubClass!="FEGS",]
     best<-ecoFB$Count[order(ecoFB$Count,decreasing=TRUE)[1]]
     AllTriplets$keep[as.integer(row.names(ecoFB[ecoFB$Count>=best,]))]<-
ifelse(AllTriplets$keep[as.integer(row.names(ecoFB[ecoFB$Count>=best,]))]==1,1,ifelse(AllTriple
ts$keep[as.integer(row.names(ecoFB[ecoFB$Count>=best,]))]==2,2,ifelse(AllTriplets$keep[as.int
eger(row.names(ecoFB[ecoFB$Count>=best,]))]==3,3,4)))
     AllTriplets$keep[as.integer(row.names(ecoFB[ecoFB$Count>=best-1,]))]<-
ifelse(AllTriplets$keep[as.integer(row.names(ecoFB[ecoFB$Count>=best-
1,]))]==1,1,ifelse(AllTriplets$keep[as.integer(row.names(ecoFB[ecoFB$Count>=best-
1,]))]==2,2,ifelse(AllTriplets$keep[as.integer(row.names(ecoFB[ecoFB$Count>=best-
```

```
1,]))]==3,3,ifelse(AllTriplets$keep[as.integer(row.names(ecoFB[ecoFB$Count>=best-
1,]))]==4,4,5))))
      }
    }
  }

  if(NROW(cc)<=unq){AllTriplets$keep[as.integer(row.names(cc))]<-1 }

  ##cc
  cc<-AllTriplets[AllTriplets$Document==sentences$Document[i] &
AllTriplets$Sentence==sentences$Sentence[i],]
  cc[order(cc$keep),]

}



####adds the 'keep' scores to the file and also removes 'blanks' (non-Triplets)
write.table(AllTriplets,file = paste(
directory,"Triplets_SubClass_Clean.csv",sep=""),sep=",",row.names=FALSE,col.names=TRUE,app
end=FALSE)

#####################This next step uses the Keep Scores in the previous step to generate a
draft Triplets Master File
####This needs to be run to generate the Triplets Master, but you can choose to ignore the
likelihoods it generates; they are only there to help with QC
####This step recalculates the document counts based on the keep scores (three different ways)
####for a given Triplet, the "weight of evidence" is scored based on the frequency of that
category (B, F, or E) in the data; i.e., which combinations are most common vs. rare
### these weights are multiplied to get a probability that a triplet is 'valid' (based on the
frequencies in the documents)
### these 'valid' probabilities are multiplied by the document counts to get an 'importance'
score called 'true count'
### these are used to give a "combined score" 100=it was important in the most likely
sentences, 10=it was important in the probably likely sentences, 1 = it was important
somewhere in all the sentences
### these get added together to give a combined score based on the keep scores ...
### such that 111 or 100 means this is a highly likely true triplet because it was identified as the
most likely for each sentence, and it had a high frequency of occurrence
### such that 11 or 10 means this is a likely true triplet because it was identified for sentences
as probably likely, and it had a high frequency of occurrence
### such that 1 means this is maybe a true triplet because it had a high frequency of
occurrence, but was never identified as the most likely for a given sentence
### such that 0 means this is probably not a true triplet because it had a low frequency of
occurrence, and was never identified as the most likely for a given sentence
```

```
### the Tentative "FINAL" score is assigned as having a combined score>1, BUT THESE NEED TO
BE QCd!!!!   FINAL =1 means true triplet, Final =0 means not a true triplet FOR Further Analysis
and Generating Final Counts


####First, generate a table of what the triplets are#####
directory <- "/Users/chloejackson/Documents/Priority_FEGS/R/outputs/"
AllTriplets<- read.csv( file = paste(
directory,"Triplets_SubClass_Clean.csv",sep=""),sep=",",header=TRUE,as.is=TRUE,fill=FALSE)

for(KR in 1:3){

  if(KR==1){Triplets_sc<- AllTriplets[AllTriplets$keep==1,]}  ###only counts triplets from most
likely sentences
  if(KR==2){Triplets_sc<- AllTriplets[AllTriplets$keep>=1,]}  ###count triplets from probably likely
sentences
  if(KR==3){Triplets_sc<- AllTriplets[AllTriplets$keep>=0,]}  ###counts triplets from all sentences
(ignores keep)

  triplets<-
aggregate(Triplets_sc$keep,by=list(Triplets_sc$Beneficiary_SubClass,Triplets_sc$Ecosystem_Sub
Class,Triplets_sc$FEGS_SubClass),FUN=sum)
  names(triplets)<-c("Beneficiary_SubClass","Ecosystem_SubClass","FEGS_SubClass","potential")

  Triplets_sc<-
merge(AllTriplets,triplets,by.x=c("Beneficiary_SubClass","Ecosystem_SubClass","FEGS_SubClass"
),by.y=c("Beneficiary_SubClass","Ecosystem_SubClass","FEGS_SubClass"),all=TRUE)

  trips_est<-
aggregate(Triplets_sc$Line_Number,by=list(Triplets_sc$Beneficiary_SubClass,Triplets_sc$Ecosys
tem_SubClass,Triplets_sc$FEGS_SubClass,Triplets_sc$Document),FUN=length)
  names(trips_est)<-
c("Beneficiary_Class","Ecosystem_Class","FEGS_Class","Document","Line_Number")
  triplets_estuary<-
aggregate(trips_est$Line_Number,by=list(trips_est$Beneficiary_Class,trips_est$Ecosystem_Clas
s,trips_est$FEGS_Class),FUN=length)
  triplets_estuary_total<-
aggregate(trips_est$Line_Number,by=list(trips_est$Beneficiary_Class,trips_est$Ecosystem_Clas
s,trips_est$FEGS_Class),FUN=sum)
  triplets_estuary<-cbind(triplets_estuary,triplets_estuary_total$x)
  names(triplets_estuary)<-
c("Beneficiary_SubClass","Ecosystem_SubClass","FEGS_SubClass","Count","Hits")
```

```
  trips_cp<-
aggregate(Triplets_sc$Consider_Paragraph,by=list(Triplets_sc$Beneficiary_SubClass,Triplets_sc$
Ecosystem_SubClass,Triplets_sc$FEGS_SubClass,Triplets_sc$Document),FUN=min)
  names(trips_cp)<-
c("Beneficiary_Class","Ecosystem_Class","FEGS_Class","Document","Consider_Paragraph")
  triplets_cp<-
aggregate(trips_cp$Consider_Paragraph,by=list(trips_est$Beneficiary_Class,trips_est$Ecosyste
m_Class,trips_est$FEGS_Class),FUN=min)
  triplets_estuary<-cbind(triplets_estuary,triplets_cp$x)
  names(triplets_estuary)<-
c("Beneficiary_SubClass","Ecosystem_SubClass","FEGS_SubClass","Count","Hits","Consider_Par
agraph")


  triplets_estuary$example1<-triplets_estuary$Hits
  triplets_estuary$example2<-triplets_estuary$Hits

  for(i in 1:nrow(triplets_estuary)){
    triplet_matches<-
Triplets_sc[Triplets_sc$Beneficiary_SubClass==triplets_estuary$Beneficiary_SubClass[i],]
    triplet_matches<-
triplet_matches[triplet_matches$Ecosystem_SubClass==triplets_estuary$Ecosystem_SubClass[i]
,]
    triplet_matches<-
triplet_matches[triplet_matches$FEGS_SubClass==triplets_estuary$FEGS_SubClass[i],]
    triplets_estuary$example1[i]<-
paste(triplet_matches$Paragraph_Text[floor(runif(1,1,nrow(triplet_matches)+1))])
    triplet_matches<-
triplet_matches[triplet_matches$Paragraph_Text!=triplets_estuary$example1[i],]
    triplets_estuary$example2[i]<-
paste(triplet_matches$Paragraph_Text[floor(runif(1,1,nrow(triplet_matches)+1))])
  }

  AllTriplets_Counts<-
triplets_estuary[order(paste(triplets_estuary$FEGS_SubClass,triplets_estuary$Ecosystem_SubCl
ass)),]

  AllTriplets_Counts<-
merge(AllTriplets_Counts,triplets,by.x=c("Beneficiary_SubClass","Ecosystem_SubClass","FEGS_S
ubClass"),by.y=c("Beneficiary_SubClass","Ecosystem_SubClass","FEGS_SubClass"),all=TRUE)
  AllTriplets_FullList<-AllTriplets_Counts

  AllTriplets_Counts<- AllTriplets_Counts[is.na(AllTriplets_Counts$potential)==FALSE,]
  Triplets_sc<- Triplets_sc[is.na(Triplets_sc$potential)==FALSE,]
```

```
  ###count likelihoods of certain combos
  bens<-sort(unique(AllTriplets_Counts$Beneficiary_SubClass))
  envs<-sort(unique(AllTriplets_Counts$Ecosystem_SubClass))
  fegs<-sort(unique(AllTriplets_Counts$FEGS_SubClass))

  ###For a given FEG, calculate the weight of evidence for a Ben/Eco pair
  B<-
aggregate(AllTriplets_Counts$Count,by=list(AllTriplets_Counts$Beneficiary_SubClass),FUN=sum
)
  B$x=B$x/sum(AllTriplets_Counts$Count)

  E<-
aggregate(AllTriplets_Counts$Count,by=list(AllTriplets_Counts$Ecosystem_SubClass),FUN=sum)
  E$x=E$x/sum(AllTriplets_Counts$Count)

  F<-aggregate(AllTriplets_Counts$Count,by=list(AllTriplets_Counts$FEGS_SubClass),FUN=sum)
  F$x=F$x/sum(AllTriplets_Counts$Count)

  BF<-
aggregate(AllTriplets_Counts$Count,by=list(AllTriplets_Counts$Beneficiary_SubClass,AllTriplets_
Counts$FEGS_SubClass),FUN=sum)
  BF$x=BF$x/sum(AllTriplets_Counts$Count)
  FE<-
aggregate(AllTriplets_Counts$Count,by=list(AllTriplets_Counts$Ecosystem_SubClass,AllTriplets_
Counts$FEGS_SubClass),FUN=sum)
  FE$x=FE$x/sum(AllTriplets_Counts$Count)
  EB<-
aggregate(AllTriplets_Counts$Count,by=list(AllTriplets_Counts$Ecosystem_SubClass,AllTriplets_
Counts$Beneficiary_SubClass),FUN=sum)
  EB$x=EB$x/sum(AllTriplets_Counts$Count)

  BF_count<-
nrow(aggregate(Triplets_sc$Line_Number,by=list(Triplets_sc$Beneficiary_SubClass,Triplets_sc$F
EGS_SubClass,Triplets_sc$Document),FUN=length))
  FE_count<-
nrow(aggregate(Triplets_sc$Line_Number,by=list(Triplets_sc$Ecosystem_SubClass,Triplets_sc$F
EGS_SubClass,Triplets_sc$Document),FUN=length))
  EB_count<-
nrow(aggregate(Triplets_sc$Line_Number,by=list(Triplets_sc$Beneficiary_SubClass,Triplets_sc$E
cosystem_SubClass,Triplets_sc$Document),FUN=length))
```

```
  TripletMaster<-
data.frame("Beneficiary_SubClass"=rep(0,NROW(envs)*NROW(bens)*NROW(fegs)),"Ecosystem
_SubClass"=rep(0,NROW(envs)*NROW(bens)*NROW(fegs)),"FEGS_SubClass"=rep(0,NROW(envs
)*NROW(bens)*NROW(fegs)),"valid"=rep(0,NROW(envs)*NROW(bens)*NROW(fegs)),"BFvalid"=
rep(0,NROW(envs)*NROW(bens)*NROW(fegs)),"FEvalid"=rep(0,NROW(envs)*NROW(bens)*NR
OW(fegs)),"EBvalid"=rep(0,NROW(envs)*NROW(bens)*NROW(fegs)))

  myrow=1;
  for(i in 1:NROW(bens)){
    for(j in 1:NROW(envs)){
      for(k in 1:NROW(fegs)){
        TripletMaster[myrow,1]<-bens[i]
        TripletMaster[myrow,2]<-envs[j]
        TripletMaster[myrow,3]<-fegs[k]
        if(NROW(BF$x[BF$Group.1==bens[i] & BF$Group.2==fegs[k]]*FE$x[FE$Group.2==fegs[k] &
FE$Group.1==envs[j]]*EB$x[EB$Group.1==envs[j] & EB$Group.2==bens[i]])>0){
          TripletMaster[myrow,4]<-BF$x[BF$Group.1==bens[i] &
BF$Group.2==fegs[k]]*FE$x[FE$Group.2==fegs[k] &
FE$Group.1==envs[j]]*EB$x[EB$Group.1==envs[j] &
EB$Group.2==bens[i]]/(F$x[F$Group.1==fegs[k]]*E$x[E$Group.1==envs[j]]*B$x[B$Group.1==be
ns[i]])
          TripletMaster[myrow,5]<-BF$x[BF$Group.1==bens[i] & BF$Group.2==fegs[k]]
          TripletMaster[myrow,6]<-FE$x[FE$Group.2==fegs[k] & FE$Group.1==envs[j]]
          TripletMaster[myrow,7]<-EB$x[EB$Group.1==envs[j] & EB$Group.2==bens[i]]
        }
        myrow<-myrow+1
      }}}

  TripletMaster_reduced<-
merge(TripletMaster,AllTriplets_Counts,by.x=c("Beneficiary_SubClass","Ecosystem_SubClass","F
EGS_SubClass"),by.y=c("Beneficiary_SubClass","Ecosystem_SubClass","FEGS_SubClass"))
  TripletMaster_reduced$TrueCount<-
TripletMaster_reduced$valid*sum(AllTriplets_Counts$Count)
  TripletMaster_reduced$TrueHits<-TripletMaster_reduced$valid*sum(AllTriplets_Counts$Hits)

  triplets<-
aggregate(AllTriplets$keep,by=list(AllTriplets$Beneficiary_SubClass,AllTriplets$Ecosystem_SubCl
ass,AllTriplets$FEGS_SubClass),FUN=mean)
  names(triplets)<-c("Beneficiary_SubClass","Ecosystem_SubClass","FEGS_SubClass","Keep")

  if(KR==1){TripletMaster_FullList1<-
merge(AllTriplets_FullList,TripletMaster_reduced,by.x=c("Beneficiary_SubClass","Ecosystem_Su
bClass","FEGS_SubClass","Count","Hits","Consider_Paragraph","example1","example2","potenti
```

```
al"),by.y=c("Beneficiary_SubClass","Ecosystem_SubClass","FEGS_SubClass","Count","Hits","Cons
ider_Paragraph","example1","example2","potential"),all=TRUE)}
  if(KR==2){TripletMaster_FullList12<-
merge(AllTriplets_FullList,TripletMaster_reduced,by.x=c("Beneficiary_SubClass","Ecosystem_Su
bClass","FEGS_SubClass","Count","Hits","Consider_Paragraph","example1","example2","potenti
al"),by.y=c("Beneficiary_SubClass","Ecosystem_SubClass","FEGS_SubClass","Count","Hits","Cons
ider_Paragraph","example1","example2","potential"),all=TRUE)}
  if(KR==3){TripletMaster_FullList012<-
merge(AllTriplets_FullList,TripletMaster_reduced,by.x=c("Beneficiary_SubClass","Ecosystem_Su
bClass","FEGS_SubClass","Count","Hits","Consider_Paragraph","example1","example2","potenti
al"),by.y=c("Beneficiary_SubClass","Ecosystem_SubClass","FEGS_SubClass","Count","Hits","Cons
ider_Paragraph","example1","example2","potential"),all=TRUE)}

}  ###end KR

TripletMaster_FullList<-
merge(TripletMaster_FullList012,TripletMaster_FullList12[,c("Beneficiary_SubClass","Ecosystem
_SubClass","FEGS_SubClass","TrueCount")],by.x=c("Beneficiary_SubClass","Ecosystem_SubClass
","FEGS_SubClass"),by.y=c("Beneficiary_SubClass","Ecosystem_SubClass","FEGS_SubClass"),all=
TRUE)
TripletMaster_FullList<-
merge(TripletMaster_FullList,TripletMaster_FullList1[,c("Beneficiary_SubClass","Ecosystem_Sub
Class","FEGS_SubClass","TrueCount")],by.x=c("Beneficiary_SubClass","Ecosystem_SubClass","FE
GS_SubClass"),by.y=c("Beneficiary_SubClass","Ecosystem_SubClass","FEGS_SubClass"),all=TRUE
)
names(TripletMaster_FullList)<-
c("Beneficiary_SubClass","Ecosystem_SubClass","FEGS_SubClass","Count","Hits","Consider_Par
agraph","example1","example2","potential","valid","BFvalid","FEvalid","EBvalid","TrueCount01
2","TrueHits","TrueCount12","TrueCount1")

TripletMaster_FullList$Combined<-
100*ifelse(ifelse(is.na(TripletMaster_FullList$TrueCount1)==TRUE,0,TripletMaster_FullList$True
Count1)>0,1,0) +
10*ifelse(ifelse(is.na(TripletMaster_FullList$TrueCount12)==TRUE,0,TripletMaster_FullList$True
Count12)>1,1,0) +
1*ifelse(ifelse(is.na(TripletMaster_FullList$TrueCount012)==TRUE,0,TripletMaster_FullList$True
Count012)>1,1,0)
TripletMaster_FullList$FINAL<-ifelse(TripletMaster_FullList$Combined>1,1,0)

write.table(TripletMaster_FullList,file=paste(directory,"TripletsMaster_Embayments.csv",sep=""
), sep = ",", row.names=FALSE,col.names=TRUE,append=FALSE)

######Note for QC all the "valid" probabilities sum to 1 and the TrueCounts sum to the Total
Counts in TripletMaster.csv
```

```
#########TEMPORARILY ASSIGN OLD NEP Triplet Master FINAL values#################
#########TEMPORARILY ASSIGN OLD NEP Triplet Master FINAL values#################
#########TEMPORARILY ASSIGN OLD NEP Triplet Master FINAL values#################
#########TEMPORARILY ASSIGN OLD NEP Triplet Master FINAL values#################
################Compare Final from Original NEP document analysis
################Compare Final from Original NEP document analysis
################Compare Final from Original NEP document analysis
library(readxl)
directory <- "/Users/chloejackson/Documents/Priority_FEGS/R/outputs/"
Old_NEP_FEGS<-
read_excel("/Users/chloejackson/Documents/Priority_FEGS/R/outputs/Triplets_Master_OldNE
P_to_NESCS.xlsx", sheet = "Triplet_NEP_Aquatic_Combined")
NESCS_words<-
read_excel("/Users/chloejackson/Documents/Priority_FEGS/R/outputs/Triplets_Master_OldNE
P_to_NESCS.xlsx", sheet = "Translator")
Old_NEP_FEGS<-as.data.frame(Old_NEP_FEGS)
NESCS_words<-as.data.frame(NESCS_words)
TripletMaster_reduced<-
read.csv(file=paste(directory,"TripletsMaster_Embayments.csv",sep=""), sep =
",",header=TRUE,as.is=TRUE,fill=FALSE)
names(TripletMaster_reduced)[NCOL(TripletMaster_reduced)]<-"TripMast_Final"

TripletMaster_reduced$Old_NEP_FEGS_Final_Aquatic<-rep(0,NROW(TripletMaster_reduced))
for(i in 1:NROW(TripletMaster_reduced)){

if(NROW(Old_NEP_FEGS$FINAL[Old_NEP_FEGS$FEGS_NEP==(NESCS_words$NEP_Class[NESCS_
words$SubClass==TripletMaster_reduced$FEGS_SubClass[i]]) &
Old_NEP_FEGS$Ben_NEP==(NESCS_words$NEP_Class[NESCS_words$SubClass==TripletMaster_
reduced$Beneficiary_SubClass[i]])])>0)){
   TripletMaster_reduced$Old_NEP_FEGS_Final_Aquatic[i]<-
Old_NEP_FEGS$FINAL[Old_NEP_FEGS$FEGS_NEP==(NESCS_words$NEP_Class[NESCS_words$Su
bClass==TripletMaster_reduced$FEGS_SubClass[i]]) &
Old_NEP_FEGS$Ben_NEP==(NESCS_words$NEP_Class[NESCS_words$SubClass==TripletMaster_
reduced$Beneficiary_SubClass[i]])]
  }
}
TripletMaster_reduced$FINAL<-TripletMaster_reduced$Old_NEP_FEGS_Final_Aquatic

write.table(TripletMaster_reduced,file=paste(directory,"TripletsMaster_Embayments_reconcile
d.csv",sep=""), sep = ",", row.names=FALSE,col.names=TRUE,append=FALSE)

###############STOP HERE########################################
###############STOP HERE########################################
```

```
###############STOP HERE########################################
###############STOP HERE########################################

################CHECK The Triplets Master befor proceeding
further################################
################CHECK The Triplets Master befor proceeding
further################################
################CHECK The Triplets Master befor proceeding
further################################
################CHECK The Triplets Master befor proceeding
further################################
################CHECK The Triplets Master befor proceeding
further################################

################Set FINAL = 0 if the triplet is
nonsensical################################

###### the Tentative "FINAL" score in TripletsMaster is assigned as having a combined score>1,
BUT THESE NEED TO BE QCd!!!!
#####FINAL =1 means true triplet, Final =0 means not a true triplet FOR Further Analysis and
Generating Final Counts
#########ReconcileTriplets_MassBaysBCG.R   can be used to merge with old NEP version  and
then add any new ones

###This produces graphs and summary information AFTER running Triplets_Counter_FEGS.R
###This produces graphs and summary information AFTER running Triplets_Counter_FEGS.R
###This produces graphs and summary information AFTER running Triplets_Counter_FEGS.R
###This produces graphs and summary information AFTER running Triplets_Counter_FEGS.R


#############Start here AFTER Triplets Master has been QCd###########################
#############Start here AFTER Triplets Master has been QCd###########################
#################################################
####Discard any sentences that are keep=0, or don't have a likelihood > 1;  keep only "true
triplets"
###keep = 0 may come back in
###TripletMaster_reduced<-
read.csv(file=paste(directory,"TripletsMaster_MassBayAllHabs_Reconciled.csv",sep=""), sep =
",",header=TRUE,as.is=TRUE,fill=FALSE)

library(readxl)
library(ggplot2)
library(stringr)
```

```
directory <- "/Users/chloejackson/Documents/Priority_FEGS/R/outputs/"

Plans<-read.csv("/Users/chloejackson/Documents/Priority_FEGS/R/LookupTable.csv")

Friendly_Names<-
read_excel("/Users/chloejackson/Documents/Priority_FEGS/R/Friendly_Names.xlsx", sheet =
"Sheet1")

TripletMaster_reduced<-
read.csv(file=paste(directory,"TripletsMaster_Embayments_reconciled.csv",sep=""), sep =
",",header=TRUE,as.is=TRUE,fill=FALSE)
AllTriplets<- read.csv( file = paste(
directory,"Triplets_SubClass_Clean.csv",sep=""),sep=",",header=TRUE,as.is=TRUE,fill=FALSE)

Plans$Filename<-paste(Plans$Document,".txt",sep="")
Plans$Region<-gsub("-","_",Plans$Region)
Plans$Organization_Class<-gsub("-","_",Plans$Organization_Class)

##Join the sentences to the Counts & the Lookup Table
AllTriplets<-AllTriplets[AllTriplets$FEGS_SubClass!="blank",]
AllTriplets<-
merge(AllTriplets,TripletMaster_reduced[,c("Beneficiary_SubClass","Ecosystem_SubClass","FEG
S_SubClass","Count","TrueCount1","TrueCount12","TrueCount012","FINAL")],by.x=c("Beneficiar
y_SubClass","Ecosystem_SubClass","FEGS_SubClass"),by.y=c("Beneficiary_SubClass","Ecosystem
_SubClass","FEGS_SubClass"))
AllTriplets<-merge(AllTriplets,Plans,by.x="Document",by.y="Filename",all.x=FALSE,all.y=FALSE)


##########DECIDE WHETHER TO Subset to only "True" Triplets based on Triplet Master?
###from Triplets_Counter_FEGS.R  keep=0 keeps ALL triplets assigned to sentence; keep=1
keeps only top triplets assigned to each sentence; keep=2 keeps only the top of the top triplets
assigned to each sentence
###FINAL =1 are the QCd 'true' triplets; to be 100% inclusive set FINAL>=0
########## To be most inclusive, set keep>=0; FINAL>=0
########## To be most restrictive, set keep>=2; FINAL>=1
AllTriplets<-AllTriplets[AllTriplets$keep>=0,]
AllTriplets<-AllTriplets[AllTriplets$FINAL>=0,]


########DECIDE WHETHER TO i) KEEP Only SENTENCES, ii) SENTENCES +- 1 Sentence, OR iii)
keep SENTENCES +- 2 Sentences
##AllTriplets<-AllTriplets[AllTriplets$Consider_Paragraph==0,]   ####Keep sentences only (most
restrictive)
```

```
AllTriplets<-AllTriplets[(AllTriplets$Consider_Paragraph %in%
AllTriplets$Consider_Paragraph[grep(2,AllTriplets$Consider_Paragraph)])==FALSE,]
####Remove paragraph searches of plus/minus TWO sentences
##AllTriplets<-AllTriplets[AllTriplets$Consider_Paragraph>=0,]   ####Keep sentences & full
paragraphs (most broad)


####DECIDE WHETHER TO EXCLUDE Generic/Unknown "Unknown Beneficiary" or "FEGS" or
"Environment" paired as part of a triplet
#### Add comments in front of the line to put that subclass Back in, otherwise dropped
AllTriplets<-AllTriplets[AllTriplets$Beneficiary_SubClass!="Unknown Beneficiary",]
AllTriplets<-AllTriplets[AllTriplets$FEGS_SubClass!="FEGS",]
AllTriplets<-AllTriplets[AllTriplets$Ecosystem_SubClass!="Environment",]



###Replace Subclass Names with something more user friendly for graphing
##Set to "Friendly Name" or "Grouped Name"  (note 'groups' could be groups of only 1 if non-
groups are preferred
Friendly_Names<-as.data.frame(Friendly_Names)
##Friendly_Names$Combined_Name<-Friendly_Names$Friendly_Name_SubClass
####uncomment to replace with unique "Friendly_Name"
Friendly_Names$Combined_Name<-Friendly_Names$Grouped_Name  ####uncomment to
replace with grouped "Grouped_Name"


Friendly_Names<-Friendly_Names[Friendly_Names$Combined_Name!="NA" &
Friendly_Names$Combined_Name!="Skip",]

###Identify classes associated with each Grouped_Name; if groups split classes, then combine
classes<-unique(Friendly_Names[,c("Combined_Name","Class")]);
classes$CombinedClass<-classes$Class;
for(i in
1:NROW(classes)){classes$CombinedClass[classes$Combined_Name==classes$Combined_Nam
e[i]]<-
str_c(classes$Class[classes$Combined_Name==classes$Combined_Name[i]],collapse="/")}
classes<-
unique(data.frame("Combined_Name"=classes$Combined_Name,"Class"=classes$CombinedCla
ss))


newB<-
merge(AllTriplets,Friendly_Names,by.x="Beneficiary_SubClass",by.y="SubClass",all.x=TRUE,sort=
FALSE)
```

```
newF<-
merge(newB,Friendly_Names,by.x="FEGS_SubClass",by.y="SubClass",all.x=TRUE,sort=FALSE)
newE<-
merge(newF,Friendly_Names,by.x="Ecosystem_SubClass",by.y="SubClass",all.x=TRUE,sort=FALS
E)
AllTriplets<-newE
AllTriplets$B_SC<-AllTriplets$Beneficiary_SubClass
AllTriplets$F_SC<-AllTriplets$FEGS_SubClass
AllTriplets$E_SC<-AllTriplets$Ecosystem_SubClass
AllTriplets$Beneficiary_SubClass<-AllTriplets$Combined_Name.x
AllTriplets$FEGS_SubClass<-AllTriplets$Combined_Name.y
AllTriplets$Ecosystem_SubClass<-AllTriplets$Combined_Name


########Aggregate Counts and Graph
AllTriplets_Full<-AllTriplets

BenList<-unique(AllTriplets$Beneficiary_SubClass); BenList<-BenList[is.na(BenList)==FALSE]
FegList<-unique(AllTriplets$FEGS_SubClass); FegList<-FegList[is.na(FegList)==FALSE]
EcoList<-unique(AllTriplets$Ecosystem_SubClass); EcoList<-EcoList[is.na(EcoList)==FALSE]

##########CHANGE THIS TO GRAPH/SUMMARIZE ALL HABITATS, OR INDIVIDUAL Habitats
#####copy/paste output to a separate labelled folder or it may get overwritten before
rerunning
#########Must match names in "friendly names" or "grouped names" whichever was used
#####"econame" gives a short code for graphing and naming files

for(el in 0:0){   ###set to 0 to run all ecosystems; OR Set to 1,2,3... matching order in EcoList to
run just One; OR set undesired Ecosystems to NA or "Skip" in Friendly Names

  if(el>0){EcoList2<-EcoList[el]; econame<-EcoList[el]}
  if(el==0){EcoList2<-EcoList; econame<-"All_Habs"}

  for(cat in 0:3){
    ###########DECIDE what level you want to summarize
    ###########copy/paste the one you want at the bottom of this list; change and rerun code
do graph something different
    ##cat=0  ####This summarizes across All Docs regardless of Region or Organization (for each
habitat in EcoList)
    ##cat=1  ####This summarizes by Region  (for each habitat in EcoList)
    ##cat=2  ####This summarizes by Organization Class (for each habitat in EcoList)
    ##cat=3  ####This summarizes by Region x Organization Class (for each habitat in EcoList)

    AllTriplets<-AllTriplets_Full
```

```
   if(cat==0){AllTriplets$Category<-rep("All_Docs",NROW(AllTriplets$Region)); category<-
"All_Docs";} ##category is any friendly name for graphing/file naming
   if(cat==1){AllTriplets$Category<-AllTriplets$Region; category<-"Region";} ##category is any
friendly name for graphing/file naming
   if(cat==2){AllTriplets$Category<-AllTriplets$Organization; category<-"Organization";}
   if(cat==3){AllTriplets$Category<-
paste(AllTriplets$Region,"_X_",AllTriplets$Organization,sep=""); category<-"RegXOrg";}

   dir.create(paste(directory,"Graphs\\",econame,"_",category,sep=""))

   AllTriplets<-AllTriplets[AllTriplets$Category!="NA",]  ###remove any NAs due to missing
lookup info or similar

   ###tally number of documents per category type
   Documents<-unique(AllTriplets[,c("Document","Category")])
   DocNum<-aggregate(Documents$Document,by=list(Documents$Category),FUN=length);
names(DocNum)<-c("Category","DocumentNumber");
   DocNum
   CatList<-unique(AllTriplets$Category)


   ###########Fraction of Documents Mentioning each Habitat for each Category
   Triplets_sc<- AllTriplets[AllTriplets$Ecosystem_SubClass %in% EcoList2,]
   trips_est<-
aggregate(Triplets_sc$Line_Number,by=list(Triplets_sc$Ecosystem_SubClass,Triplets_sc$Categor
y,Triplets_sc$Document),FUN=length)
   names(trips_est)<-c("Ecosystem_SubClass","Category","Document","Line_Number")
   triplets_estuary<-
aggregate(trips_est$Line_Number,by=list(trips_est$Ecosystem_SubClass,trips_est$Category),FU
N=length,drop=FALSE)
   triplets_estuary_total<-
aggregate(trips_est$Line_Number,by=list(trips_est$Ecosystem_SubClass,trips_est$Category),FU
N=sum,drop=FALSE)
   triplets_estuary<-cbind(triplets_estuary,triplets_estuary_total$x)
   names(triplets_estuary)<-c("Ecosystem_SubClass","Category","Count","Hits")
   triplets_estuary$Count[is.na(triplets_estuary$Count)==TRUE]<-0
   triplets_estuary<-merge(triplets_estuary,DocNum)
   triplets_estuary$Freq<-triplets_estuary$Count/triplets_estuary$DocumentNumber
   EC_Scores<-
reshape(triplets_estuary[,c("Category","Ecosystem_SubClass","Count")],timevar="Ecosystem_S
ubClass",idvar=c("Category"),direction="wide")
   names(EC_Scores)<-gsub("Count.","",names(EC_Scores))

write.table(EC_Scores,file=paste(directory,"Graphs\\",econame,"_",category,"\\EcosystemsxCat
```

```
egory","_DocCount_",category,"_",econame,".csv",sep=""), sep = ",",
row.names=FALSE,col.names=TRUE,append=FALSE)


  ##########Just Calculate Fraction of Docs that mention FEGS for each Ecosystem
  if(cat>0){
    Triplets_sc<- AllTriplets[AllTriplets$Ecosystem_SubClass %in% EcoList2,]
    trips_est<-
aggregate(Triplets_sc$Line_Number,by=list(Triplets_sc$Ecosystem_SubClass,Triplets_sc$FEGS_S
ubClass,Triplets_sc$Category,Triplets_sc$Document),FUN=length)
    names(trips_est)<-
c("Ecosystem_SubClass","FEGS_SubClass","Category","Document","Line_Number")
    triplets_estuary<-
aggregate(trips_est$Line_Number,by=list(trips_est$Ecosystem_SubClass,trips_est$FEGS_SubCla
ss,trips_est$Category),FUN=length,drop=FALSE)
    triplets_estuary_total<-
aggregate(trips_est$Line_Number,by=list(trips_est$Ecosystem_SubClass,trips_est$FEGS_SubCla
ss,trips_est$Category),FUN=sum,drop=FALSE)
    triplets_estuary<-cbind(triplets_estuary,triplets_estuary_total$x)
    names(triplets_estuary)<-
c("Ecosystem_SubClass","FEGS_SubClass","Category","Count","Hits")
    triplets_estuary$Count[is.na(triplets_estuary$Count)==TRUE]<-0
    triplets_estuary<-merge(triplets_estuary,DocNum)
    triplets_estuary$Freq<-triplets_estuary$Count/triplets_estuary$DocumentNumber
    FEC_Scores<-
reshape(triplets_estuary[,c("Category","Ecosystem_SubClass","FEGS_SubClass","Count")],timev
ar="FEGS_SubClass",idvar=c("Category","Ecosystem_SubClass"),direction="wide")
    names(FEC_Scores)<-gsub("Count.","",names(FEC_Scores))

write.table(FEC_Scores[,c("Category","Ecosystem_SubClass",sort(names(FEC_Scores[3:NCOL(FE
C_Scores)])))],file=paste(directory,"Graphs\\",econame,"_",category,"\\FEGSxCategoryxEcosyste
m","_DocCount_",category,"_",econame,".csv",sep=""), sep = ",",
row.names=FALSE,col.names=TRUE,append=FALSE)
  }
  ##########Beneficiary Frequency in each Ecosystem for Each Category;
  if(cat>0){
    Triplets_sc<- AllTriplets[AllTriplets$Ecosystem_SubClass %in% EcoList2,]
    trips_est<-
aggregate(Triplets_sc$Line_Number,by=list(Triplets_sc$Ecosystem_SubClass,Triplets_sc$Benefic
iary_SubClass,Triplets_sc$Category,Triplets_sc$Document),FUN=length)
    names(trips_est)<-
c("Ecosystem_SubClass","Beneficiary_SubClass","Category","Document","Line_Number")
```

```
    triplets_estuary<-
aggregate(trips_est$Line_Number,by=list(trips_est$Ecosystem_SubClass,trips_est$Beneficiary_SubClass,trips_est$Category),FUN=length,drop=FALSE)
    triplets_estuary_total<-
aggregate(trips_est$Line_Number,by=list(trips_est$Ecosystem_SubClass,trips_est$Beneficiary_SubClass,trips_est$Category),FUN=sum,drop=FALSE)
    triplets_estuary<-cbind(triplets_estuary,triplets_estuary_total$x)
    names(triplets_estuary)<-
c("Ecosystem_SubClass","Beneficiary_SubClass","Category","Count","Hits")
    triplets_estuary$Count[is.na(triplets_estuary$Count)==TRUE]<-0
    triplets_estuary<-merge(triplets_estuary,DocNum)
    triplets_estuary$Freq<-triplets_estuary$Count/triplets_estuary$DocumentNumber
    BEC_Scores<-
reshape(triplets_estuary[,c("Category","Ecosystem_SubClass","Beneficiary_SubClass","Count")],
timevar="Beneficiary_SubClass",idvar=c("Category","Ecosystem_SubClass"),direction="wide")
    names(BEC_Scores)<-gsub("Count.","",names(BEC_Scores))

write.table(BEC_Scores[,c("Category","Ecosystem_SubClass",sort(names(BEC_Scores[3:NCOL(BEC_Scores)])))],file=paste(directory,"Graphs\\",econame,"_",category,"\\BensxCategoryxEcosystem","_DocCount_",category,"_",econame,".csv",sep=""), sep = ",",
row.names=FALSE,col.names=TRUE,append=FALSE)
  }

  ####Total BEF counts across all categories###
  Triplets_sc<- AllTriplets[AllTriplets$Ecosystem_SubClass %in% EcoList2,]
  trips_est<-
aggregate(Triplets_sc$Line_Number,by=list(Triplets_sc$Category,Triplets_sc$Beneficiary_SubClass,Triplets_sc$Ecosystem_SubClass,Triplets_sc$FEGS_SubClass,Triplets_sc$Document),FUN=length)
  names(trips_est)<-
c("Category","Beneficiary_SubClass","Ecosystem_SubClass","FEGS_SubClass","Document","Line_Number")
  triplets_estuary<-
aggregate(trips_est$Line_Number,by=list(trips_est$Category,trips_est$Beneficiary_SubClass,trips_est$Ecosystem_SubClass,trips_est$FEGS_SubClass),FUN=length)
  triplets_estuary_total<-
aggregate(trips_est$Line_Number,by=list(trips_est$Category,trips_est$Beneficiary_SubClass,trips_est$Ecosystem_SubClass,trips_est$FEGS_SubClass),FUN=sum)
  triplets_estuary<-cbind(triplets_estuary,triplets_estuary_total$x)
  names(triplets_estuary)<-
c("Category","Beneficiary_SubClass","Ecosystem_SubClass","FEGS_SubClass","Count","Hits")
  triplets_estuary<-merge(triplets_estuary,DocNum)
  triplets_estuary$Freq<-triplets_estuary$Count/triplets_estuary$DocumentNumber
  triplets_estuary$Count[is.na(triplets_estuary$Count)==TRUE]<-0
```

```
   ##    triplets_estuary$example1<-triplets_estuary$Count
   ##    triplets_estuary$example2<-triplets_estuary$Count
   ##    for(i in 1:nrow(triplets_estuary)){
   ##              triplet_matches<-
Triplets_sc[Triplets_sc$Beneficiary_SubClass==triplets_estuary$Beneficiary_SubClass[i] &
Triplets_sc$Ecosystem_SubClass==triplets_estuary$Ecosystem_SubClass[i] &
Triplets_sc$FEGS_SubClass==triplets_estuary$FEGS_SubClass[i] &
Triplets_sc$Category==triplets_estuary$Category[i],]
   ##              bestest<-triplet_matches
   ##              triplets_estuary$example1[i]<-
paste(bestest$Paragraph_Text[floor(runif(1,1,nrow(bestest)+1))])
   ##              triplet_matches<-
triplet_matches[triplet_matches$Paragraph_Text!=triplets_estuary$example1[i],]
   ##              triplets_estuary$example2[i]<-
paste(triplet_matches$Paragraph_Text[floor(runif(1,1,nrow(triplet_matches)+1))])
   ##    }
   ##
   ##    AllTriplets_NEP_Counts<-
triplets_estuary[order(paste(triplets_estuary$Category,triplets_estuary$Beneficiary_SubClass,tr
iplets_estuary$FEGS_SubClass,triplets_estuary$Ecosystem_SubClass)),]
   ##    BenFegEco_Counts<-AllTriplets_NEP_Counts
   ##
write.table(BenFegEco_Counts,file=paste(directory,"Graphs\\",econame,"_",category,"\\BenFeg
EcoCat_DocCounts_",category,"_",econame,".csv",sep=""), sep = ",",
row.names=FALSE,col.names=TRUE,append=FALSE)

   BEFC_Scores<-
reshape(triplets_estuary[,c("Category","Ecosystem_SubClass","Beneficiary_SubClass","FEGS_Su
bClass","Count")],timevar="Category",idvar=c("Beneficiary_SubClass","Ecosystem_SubClass","F
EGS_SubClass"),direction="wide")
   names(BEFC_Scores)<-gsub("Count.","",names(BEFC_Scores))
   BEFC_Scores<-BEFC_Scores[order(BEFC_Scores$FEGS_SubClass),]
   BEFC_Scores<-BEFC_Scores[order(BEFC_Scores$Beneficiary_SubClass),]
   BEFC_Scores<-BEFC_Scores[order(BEFC_Scores$Ecosystem_SubClass),]
   BEFC_Scores[is.na(BEFC_Scores)]<-0  ##fill NA with 0 that are created because a Feg/Ben
combo wasn't mentioned (true zeros); needed for

   BEFC_Scores<-merge(BEFC_Scores,classes,by.x="FEGS_SubClass",by.y="Combined_Name")
   BEFC_Scores<-
merge(BEFC_Scores,classes,by.x="Beneficiary_SubClass",by.y="Combined_Name");
   BEFC_Scores<-
merge(BEFC_Scores,classes,by.x="Ecosystem_SubClass",by.y="Combined_Name")
```

```
   names(BEFC_Scores)<-
c("Ecosystem_SubClass","Beneficiary_SubClass","FEGS_SubClass","All_Docs","FEGS_Class","Ben
eficiary_Class","Ecosystem_Class")
   BEFC_Scores<-BEFC_Scores[order(BEFC_Scores$FEGS_SubClass),];BEFC_Scores<-
BEFC_Scores[order(BEFC_Scores$FEGS_Class),]
   BEFC_Scores<-BEFC_Scores[order(BEFC_Scores$Beneficiary_SubClass),];BEFC_Scores<-
BEFC_Scores[order(BEFC_Scores$Beneficiary_Class),]
   BEFC_Scores<-BEFC_Scores[order(BEFC_Scores$Ecosystem_SubClass),];BEFC_Scores<-
BEFC_Scores[order(BEFC_Scores$Ecosystem_Class),]

write.table(BEFC_Scores[,c("Ecosystem_Class","Ecosystem_SubClass","Beneficiary_Class","Bene
ficiary_SubClass","FEGS_Class","FEGS_SubClass","All_Docs")],file=paste(directory,"Graphs\\",ec
oname,"_",category,"\\BensxEcosystemxFegsxCategory","_DocCount_",category,"_",econame,".
csv",sep=""), sep = ",", row.names=FALSE,col.names=TRUE,append=FALSE)


   ###########Fraction of Documents Mentioning each Beneficiary for each Category
(regardless of habitat)
   Triplets_sc<- AllTriplets[AllTriplets$Ecosystem_SubClass %in% EcoList2,]
   trips_est<-
aggregate(Triplets_sc$Line_Number,by=list(Triplets_sc$Beneficiary_SubClass,Triplets_sc$Catego
ry,Triplets_sc$Document),FUN=length)
   names(trips_est)<-c("Beneficiary_SubClass","Category","Document","Line_Number")
   triplets_estuary<-
aggregate(trips_est$Line_Number,by=list(trips_est$Beneficiary_SubClass,trips_est$Category),F
UN=length,drop=FALSE)
   triplets_estuary_total<-
aggregate(trips_est$Line_Number,by=list(trips_est$Beneficiary_SubClass,trips_est$Category),F
UN=sum,drop=FALSE)
   triplets_estuary<-cbind(triplets_estuary,triplets_estuary_total$x)
   names(triplets_estuary)<-c("Beneficiary_SubClass","Category","Count","Hits")
   triplets_estuary$Count[is.na(triplets_estuary$Count)==TRUE]<-0
   triplets_estuary<-merge(triplets_estuary,DocNum)
   triplets_estuary$Freq<-triplets_estuary$Count/triplets_estuary$DocumentNumber
   BC_Scores<-
reshape(triplets_estuary[,c("Category","Beneficiary_SubClass","Count")],timevar="Beneficiary_
SubClass",idvar=c("Category"),direction="wide")
   names(BC_Scores)<-gsub("Count.","",names(BC_Scores))

write.table(BC_Scores,file=paste(directory,"Graphs\\",econame,"_",category,"\\BensxCategory"
,"_DocCount_",category,"_",econame,".csv",sep=""), sep = ",",
row.names=FALSE,col.names=TRUE,append=FALSE)

   ggplot(triplets_estuary, aes(fill=Category,y=Count, x=reorder(Beneficiary_SubClass,Count))) +
```

```
    geom_bar(position="stack", stat="identity") +  ##change fill to stack
    ggtitle(paste(category,econame)) +
    xlab("")+ylab(paste("Count of Docs Per Category"))+
    theme(axis.text.x = element_text(angle = 90,hjust=1,vjust=0.5,size=10)) +
    coord_flip()

ggsave(paste(directory,"Graphs\\",econame,"_",category,"\\BensXCategory_DocCount",categor
y,"_",econame,".jpg",sep=""),plot=last_plot(),device="jpeg",width=22,height=10,units="cm")


  ###########Fraction of Documents Mentioning each Beneficiary for each Habitat (regardless
of category)
  if(cat==0){
    Triplets_sc<- AllTriplets[AllTriplets$Ecosystem_SubClass %in% EcoList2,]
    trips_est<-
aggregate(Triplets_sc$Line_Number,by=list(Triplets_sc$Beneficiary_SubClass,Triplets_sc$Ecosys
tem_SubClass,Triplets_sc$Document),FUN=length)
    names(trips_est)<-
c("Beneficiary_SubClass","Ecosystem_SubClass","Document","Line_Number")
    triplets_estuary<-
aggregate(trips_est$Line_Number,by=list(trips_est$Beneficiary_SubClass,trips_est$Ecosystem_
SubClass),FUN=length,drop=FALSE)
    triplets_estuary_total<-
aggregate(trips_est$Line_Number,by=list(trips_est$Beneficiary_SubClass,trips_est$Ecosystem_
SubClass),FUN=sum,drop=FALSE)
    triplets_estuary<-cbind(triplets_estuary,triplets_estuary_total$x)
    names(triplets_estuary)<-c("Beneficiary_SubClass","Ecosystem_SubClass","Count","Hits")
    triplets_estuary$Count[is.na(triplets_estuary$Count)==TRUE]<-0
    triplets_estuary$DocumentNumber<-sum(DocNum$DocumentNumber)
    triplets_estuary$Freq<-triplets_estuary$Count/triplets_estuary$DocumentNumber
    BE_Scores<-
reshape(triplets_estuary[,c("Ecosystem_SubClass","Beneficiary_SubClass","Count")],timevar="B
eneficiary_SubClass",idvar=c("Ecosystem_SubClass"),direction="wide")
    names(BE_Scores)<-gsub("Count.","",names(BE_Scores))

write.table(BE_Scores,file=paste(directory,"Graphs\\",econame,"_",category,"\\BensxEcosystem
","_DocCount_",category,"_",econame,".csv",sep=""), sep = ",",
row.names=FALSE,col.names=TRUE,append=FALSE)

    ggplot(triplets_estuary, aes(fill=Ecosystem_SubClass,y=Count,
x=reorder(Beneficiary_SubClass,Count))) +
      geom_bar(position="stack", stat="identity") +  ##change fill to stack
      ggtitle(paste(category,econame)) +
      xlab("")+ylab(paste("Count of Docs Per Category"))+
```

```
      theme(axis.text.x = element_text(angle = 90,hjust=1,vjust=0.5,size=10)) +
      coord_flip()

ggsave(paste(directory,"Graphs\\",econame,"_",category,"\\BensXEcosystem_DocCount",categ
ory,"_",econame,".jpg",sep=""),plot=last_plot(),device="jpeg",width=22,height=10,units="cm")
   }


   ###########Fraction of Documents Mentioning each FEGS for each Category (regardless of
habitat)
   Triplets_sc<- AllTriplets[AllTriplets$Ecosystem_SubClass %in% EcoList2,]
   trips_est<-
aggregate(Triplets_sc$Line_Number,by=list(Triplets_sc$FEGS_SubClass,Triplets_sc$Category,Trip
lets_sc$Document),FUN=length)
   names(trips_est)<-c("FEGS_SubClass","Category","Document","Line_Number")
   triplets_estuary<-
aggregate(trips_est$Line_Number,by=list(trips_est$FEGS_SubClass,trips_est$Category),FUN=le
ngth,drop=FALSE)
   triplets_estuary_total<-
aggregate(trips_est$Line_Number,by=list(trips_est$FEGS_SubClass,trips_est$Category),FUN=su
m,drop=FALSE)
   triplets_estuary<-cbind(triplets_estuary,triplets_estuary_total$x)
   names(triplets_estuary)<-c("FEGS_SubClass","Category","Count","Hits")
   triplets_estuary$Count[is.na(triplets_estuary$Count)==TRUE]<-0
   triplets_estuary<-merge(triplets_estuary,DocNum)
   triplets_estuary$Freq<-triplets_estuary$Count/triplets_estuary$DocumentNumber
   FC_Scores<-
reshape(triplets_estuary[,c("Category","FEGS_SubClass","Count")],timevar="FEGS_SubClass",id
var=c("Category"),direction="wide")
   names(FC_Scores)<-gsub("Count.","",names(FC_Scores))

write.table(FC_Scores,file=paste(directory,"Graphs\\",econame,"_",category,"\\FEGSxCategory"
,"_DocCount_",category,"_",econame,".csv",sep=""), sep = ",",
row.names=FALSE,col.names=TRUE,append=FALSE)

   ggplot(triplets_estuary, aes(fill=Category,y=Count, x=reorder(FEGS_SubClass,Count))) +
     geom_bar(position="stack", stat="identity") +  ##change fill to stack
     ggtitle(paste(category,econame)) +
     xlab("")+ylab(paste("Count of Docs Per Category"))+
     theme(axis.text.x = element_text(angle = 90,hjust=1,vjust=0.5,size=10)) +
     coord_flip()

ggsave(paste(directory,"Graphs\\",econame,"_",category,"\\FEGSXCategory_DocCount",categor
y,"_",econame,".jpg",sep=""),plot=last_plot(),device="jpeg",width=22,height=10,units="cm")
```

```
  ###########Fraction of Documents Mentioning each FEGS for each Habitat (regardless of
category)
  if(cat==0){
    Triplets_sc<- AllTriplets[AllTriplets$Ecosystem_SubClass %in% EcoList2,]
    trips_est<-
aggregate(Triplets_sc$Line_Number,by=list(Triplets_sc$FEGS_SubClass,Triplets_sc$Ecosystem_S
ubClass,Triplets_sc$Document),FUN=length)
    names(trips_est)<-c("FEGS_SubClass","Ecosystem_SubClass","Document","Line_Number")
    triplets_estuary<-
aggregate(trips_est$Line_Number,by=list(trips_est$FEGS_SubClass,trips_est$Ecosystem_SubCla
ss),FUN=length,drop=FALSE)
    triplets_estuary_total<-
aggregate(trips_est$Line_Number,by=list(trips_est$FEGS_SubClass,trips_est$Ecosystem_SubCla
ss),FUN=sum,drop=FALSE)
    triplets_estuary<-cbind(triplets_estuary,triplets_estuary_total$x)
    names(triplets_estuary)<-c("FEGS_SubClass","Ecosystem_SubClass","Count","Hits")
    triplets_estuary$Count[is.na(triplets_estuary$Count)==TRUE]<-0
    triplets_estuary$DocumentNumber<-sum(DocNum$DocumentNumber)
    triplets_estuary$Freq<-triplets_estuary$Count/triplets_estuary$DocumentNumber
    FE_Scores<-
reshape(triplets_estuary[,c("Ecosystem_SubClass","FEGS_SubClass","Count")],timevar="FEGS_S
ubClass",idvar=c("Ecosystem_SubClass"),direction="wide")
    names(FE_Scores)<-gsub("Count.","",names(FE_Scores))

write.table(FE_Scores,file=paste(directory,"Graphs\\",econame,"_",category,"\\FEGSxEcosystem
","_DocCount_",category,"_",econame,".csv",sep=""), sep = ",",
row.names=FALSE,col.names=TRUE,append=FALSE)

    ggplot(triplets_estuary, aes(fill=Ecosystem_SubClass,y=Count,
x=reorder(FEGS_SubClass,Count))) +
     geom_bar(position="stack", stat="identity") +  ##change fill to stack
     ggtitle(paste(category,econame)) +
     xlab("")+ylab(paste("Counts of Docs Per Category"))+
     theme(axis.text.x = element_text(angle = 90,hjust=1,vjust=0.5,size=10)) +
     coord_flip()

ggsave(paste(directory,"Graphs\\",econame,"_",category,"\\FEGSXEcosystem_DocCount",categ
ory,"_",econame,".jpg",sep=""),plot=last_plot(),device="jpeg",width=22,height=10,units="cm")
  }
```

```
   ###########Fraction of Documents Mentioning each FEGS x Bens (regardless of category or Habitat)
   if(cat==0){
     Triplets_sc<- AllTriplets[AllTriplets$Ecosystem_SubClass %in% EcoList2,]
     trips_est<-
aggregate(Triplets_sc$Line_Number,by=list(Triplets_sc$FEGS_SubClass,Triplets_sc$Beneficiary_SubClass,Triplets_sc$Document),FUN=length)
     names(trips_est)<-c("FEGS_SubClass","Beneficiary_SubClass","Document","Line_Number")
     triplets_estuary<-
aggregate(trips_est$Line_Number,by=list(trips_est$FEGS_SubClass,trips_est$Beneficiary_SubClass),FUN=length,drop=FALSE)
     triplets_estuary_total<-
aggregate(trips_est$Line_Number,by=list(trips_est$FEGS_SubClass,trips_est$Beneficiary_SubClass),FUN=sum,drop=FALSE)
     triplets_estuary<-cbind(triplets_estuary,triplets_estuary_total$x)
     names(triplets_estuary)<-c("FEGS_SubClass","Beneficiary_SubClass","Count","Hits")
     triplets_estuary$Count[is.na(triplets_estuary$Count)==TRUE]<-0
     triplets_estuary$DocumentNumber<-sum(DocNum$DocumentNumber)
     triplets_estuary$Freq<-triplets_estuary$Count/triplets_estuary$DocumentNumber
     FB_Scores<-
reshape(triplets_estuary[,c("Beneficiary_SubClass","FEGS_SubClass","Count")],timevar="FEGS_SubClass",idvar=c("Beneficiary_SubClass"),direction="wide")
     names(FB_Scores)<-gsub("Count.","",names(FB_Scores))

     FB_Scores<-
merge(FB_Scores,classes,by.x="Beneficiary_SubClass",by.y="Combined_Name",all.x=TRUE,all.y=FALSE,no.dups=TRUE);
     FB_Scores<-
cbind(FB_Scores$Class,FB_Scores$Beneficiary_SubClass,FB_Scores[,2:(NCOL(FB_Scores)-1)])
     names(FB_Scores)[1:2]<-c("Beneficiary_Class","Beneficiary_SubClass")
     FB_Scores<-FB_Scores[order(FB_Scores$Beneficiary_SubClass),];FB_Scores<-
FB_Scores[order(FB_Scores$Beneficiary_Class),]

write.table(FB_Scores,file=paste(directory,"Graphs\\",econame,"_",category,"\\FEGSxBeneficiary","_DocCount_",category,"_",econame,".csv",sep=""), sep = ",",
row.names=FALSE,col.names=TRUE,append=FALSE)

     ggplot(triplets_estuary, aes(fill=FEGS_SubClass,y=Count,
x=reorder(Beneficiary_SubClass,Count))) +
       geom_bar(position="fill", stat="identity") +  ##change fill to stack
       ggtitle(paste(category,econame)) +
       xlab("")+ylab(paste("Counts of Docs Per Category"))+
       theme(axis.text.x = element_text(angle = 90,hjust=1,vjust=0.5,size=10)) +
```

```
    coord_flip()

ggsave(paste(directory,"Graphs\\",econame,"_",category,"\\FEGSXBeneficiary_DocCount",categ
ory,"_",econame,".jpg",sep=""),plot=last_plot(),device="jpeg",width=30,height=10,units="cm")
  }

  ###########Fraction of Documents Mentioning each FEGS x Bens (by category regardless of
Habitat)
  if(cat>0){
    Triplets_sc<- AllTriplets[AllTriplets$Ecosystem_SubClass %in% EcoList2,]
    trips_est<-
aggregate(Triplets_sc$Line_Number,by=list(Triplets_sc$FEGS_SubClass,Triplets_sc$Beneficiary_
SubClass,Triplets_sc$Category,Triplets_sc$Document),FUN=length)
    names(trips_est)<-
c("FEGS_SubClass","Beneficiary_SubClass","Category","Document","Line_Number")
    triplets_estuary<-
aggregate(trips_est$Line_Number,by=list(trips_est$FEGS_SubClass,trips_est$Beneficiary_SubCl
ass,trips_est$Category),FUN=length,drop=FALSE)
    triplets_estuary_total<-
aggregate(trips_est$Line_Number,by=list(trips_est$FEGS_SubClass,trips_est$Beneficiary_SubCl
ass,trips_est$Category),FUN=sum,drop=FALSE)
    triplets_estuary<-cbind(triplets_estuary,triplets_estuary_total$x)
    names(triplets_estuary)<-
c("FEGS_SubClass","Beneficiary_SubClass","Category","Count","Hits")
    triplets_estuary$Count[is.na(triplets_estuary$Count)==TRUE]<-0
    triplets_estuary$DocumentNumber<-sum(DocNum$DocumentNumber)
    triplets_estuary$Freq<-triplets_estuary$Count/triplets_estuary$DocumentNumber
    FBC_Scores<-
reshape(triplets_estuary[,c("Beneficiary_SubClass","FEGS_SubClass","Category","Count")],time
var="Category",idvar=c("Beneficiary_SubClass","FEGS_SubClass"),direction="wide")
    names(FBC_Scores)<-gsub("Count.","",names(FBC_Scores))

    FBC_Scores<-merge(FBC_Scores,classes,by.x="FEGS_SubClass",by.y="Combined_Name")
    FBC_Scores<-
merge(FBC_Scores,classes,by.x="Beneficiary_SubClass",by.y="Combined_Name");
    names(FBC_Scores)[c(NCOL(FBC_Scores)-1,NCOL(FBC_Scores))]<-
c("FEGS_Class","Beneficiary_Class")
    FBC_Scores<-FBC_Scores[order(FBC_Scores$FEGS_SubClass),];FBC_Scores<-
FBC_Scores[order(FBC_Scores$FEGS_Class),]
    FBC_Scores<-FBC_Scores[order(FBC_Scores$Beneficiary_SubClass),];FBC_Scores<-
FBC_Scores[order(FBC_Scores$Beneficiary_Class),]
    FBC_Scores<-
cbind(FBC_Scores$Beneficiary_Class,FBC_Scores$Beneficiary_SubClass,FBC_Scores$FEGS_Class,
FBC_Scores$FEGS_SubClass,FBC_Scores[,3:(NCOL(FBC_Scores)-2)])
```

```
      names(FBC_Scores)[1:4]<-
c("Beneficiary_Class","Beneficiary_SubClass","FEGS_Class","FEGS_SubClass")

write.table(FBC_Scores,file=paste(directory,"Graphs\\",econame,"_",category,"\\FEGSxBeneficia
ryxCat","_DocCount_",category,"_",econame,".csv",sep=""), sep = ",",
row.names=FALSE,col.names=TRUE,append=FALSE)

      ###HEATMAPS For Each Category
      for(ee in 1:NROW(CatList)){
if(class(try(NROW(FBC_Scores[,c(CatList[ee])]),silent=TRUE))!="try-error"){
        BF_Heatmap<-
reshape(FBC_Scores[,c("FEGS_SubClass","Beneficiary_SubClass",CatList[ee])],timevar="FEGS_Su
bClass",idvar=c("Beneficiary_SubClass"),direction="wide")
        names(BF_Heatmap)<-gsub(paste(CatList[ee],".",sep=""),"",names(BF_Heatmap))
        BF_Heatmap<-
BF_Heatmap[order(rowSums(BF_Heatmap[,2:NCOL(BF_Heatmap)]),decreasing=TRUE),c(1,1+or
der(colSums(BF_Heatmap[,2:NCOL(BF_Heatmap)]),decreasing=TRUE))]

write.table(BF_Heatmap,file=paste(directory,"Graphs\\",econame,"_",category,"\\Heatmap_Be
nxFeg","_DocCount_",category,"_",CatList[ee],".csv",sep=""), sep = ",",
row.names=FALSE,col.names=TRUE,append=FALSE)
      }}
    }

    ###########Fraction of Documents Mentioning each FEGS x Bens (by Habitat regardless of
Category)
    if(cat==0){
      Triplets_sc<- AllTriplets[AllTriplets$Ecosystem_SubClass %in% EcoList2,]
      trips_est<-
aggregate(Triplets_sc$Line_Number,by=list(Triplets_sc$FEGS_SubClass,Triplets_sc$Beneficiary_
SubClass,Triplets_sc$Ecosystem_SubClass,Triplets_sc$Document),FUN=length)
      names(trips_est)<-
c("FEGS_SubClass","Beneficiary_SubClass","Ecosystem_SubClass","Document","Line_Number")
      triplets_estuary<-
aggregate(trips_est$Line_Number,by=list(trips_est$FEGS_SubClass,trips_est$Beneficiary_SubCl
ass,trips_est$Ecosystem_SubClass),FUN=length,drop=FALSE)
      triplets_estuary_total<-
aggregate(trips_est$Line_Number,by=list(trips_est$FEGS_SubClass,trips_est$Beneficiary_SubCl
ass,trips_est$Ecosystem_SubClass),FUN=sum,drop=FALSE)
      triplets_estuary<-cbind(triplets_estuary,triplets_estuary_total$x)
      names(triplets_estuary)<-
c("FEGS_SubClass","Beneficiary_SubClass","Ecosystem_SubClass","Count","Hits")
      triplets_estuary$Count[is.na(triplets_estuary$Count)==TRUE]<-0
      triplets_estuary$DocumentNumber<-sum(DocNum$DocumentNumber)
```

```
    triplets_estuary$Freq<-triplets_estuary$Count/triplets_estuary$DocumentNumber
    FBE_Scores<-
reshape(triplets_estuary[,c("Beneficiary_SubClass","FEGS_SubClass","Ecosystem_SubClass","Co
unt")],timevar="Ecosystem_SubClass",idvar=c("Beneficiary_SubClass","FEGS_SubClass"),directio
n="wide")
    names(FBE_Scores)<-gsub("Count.","",names(FBE_Scores))

    FBE_Scores<-merge(FBE_Scores,classes,by.x="FEGS_SubClass",by.y="Combined_Name")
    FBE_Scores<-
merge(FBE_Scores,classes,by.x="Beneficiary_SubClass",by.y="Combined_Name");
    names(FBE_Scores)[c(NCOL(FBE_Scores)-1,NCOL(FBE_Scores))]<-
c("FEGS_Class","Beneficiary_Class")
    FBE_Scores<-FBE_Scores[order(FBE_Scores$FEGS_SubClass),];FBE_Scores<-
FBE_Scores[order(FBE_Scores$FEGS_Class),]
    FBE_Scores<-FBE_Scores[order(FBE_Scores$Beneficiary_SubClass),];FBE_Scores<-
FBE_Scores[order(FBE_Scores$Beneficiary_Class),]
    FBE_Scores<-
data.frame(FBE_Scores$Beneficiary_Class,FBE_Scores$Beneficiary_SubClass,FBE_Scores$FEGS_
Class,FBE_Scores$FEGS_SubClass,FBE_Scores[,names(FBE_Scores) %in% EcoList2])
    names(FBE_Scores)<-
c("Beneficiary_Class","Beneficiary_SubClass","FEGS_Class","FEGS_SubClass",EcoList2)

write.table(FBE_Scores,file=paste(directory,"Graphs\\",econame,"_",category,"\\FEGSxBeneficia
ryxEco","_DocCount_",category,"_",econame,".csv",sep=""), sep = ",",
row.names=FALSE,col.names=TRUE,append=FALSE)

    ###HEATMAPS For Each Ecosystem
    for(ee in 1:NROW(EcoList2)){
     BF_Heatmap<-
reshape(FBE_Scores[,c("FEGS_SubClass","Beneficiary_SubClass",EcoList2[ee])],timevar="FEGS_S
ubClass",idvar=c("Beneficiary_SubClass"),direction="wide")
     names(BF_Heatmap)<-gsub(paste(EcoList2[ee],".",sep=""),"",names(BF_Heatmap))
     BF_Heatmap<-
BF_Heatmap[order(rowSums(BF_Heatmap[,2:NCOL(BF_Heatmap)]),decreasing=TRUE),c(1,1+or
der(colSums(BF_Heatmap[,2:NCOL(BF_Heatmap)]),decreasing=TRUE))]

write.table(BF_Heatmap,file=paste(directory,"Graphs\\",econame,"_",category,"\\Heatmap_Be
nxFeg","_DocCount_",category,"_",EcoList2[ee],".csv",sep=""), sep = ",",
row.names=FALSE,col.names=TRUE,append=FALSE)
    }

   }

 }}
```
