# Supplementary material for: Identifying priority ecosystem services in tidal wetland restoration
Source: Front Ecol Evol. Author manuscript; Available in PMC 2025 Jul 7. (PMC11457110; doi:10.3389/fevo.2024.1260447)
Supplement: Supplement1 [file NIHMS2010490-supplement-Supplement1.zip › Table 2_Identifying priority ecosystem services in tidal wetland restoration.docx]

Table A. The top 25% of EEPs across regions and organizations. The numbers in the cells represent the percentage of documents in which an EEP was linked to the region/organization. The colors in the cells represent the percentile thresholds for the top 25% of EEPs within regions and organizations individually. For regions, the top 25% was based on the average frequency of each EEP subclass across the three regions. For organizations, the top 25% was based on the average frequency of each EEP subclass across the four organizations. The cells that are unhighlighted represent EEP subclasses that did not meet the top 25% threshold for either the regions or the organizations but did for the other.

| **EEP Class** | **EEP Subclass** | | | | **Regions** | | | | **Organizations** | | | |
| --- | --- | --- | --- | --- | --- | --- | --- | --- | --- | --- | --- | --- |
|  |  |  |  |  | **Pacific Northwest** | | **Northern Gulf of Mexico** | **Mid-Atlantic** | **Federal Agencies** | **State & Local Agencies** | **Wetland Conservation Organizations** | **Land Stewards** |
| Flora | Flora (general) | | | | 71 | | 74 | 78 | 45 | 71 | 70 | 89 |
|  | Commercially Important Flora | | | | 38 | | 59 | 52 | 40 | 29 | 40 | 73 |
| Fauna | Fauna (general) | | | | 87 | | 76 | 84 | 65 | 71 | 85 | 96 |
|  | Charismatic Fauna | | | | 51 | | 61 | 56 | 30 | 40 | 40 | 84 |
|  | Commercially Important Fauna | | | | 40 | | 67 | 52 | 35 | 36 | 40 | 79 |
|  | Edible Fauna | | | | 49 | | 80 | 64 | 55 | 42 | 55 | 89 |
|  | Fauna Community | | | | 44 | | 57 | 54 | 35 | 33 | 30 | 80 |
| Water | Water (general) | | | | 62 | | 59 | 66 | 35 | 62 | 45 | 79 |
|  | Water Quality | | | | 64 | | 74 | 84 | 45 | 71 | 75 | 88 |
|  | Water Quantity | | | | 73 | | 74 | 78 | 40 | 71 | 75 | 91 |
|  | Water Movement/Navigability | | | | 78 | | 76 | 70 | 50 | 71 | 70 | 88 |
| Multiple Ecosystem Components | Multiple Ecosystem Components (general) | | | | 60 | | 72 | 80 | 45 | 67 | 55 | 89 |
|  | Aesthetic Viewscapes | | | | 56 | | 41 | 60 | 30 | 38 | 25 | 82 |
|  | Naturalness | | | | 89 | | 91 | 98 | 80 | 91 | 95 | 98 |
|  | Open Land for Development | | | | 76 | | 76 | 84 | 55 | 69 | 80 | 95 |
| Regulating Services | Regulating Services (general) | | | | 73 | | 76 | 88 | 55 | 80 | 65 | 93 |
|  | Water Quality Regulation (Nutrients & Retention) | | | | 76 | | 76 | 90 | 55 | 78 | 75 | 95 |
|  | Soil & Sediment Regulation | | | | 56 | | 50 | 72 | 35 | 67 | 45 | 68 |
| Risk of Extreme Events | Risk of Flooding | | | | 71 | | 72 | 70 | 50 | 69 | 75 | 79 |
| **Regions** | | | **Organizations** | | |  |  |  |  |  |  |  |
| **Percentiles** | | **Color** | **Percentiles** | **Color** | |  |  |  |  |  |  |  |
| Less than 25% threshold | |  | Less than 25% threshold |  | |  |  |  |  |  |  |  |
| 0-25% | |  | 0-25% |  | |  |  |  |  |  |  |  |
| 25.1-50% | |  | 25.1-50% |  | |  |  |  |  |  |  |  |
| 50.1-75% | |  | 50.1-75% |  | |  |  |  |  |  |  |  |
| 75.1-95% | |  | 75.1-95% |  | |  |  |  |  |  |  |  |
| 95.1-100% | |  | 95.1-100% |  | |  |  |  |  |  |  |  |

Table B. The top 25% of EEPs across tidal wetland types. The numbers in the cells represent the percentage of documents in which the EEP was linked with the tidal wetland type. The colors in the cells represent the percentile thresholds for the top 25% of EEPs within tidal wetland types.

| **EEP Class** | | **EEP Subclass** | **Tidal Wetlands (general)** | **Emergent Wetlands (marsh)** | **Forested Wetlands** | **Scrub-Shrub Wetlands** |
| --- | --- | --- | --- | --- | --- | --- |
| Flora | | Flora (general) | 71 | 45 | 23 | 12 |
|  |  | Flora Community | 50 | 26 | 18 | 6 |
|  |  | Charismatic Flora | 48 | 22 | 13 | 6 |
| Fauna | | Fauna (general) | 78 | 50 | 31 | 13 |
|  |  | Charismatic Fauna | 51 | 28 | 14 | 4 |
|  |  | Commercially Important Fauna | 52 | 23 | 13 | 1 |
|  |  | Edible Fauna | 62 | 33 | 20 | 4 |
|  |  | Fauna Community | 47 | 26 | 11 | 4 |
| Water | | Water (general) | 60 | 26 | 18 | 5 |
|  |  | Water Quality | 70 | 29 | 11 | 1 |
|  |  | Water Quantity | 72 | 29 | 16 | 1 |
|  |  | Water Movement/Navigability | 71 | 38 | 26 | 6 |
| Multiple Ecosystem Components | | Multiple Ecosystem Components (general) | 67 | 22 | 15 | 1 |
|  |  | Naturalness | 91 | 57 | 41 | 17 |
|  |  | Open Land for Development* | 78 | 34 | 20 | 6 |
| Regulating Services | | Regulating Services (general) | 79 | 38 | 15 | 8 |
|  |  | Water Quality Regulation (Nutrients & Retention) | 79 | 32 | 19 | 4 |
| Risk of Extreme Events | | Risk of Flooding | 70 | 34 | 14 | 6 |
| **Percentiles** | **Color** |  |  |  |  |  |
| 0% |  |  |  |  |  |  |
| 0.01-25% |  |  |  |  |  |  |
| 25.1-50% |  |  |  |  |  |  |
| 50.1-75% |  |  |  |  |  |  |
| 75.1-95% |  |  |  |  |  |  |
| 95.1-100% |  |  |  |  |  |  |

Table C. The top 25% of beneficiaries across regions and organizations. The numbers in the cells represent the percentage of documents in which the beneficiary was linked with the region/organization. The colors in the cells represent the percentile thresholds for the top 25% of beneficiaries within regions and organizations independently. For regions, the top 25% was based on the average frequency of each beneficiary subclass across the three regions. For organizations, the top 25% was based on the average frequency of each beneficiary subclass across the four organizations. The percentiles are based on the top 25%. The cells that are unhighlighted represent beneficiary subclasses that did not meet the top 25% threshold for either the regions or the organizations but did for the other.

| **Beneficiary Class** | | **Beneficiary Subclass** | | | | **Regions** | | | | **Organizations** | | | |
| --- | --- | --- | --- | --- | --- | --- | --- | --- | --- | --- | --- | --- | --- |
|  |  |  |  |  |  | **Pacific Northwest** | | **Northern Gulf of Mexico** | **Mid-Atlantic** | **Federal Agencies** | **State & Local Agencies** | **Wetland Conservation Organizations** | **Land Stewards** |
| Agricultural | | Agriculture (general) | | | | 69 | | 37 | 82 | 55 | 56 | 55 | 75 |
| Commercial & Industrial | | Commercial Food Extractors & Fisheries | | | | 44 | | 80 | 58 | 50 | 36 | 55 | 88 |
|  |  | Commercial/Industrial Property Owners | | | | 60 | | 57 | 66 | 45 | 60 | 40 | 75 |
| Government, Municipal, Residential | | Government, Municipal, Residential (general) | | | | 82 | | 78 | 94 | 60 | 84 | 80 | 96 |
|  |  | Residential Property Owners | | | | 62 | | 74 | 76 | 50 | 71 | 50 | 86 |
|  |  | Public Property Owners | | | | 58 | | 63 | 48 | 40 | 56 | 55 | 63 |
| Commercial/Military Transportation | | Nonspecific Commercial/Military Transportation | | | | 58 | | 67 | 60 | 45 | 60 | 40 | 77 |
| Recreational | | Recreation (general) | | | | 53 | | 61 | 64 | 45 | 53 | 30 | 80 |
|  |  | Experiencers/Viewers | | | | 73 | | 67 | 86 | 50 | 73 | 55 | 95 |
| Learning | | Educators/Students | | | | 67 | | 70 | 76 | 40 | 64 | 60 | 91 |
|  |  | Researchers | | | | 78 | | 83 | 84 | 65 | 76 | 90 | 89 |
| Nonuse Value | | People Who Care (Existence) | | | | 84 | | 85 | 92 | 65 | 82 | 90 | 98 |
|  |  | People Who Care (Option/Bequest) | | | | 58 | | 76 | 78 | 45 | 64 | 70 | 86 |
| **Regions** | | | **Organizations** | | |  |  |  |  |  |  |  |  |
| **Percentiles** | | **Color** | **Percentiles** | **Color** | |  |  |  |  |  |  |  |  |
| Less than 25% threshold | |  | Less than 25% threshold |  | |  |  |  |  |  |  |  |  |
| 0-25% | |  | 0-25% |  | |  |  |  |  |  |  |  |  |
| 25.1-50% | |  | 25.1-50% |  | |  |  |  |  |  |  |  |  |
| 50.1-75% | |  | 50.1-75% |  | |  |  |  |  |  |  |  |  |
| 75.1-95% | |  | 75.1-95% |  | |  |  |  |  |  |  |  |  |
| 95.1-100% | |  | 95.1-100% |  | |  |  |  |  |  |  |  |  |

Table D. The top 25% of beneficiaries across tidal wetland types. The numbers in the cells represent the percentage of documents in which the beneficiary was linked with the tidal wetland type. The colors in the cells represent the percentile thresholds for the top 25% of EEPs within tidal wetland types.

| **Beneficiary Class** | | **Beneficiary Subclass** | **Tidal Wetlands (general)** | **Emergent Wetlands (marsh)** | **Forested Wetlands** | **Scrub-Shrub Wetlands** |
| --- | --- | --- | --- | --- | --- | --- |
| Agricultural | | Agriculture (general) | 60 | 18 | 9 | 4 |
| Commercial & Industrial | | Commercial Food Extractors & Fisheries | 57 | 26 | 14 | 3 |
| Government, Municipal, Residential | | Government, Municipal, Residential (general) | 79 | 47 | 30 | 7 |
|  |  | Residential Property Owners | 67 | 18 | 14 | 1 |
| Commercial/Military Transportation | | Nonspecific Commercial Transportation | 57 | 21 | 14 | 0 |
| Recreational | | Recreational (general) | 52 | 23 | 13 | 1 |
|  |  | Experiencers/Viewers | 72 | 34 | 21 | 6 |
|  |  | Recreational Boaters | 46 | 20 | 16 | 2 |
| Learning | | Educators/Students | 67 | 38 | 19 | 4 |
|  |  | Researchers | 76 | 48 | 26 | 10 |
| Nonuse Value | | People Who Care (Existence) | 86 | 54 | 39 | 15 |
|  |  | People Who Care (Option/Bequest) | 63 | 30 | 15 | 1 |
| **Percentiles** | **Color** |  |  |  |  |  |
| 0% |  |  |  |  |  |  |
| 0.01-25% |  |  |  |  |  |  |
| 25.1-50% |  |  |  |  |  |  |
| 50.1-75% |  |  |  |  |  |  |
| 75.1-95% |  |  |  |  |  |  |
| 95.1-100% |  |  |  |  |  |  |

Table E. Top 50% of EEP-by-beneficiary subclass combinations for all documents. The colors/numbers in the cells represent the percentage of documents that the EEP-by-beneficiary subclass combination showed up in. The percentiles are based on all of the documents.

| **EEP Subclass/Beneficiary Subclass** | People Who Care (Existence) | Government, Municipal, Residential (general) | Researchers | People Who Care (Option/Bequest) | Experiencers/Viewers | Educators/Students | Residential Property Owners | Recreation (general) | Commercial/Industrial Property Owners | Agriculture (general) | Nonspecific Commercial/Military Transportation | Commercial Food Extractors & Fisheries | Learning (general) | Recreational Boaters | Public Property Owners | Farmers | Recreational Fishermen | Nonspecific Inspirational | Transporters of People | Recreational Hunters | Humanity & Public Health | Commercial & Industrial (general) | Military & Coast Guard |
| --- | --- | --- | --- | --- | --- | --- | --- | --- | --- | --- | --- | --- | --- | --- | --- | --- | --- | --- | --- | --- | --- | --- | --- |
| Naturalness | 86 | 82 | 74 | 69 | 68 | 65 | 57 | 52 | 48 | 47 | 45 | 45 | 43 | 41 | 38 | 34 | 34 | 33 | 32 | 31 | 30 | 28 | 28 |
| Fauna (general) | 70 | 63 | 56 | 35 | 43 | 43 | 30 | 33 | 23 | 28 | 18 | 31 | 20 | 23 | 23 | 26 | 12 | 12 | 16 | 28 | 13 | 6 | 9 |
| Regulating Services (general) | 72 | 57 | 50 | 37 | 30 | 33 | 28 | 23 | 16 | 21 | 16 | 19 | 19 | 14 | 21 | 20 | 5 | 15 | 5 | 9 | 11 | 6 | 9 |
| Open Land for Development | 65 | 54 | 45 | 28 | 22 | 27 | 30 | 15 | 21 | 36 | 22 | 11 | 13 | 9 | 20 | 35 | 7 | 4 | 13 | 8 | 9 | 9 | 9 |
| Water Movement/Navigability | 57 | 45 | 48 | 13 | 45 | 34 | 22 | 14 | 21 | 24 | 42 | 18 | 11 | 46 | 21 | 16 | 26 | 6 | 19 | 21 | 11 | 10 | 10 |
| Risk of Flooding | 57 | 45 | 39 | 33 | 24 | 22 | 18 | 13 | 11 | 13 | 17 | 14 | 9 | 11 | 17 | 8 | 5 | 10 | 4 | 6 | 13 | 11 | 11 |
| Water Quality Regulation (Nutrients & Retention) | 71 | 45 | 44 | 24 | 28 | 33 | 23 | 16 | 15 | 22 | 19 | 18 | 12 | 13 | 22 | 17 | 11 | 11 | 11 | 13 | 9 | 7 | 10 |
| Flora (general) | 62 | 41 | 49 | 19 | 27 | 33 | 19 | 18 | 9 | 25 | 16 | 9 | 11 | 16 | 16 | 14 | 6 | 4 | 9 | 9 | 4 | 3 | 7 |
| Water Quantity | 60 | 40 | 41 | 21 | 20 | 27 | 19 | 13 | 12 | 16 | 21 | 16 | 7 | 13 | 13 | 13 | 8 | 6 | 10 | 6 | 5 | 9 | 8 |
| Multiple Ecosystem Components (general) | 39 | 38 | 39 | 18 | 7 | 24 | 23 | 9 | 13 | 11 | 11 | 20 | 8 | 3 | 11 | 14 | 1 | 9 | 2 | 5 | 6 | 15 | 4 |
| Edible Fauna | 42 | 37 | 37 | 16 | 23 | 26 | 9 | 17 | 18 | 11 | 13 | 57 | 8 | 27 | 10 | 9 | 45 | 7 | 8 | 30 | 8 | 3 | 4 |
| Water Quality | 55 | 35 | 45 | 12 | 18 | 28 | 12 | 17 | 13 | 19 | 15 | 13 | 4 | 10 | 11 | 12 | 5 | 7 | 7 | 4 | 9 | 7 | 9 |
| Water (general) | 55 | 31 | 23 | 12 | 21 | 15 | 11 | 11 | 4 | 13 | 6 | 9 | 6 | 1 | 5 | 9 | 6 | 3 | 3 | 7 | 3 | 4 | 5 |
| Charismatic Fauna | 43 | 27 | 25 | 9 | 38 | 27 | 10 | 16 | 9 | 7 | 11 | 13 | 13 | 18 | 9 | 10 | 23 | 10 | 11 | 23 | 6 | 3 | 2 |
| Commercially Important Fauna | 40 | 26 | 21 | 13 | 21 | 18 | 8 | 23 | 21 | 6 | 4 | 41 | 13 | 5 | 8 | 8 | 13 | 7 | 1 | 13 | 7 | 7 | 2 |
| Aesthetic Open Space | 24 | 26 | 4 | 6 | 12 | 6 | 4 | 8 | 3 | 4 | 1 | 1 | 4 | 1 | 4 | 8 | 1 | 1 | 3 | 1 | 2 | 0 | 1 |
| Fauna Community | 40 | 24 | 26 | 13 | 19 | 20 | 4 | 15 | 7 | 5 | 5 | 6 | 6 | 4 | 4 | 6 | 6 | 4 | 2 | 8 | 4 | 5 | 1 |
| Flora Community | 43 | 22 | 23 | 10 | 14 | 18 | 5 | 8 | 6 | 7 | 4 | 5 | 3 | 4 | 4 | 6 | 4 | 5 | 4 | 6 | 1 | 4 | 2 |
| Aesthetic Viewscapes | 33 | 21 | 13 | 2 | 50 | 15 | 4 | 9 | 3 | 1 | 6 | 1 | 9 | 11 | 2 | 7 | 4 | 16 | 9 | 6 | 3 | 2 | 1 |
| Soil & Sediment Regulation | 42 | 21 | 23 | 13 | 13 | 15 | 4 | 9 | 5 | 8 | 13 | 9 | 8 | 7 | 6 | 5 | 4 | 4 | 5 | 2 | 4 | 2 | 5 |
| Charismatic Flora | 36 | 19 | 24 | 9 | 26 | 12 | 6 | 7 | 7 | 2 | 11 | 3 | 9 | 11 | 8 | 11 | 2 | 6 | 7 | 10 | 3 | 1 | 2 |
| Commercially Important Flora | 35 | 16 | 20 | 9 | 10 | 11 | 11 | 6 | 9 | 6 | 6 | 13 | 5 | 2 | 5 | 9 | 2 | 9 | 1 | 2 | 5 | 7 | 1 |
| Rare Fauna | 45 | 16 | 19 | 4 | 7 | 8 | 1 | 6 | 0 | 2 | 2 | 11 | 2 | 1 | 0 | 4 | 2 | 1 | 1 | 4 | 3 | 2 | 2 |
| Risk of Extreme Weather Events | 17 | 13 | 10 | 9 | 6 | 9 | 4 | 5 | 1 | 2 | 6 | 8 | 1 | 3 | 9 | 3 | 1 | 1 | 2 | 3 | 5 | 2 | 1 |
| Soil Quality | 21 | 12 | 9 | 1 | 6 | 5 | 1 | 4 | 2 | 4 | 6 | 2 | 0 | 1 | 1 | 9 | 0 | 1 | 1 | 1 | 1 | 2 | 1 |
| Soil Quantity | 23 | 11 | 7 | 3 | 6 | 3 | 2 | 3 | 2 | 4 | 6 | 4 | 0 | 1 | 4 | 6 | 1 | 1 | 1 | 1 | 1 | 1 | 1 |
| Spiritually/Culturally Important Fauna | 23 | 10 | 9 | 16 | 8 | 7 | 2 | 4 | 2 | 0 | 7 | 5 | 7 | 1 | 4 | 1 | 4 | 4 | 1 | 5 | 1 | 2 | 1 |
| Pest/Invasive Flora | 23 | 9 | 15 | 4 | 7 | 5 | 3 | 2 | 1 | 6 | 2 | 1 | 1 | 3 | 3 | 4 | 1 | 1 | 1 | 1 | 1 | 1 | 1 |
| Pest/Invasive Fauna | 18 | 8 | 13 | 2 | 3 | 5 | 1 | 1 | 1 | 4 | 1 | 6 | 1 | 1 | 4 | 2 | 1 | 0 | 1 | 2 | 6 | 1 | 0 |
| Mineral/Chemical Quantity | 22 | 7 | 9 | 1 | 1 | 2 | 0 | 1 | 0 | 4 | 2 | 1 | 1 | 0 | 1 | 1 | 1 | 2 | 1 | 0 | 1 | 1 | 1 |
| Climate & Carbon Regulation | 23 | 6 | 16 | 4 | 4 | 4 | 1 | 4 | 4 | 2 | 2 | 1 | 1 | 1 | 6 | 1 | 1 | 3 | 1 | 1 | 2 | 0 | 0 |
| Soil (general) | 20 | 6 | 4 | 3 | 3 | 4 | 1 | 2 | 1 | 1 | 1 | 0 | 2 | 1 | 1 | 4 | 0 | 0 | 1 | 3 | 0 | 0 | 1 |
| Air Quality & Atmospheric Regulation | 13 | 6 | 6 | 4 | 6 | 3 | 3 | 1 | 3 | 2 | 4 | 1 | 1 | 1 | 1 | 1 | 1 | 2 | 1 | 1 | 1 | 1 | 1 |
| Culturally Important Flora | 12 | 6 | 4 | 11 | 6 | 3 | 0 | 1 | 3 | 1 | 1 | 1 | 6 | 1 | 1 | 2 | 0 | 5 | 0 | 1 | 0 | 0 | 0 |
| Natural Materials (Sand/Rock) (general) | 5 | 5 | 1 | 0 | 2 | 1 | 1 | 0 | 1 | 1 | 1 | 0 | 1 | 0 | 0 | 1 | 0 | 1 | 1 | 1 | 0 | 1 | 1 |
| Ornamental Natural Materials (Shells/Bone) | 23 | 5 | 8 | 7 | 10 | 4 | 1 | 4 | 3 | 1 | 1 | 1 | 5 | 2 | 1 | 1 | 1 | 1 | 2 | 1 | 1 | 0 | 2 |

| **Percentiles** | **Color** |
| --- | --- |
| 0% |  |
| 0.01-25% |  |
| 25.1-50% |  |
| 50.1-75% |  |
| 75.1-95% |  |
| 95.1-100% |  |

Table F. Comparison of top 10 EEP-by-beneficiary subclass combinations across regions. The numbers in the cells represent the number of documents a particular combination was mentioned in. The colors in the cells represent the percentile thresholds for the top 10 combinations within regions.

| **Beneficiary Subclass/EEP Subclass** | | | Fauna (general) | Flora (general) | Multiple Ecosystem Components (general) | Naturalness | Open Land for Development | Regulating Services (general) | Risk of Flooding | Water Movement/Navigability | Water Quality Regulation (Nutrients & Retention) | Water Quantity |
| --- | --- | --- | --- | --- | --- | --- | --- | --- | --- | --- | --- | --- |
| **Mid-Atlantic** | | | | | | | | | | | | |
| Agriculture (general) | | | 18 | 16 | 4 | 30 | 19 | 16 | 9 | 11 | 13 | 7 |
| Commercial/Industrial Property Owners | | | 9 | 5 | 6 | 28 | 11 | 13 | 3 | 14 | 8 | 5 |
| Educators/Students | | | 22 | 18 | 14 | 32 | 15 | 16 | 14 | 17 | 21 | 15 |
| Experiencers/Viewers | | | 22 | 17 | 3 | 36 | 13 | 23 | 17 | 20 | 15 | 11 |
| Government, Municipal, Residential (general) | | | 34 | 23 | 24 | 46 | 27 | 33 | 24 | 22 | 27 | 22 |
| People Who Care (Existence) | | | 34 | 35 | 23 | 45 | 36 | 39 | 33 | 28 | 39 | 32 |
| People Who Care (Option/Bequest) | | | 20 | 11 | 10 | 38 | 17 | 25 | 19 | 6 | 15 | 9 |
| Recreation (general) | | | 17 | 10 | 2 | 29 | 5 | 12 | 8 | 8 | 11 | 8 |
| Researchers | | | 30 | 26 | 20 | 38 | 28 | 24 | 17 | 23 | 26 | 22 |
| Residential Property Owners | | | 13 | 7 | 12 | 32 | 16 | 15 | 9 | 7 | 13 | 9 |
| **Gulf of Mexico** | | | | | | | | | | | | |
| Agriculture (general) | | | 8 | 5 | 4 | 13 | 9 | 5 | 3 | 5 | 7 | 6 |
| Commercial/Industrial Property Owners | | | 11 | 1 | 7 | 17 | 6 | 4 | 5 | 3 | 7 | 9 |
| Educators/Students | | | 18 | 16 | 10 | 31 | 12 | 13 | 10 | 16 | 15 | 14 |
| Experiencers/Viewers | | | 23 | 9 | 4 | 31 | 8 | 8 | 8 | 19 | 11 | 9 |
| Government, Municipal, Residential (general) | | | 30 | 16 | 15 | 36 | 23 | 21 | 21 | 21 | 22 | 19 |
| People Who Care (Existence) | | | 29 | 24 | 18 | 39 | 28 | 32 | 28 | 25 | 32 | 29 |
| People Who Care (Option/Bequest) | | | 15 | 8 | 12 | 33 | 11 | 17 | 21 | 7 | 11 | 13 |
| Recreation (general) | | | 20 | 7 | 5 | 25 | 9 | 13 | 8 | 7 | 7 | 8 |
| Researchers | | | 23 | 20 | 20 | 34 | 21 | 24 | 23 | 23 | 18 | 21 |
| Residential Property Owners | | | 18 | 8 | 13 | 27 | 14 | 14 | 9 | 11 | 11 | 11 |
| **Pacific Northwest** | | | | | | | | | | | | |
| Agriculture (general) | | | 13 | 14 | 7 | 23 | 23 | 9 | 7 | 18 | 11 | 10 |
| Commercial/Industrial Property Owners | | | 12 | 7 | 6 | 23 | 12 | 6 | 8 | 12 | 6 | 3 |
| Educators/Students | | | 20 | 12 | 10 | 28 | 11 | 18 | 7 | 15 | 11 | 9 |
| Experiencers/Viewers | | | 16 | 12 | 3 | 29 | 10 | 11 | 9 | 24 | 14 | 8 |
| Government, Municipal, Residential (general) | | | 25 | 19 | 15 | 34 | 26 | 26 | 18 | 21 | 14 | 16 |
| People Who Care (Existence) | | | 35 | 29 | 14 | 37 | 27 | 30 | 20 | 27 | 29 | 24 |
| People Who Care (Option/Bequest) | | | 15 | 8 | 3 | 26 | 11 | 10 | 6 | 6 | 8 | 7 |
| Recreation (general) | | | 9 | 9 | 6 | 19 | 7 | 7 | 3 | 5 | 4 | 2 |
| Researchers | | | 26 | 23 | 15 | 32 | 14 | 23 | 15 | 21 | 18 | 15 |
| Residential Property Owners | | | 12 | 12 | 7 | 22 | 13 | 11 | 7 | 13 | 9 | 7 |
| **Percentiles** | **Color** |  |  |  |  |  |  |  |  |  |  |  |
| 0% |  |  |  |  |  |  |  |  |  |  |  |  |
| 0.01-25% |  |  |  |  |  |  |  |  |  |  |  |  |
| 25.1-50% |  |  |  |  |  |  |  |  |  |  |  |  |
| 50.1-75% |  |  |  |  |  |  |  |  |  |  |  |  |
| 75.1-95% |  |  |  |  |  |  |  |  |  |  |  |  |
| 95.1-100% |  |  |  |  |  |  |  |  |  |  |  |  |

Table G. Comparison of top 10 EEP-by-beneficiary subclass combinations across organizations. The numbers in the boxes represent the number of documents a particular combination was mentioned. The colors in the cells represent the percentile thresholds for the top 10 combinations within organizations.

| **Beneficiary Subclass/EEP Subclass** | | | Fauna (general) | Flora (general) | Multiple Ecosystem Components (general) | Naturalness | Open Land for Development | Regulating Services (general) | Risk of Flooding | Water Movement/Navigability | Water Quality Regulation (Nutrients & Retention) | Water Quantity |
| --- | --- | --- | --- | --- | --- | --- | --- | --- | --- | --- | --- | --- |
| **Federal Agencies** | | | | | | | | | | | | |
| Agriculture (general) | | | 8 | 2 | 3 | 8 | 8 | 3 | 2 | 2 | 5 | 4 |
| Commercial/Industrial Property Owners | | | 5 | 1 | 2 | 9 | 2 | 5 | 5 | 1 | 5 | 5 |
| Educators/Students | | | 3 | 5 | 3 | 8 | 3 | 6 | 4 | 5 | 6 | 3 |
| Experiencers/Viewers | | | 7 | 3 | 3 | 10 | 3 | 2 | 4 | 7 | 8 | 6 |
| Government, Municipal, Residential (general) | | | 9 | 7 | 5 | 11 | 9 | 7 | 7 | 7 | 5 | 7 |
| People Who Care (Existence) | | | 10 | 7 | 6 | 13 | 8 | 10 | 7 | 9 | 10 | 7 |
| People Who Care (Option/Bequest) | | | 7 | 4 | 4 | 9 | 6 | 6 | 6 | 2 | 4 | 3 |
| Recreation (general) | | | 6 | 5 | 4 | 8 | 4 | 4 | 5 | 4 | 5 | 5 |
| Researchers | | | 8 | 7 | 5 | 13 | 6 | 8 | 6 | 8 | 8 | 7 |
| Residential Property Owners | | | 4 | 5 | 3 | 9 | 4 | 5 | 5 | 4 | 5 | 4 |
| **State and Local Agencies** | | | | | | | | | | | | |
| Agriculture (general) | | | 4 | 7 | 0 | 20 | 14 | 10 | 6 | 4 | 6 | 5 |
| Commercial/Industrial Property Owners | | | 3 | 2 | 3 | 20 | 7 | 8 | 4 | 10 | 7 | 3 |
| Educators/Students | | | 11 | 11 | 12 | 25 | 8 | 15 | 9 | 7 | 13 | 9 |
| Experiencers/Viewers | | | 8 | 15 | 2 | 26 | 6 | 12 | 14 | 14 | 11 | 5 |
| Government, Municipal, Residential (general) | | | 21 | 15 | 18 | 37 | 20 | 25 | 24 | 18 | 25 | 20 |
| People Who Care (Existence) | | | 25 | 22 | 10 | 37 | 19 | 29 | 25 | 17 | 26 | 19 |
| People Who Care (Option/Bequest) | | | 6 | 6 | 8 | 28 | 7 | 16 | 15 | 5 | 10 | 3 |
| Recreation (general) | | | 3 | 4 | 4 | 19 | 3 | 5 | 8 | 3 | 5 | 5 |
| Researchers | | | 13 | 13 | 16 | 27 | 13 | 18 | 13 | 13 | 15 | 11 |
| Residential Property Owners | | | 3 | 4 | 10 | 28 | 12 | 10 | 9 | 7 | 11 | 5 |
| **Land Stewards** | | | | | | | | | | | | |
| Agriculture (general) | | | 23 | 24 | 10 | 30 | 23 | 14 | 8 | 23 | 15 | 10 |
| Commercial/Industrial Property Owners | | | 21 | 7 | 13 | 32 | 14 | 6 | 5 | 15 | 6 | 6 |
| Educators/Students | | | 41 | 25 | 16 | 48 | 23 | 24 | 15 | 32 | 21 | 20 |
| Experiencers/Viewers | | | 41 | 18 | 5 | 51 | 20 | 24 | 15 | 34 | 18 | 16 |
| Government, Municipal, Residential (general) | | | 49 | 31 | 27 | 54 | 38 | 38 | 25 | 32 | 25 | 23 |
| People Who Care (Existence) | | | 51 | 48 | 38 | 55 | 50 | 51 | 40 | 45 | 50 | 48 |
| People Who Care (Option/Bequest) | | | 31 | 16 | 11 | 46 | 21 | 24 | 19 | 8 | 16 | 17 |
| Recreation (general) | | | 35 | 15 | 4 | 43 | 13 | 21 | 4 | 13 | 10 | 7 |
| Researchers | | | 47 | 41 | 27 | 49 | 37 | 38 | 30 | 39 | 31 | 30 |
| Residential Property Owners | | | 31 | 15 | 15 | 37 | 22 | 20 | 10 | 16 | 14 | 14 |
| **Wetland Conservation Organizations** | | | | | | | | | | | | |
| Agriculture (general) | | | 4 | 2 | 2 | 8 | 6 | 3 | 3 | 5 | 5 | 4 |
| Commercial/Industrial Property Owners | | | 3 | 3 | 1 | 7 | 6 | 4 | 2 | 3 | 3 | 3 |
| Educators/Students | | | 5 | 5 | 3 | 10 | 4 | 2 | 3 | 4 | 7 | 6 |
| Experiencers/Viewers | | | 5 | 2 | 0 | 9 | 2 | 4 | 1 | 8 | 3 | 1 |
| Government, Municipal, Residential (general) | | | 10 | 5 | 4 | 14 | 9 | 10 | 7 | 7 | 8 | 7 |
| People Who Care (Existence) | | | 12 | 11 | 1 | 16 | 14 | 11 | 9 | 9 | 14 | 11 |
| People Who Care (Option/Bequest) | | | 6 | 1 | 2 | 14 | 5 | 6 | 6 | 4 | 4 | 6 |
| Recreation (general) | | | 2 | 2 | 1 | 3 | 1 | 2 | 2 | 0 | 2 | 1 |
| Researchers | | | 11 | 8 | 7 | 15 | 7 | 7 | 6 | 7 | 8 | 10 |
| Residential Property Owners | | | 5 | 3 | 4 | 7 | 5 | 5 | 1 | 4 | 3 | 4 |
| **Percentiles** | **Color** |  |  |  |  |  |  |  |  |  |  |  |
| 0% |  |  |  |  |  |  |  |  |  |  |  |  |
| 0.01-25% |  |  |  |  |  |  |  |  |  |  |  |  |
| 25.1-50% |  |  |  |  |  |  |  |  |  |  |  |  |
| 50.1-75% |  |  |  |  |  |  |  |  |  |  |  |  |
| 75.1-95% |  |  |  |  |  |  |  |  |  |  |  |  |
| 95.1-100% |  |  |  |  |  |  |  |  |  |  |  |  |

Table H. Comparison of top 10 EEP-by-beneficiary subclass combinations across tidal wetland types. The numbers in the boxes represent the number of documents within which a particular combination was mentioned. The colors in the cells represent the percentile thresholds for the top 10 combinations within tidal wetland types.

| **Beneficiary Subclass/EEP Subclass** | | | Fauna (general) | Flora (general) | Multiple Ecosystem Components (general) | Naturalness | Open Land for Development | Regulating Services (general) | Risk of Flooding | Water Movement/Navigability | Water Quality Regulation (Nutrients & Retention) | Water Quantity |
| --- | --- | --- | --- | --- | --- | --- | --- | --- | --- | --- | --- | --- |
| **Tidal Wetlands (general)** | | | | | | | | | | | | |
| Agriculture (general) | | | 32 | 24 | 10 | 62 | 48 | 27 | 11 | 30 | 29 | 20 |
| Commercial/Industrial Property Owners | | | 23 | 8 | 18 | 62 | 24 | 20 | 15 | 22 | 17 | 16 |
| Educators/Students | | | 54 | 39 | 28 | 87 | 31 | 45 | 26 | 39 | 42 | 33 |
| Experiencers/Viewers | | | 53 | 25 | 9 | 89 | 26 | 37 | 24 | 52 | 35 | 21 |
| Government, Municipal, Residential (general) | | | 80 | 49 | 49 | 110 | 74 | 79 | 60 | 58 | 56 | 49 |
| People Who Care (Existence) | | | 96 | 80 | 53 | 116 | 88 | 100 | 73 | 76 | 95 | 82 |
| People Who Care (Option/Bequest) | | | 40 | 15 | 21 | 84 | 33 | 42 | 38 | 15 | 28 | 25 |
| Recreation (general) | | | 37 | 19 | 13 | 67 | 17 | 28 | 18 | 17 | 16 | 15 |
| Researchers | | | 72 | 59 | 47 | 97 | 60 | 66 | 48 | 55 | 58 | 54 |
| Residential Property Owners | | | 37 | 21 | 29 | 75 | 41 | 37 | 23 | 24 | 30 | 25 |
| **Emergent Wetlands** | | | | | | | | | | | | |
| Agriculture (general) | | | 10 | 15 | 2 | 10 | 11 | 4 | 7 | 11 | 1 | 3 |
| Commercial/Industrial Property Owners | | | 10 | 6 | 2 | 15 | 5 | 3 | 2 | 5 | 3 | 1 |
| Educators/Students | | | 22 | 16 | 8 | 38 | 11 | 9 | 12 | 11 | 10 | 9 |
| Experiencers/Viewers | | | 21 | 18 | 0 | 36 | 8 | 14 | 13 | 12 | 6 | 8 |
| Government, Municipal, Residential (general) | | | 36 | 23 | 7 | 52 | 17 | 15 | 19 | 16 | 18 | 16 |
| People Who Care (Existence) | | | 56 | 46 | 14 | 70 | 35 | 43 | 29 | 35 | 35 | 25 |
| People Who Care (Option/Bequest) | | | 18 | 12 | 3 | 40 | 10 | 14 | 12 | 3 | 7 | 3 |
| Recreation (general) | | | 9 | 8 | 0 | 22 | 4 | 3 | 1 | 4 | 8 | 5 |
| Researchers | | | 46 | 41 | 11 | 54 | 14 | 24 | 20 | 26 | 18 | 18 |
| Residential Property Owners | | | 10 | 13 | 2 | 15 | 9 | 8 | 3 | 9 | 4 | 4 |
| **Forested Wetlands** | | | | | | | | | | | | |
| Agriculture (general) | | | 3 | 1 | 4 | 6 | 1 | 2 | 4 | 6 | 1 | 4 |
| Commercial/Industrial Property Owners | | | 4 | 1 | 2 | 7 | 2 | 2 | 2 | 3 | 1 | 1 |
| Educators/Students | | | 7 | 5 | 4 | 21 | 8 | 3 | 3 | 9 | 8 | 2 |
| Experiencers/Viewers | | | 13 | 4 | 1 | 25 | 5 | 3 | 3 | 14 | 4 | 5 |
| Government, Municipal, Residential (general) | | | 25 | 8 | 6 | 27 | 13 | 5 | 12 | 15 | 8 | 5 |
| People Who Care (Existence) | | | 37 | 24 | 10 | 45 | 22 | 17 | 14 | 20 | 21 | 14 |
| People Who Care (Option/Bequest) | | | 3 | 4 | 2 | 15 | 5 | 2 | 2 | 5 | 5 | 5 |
| Recreation (general) | | | 11 | 4 | 1 | 11 | 3 | 1 | 2 | 3 | 4 | 3 |
| Researchers | | | 21 | 9 | 9 | 28 | 7 | 5 | 5 | 16 | 13 | 6 |
| Residential Property Owners | | | 9 | 6 | 3 | 13 | 5 | 1 | 4 | 6 | 4 | 2 |
| **Scrub-shrub Wetlands** | | | | | | | | | | | | |
| Agriculture (general) | | | 2 | 0 | 0 | 2 | 2 | 0 | 0 | 0 | 0 | 0 |
| Commercial/Industrial Property Owners | | | 0 | 0 | 0 | 1 | 0 | 0 | 0 | 0 | 0 | 0 |
| Educators/Students | | | 1 | 1 | 0 | 3 | 1 | 1 | 0 | 2 | 0 | 0 |
| Experiencers/Viewers | | | 3 | 4 | 0 | 5 | 0 | 4 | 4 | 0 | 0 | 0 |
| Government, Municipal, Residential (general) | | | 2 | 4 | 0 | 5 | 1 | 1 | 1 | 2 | 1 | 0 |
| People Who Care (Existence) | | | 13 | 6 | 0 | 16 | 4 | 6 | 3 | 3 | 3 | 1 |
| People Who Care (Option/Bequest) | | | 1 | 0 | 0 | 0 | 1 | 1 | 0 | 1 | 0 | 0 |
| Recreation (general) | | | 1 | 1 | 0 | 0 | 0 | 0 | 0 | 0 | 0 | 0 |
| Researchers | | | 6 | 2 | 0 | 4 | 2 | 1 | 0 | 5 | 1 | 0 |
| Residential Property Owners | | | 1 | 1 | 0 | 2 | 0 | 0 | 0 | 1 | 0 | 0 |
| **Percentiles** | **Color** |  |  |  |  |  |  |  |  |  |  |  |
| 0% |  |  |  |  |  |  |  |  |  |  |  |  |
| 0.01-25% |  |  |  |  |  |  |  |  |  |  |  |  |
| 25.1-50% |  |  |  |  |  |  |  |  |  |  |  |  |
| 50.1-75% |  |  |  |  |  |  |  |  |  |  |  |  |
| 75.1-95% |  |  |  |  |  |  |  |  |  |  |  |  |
| 95.1-100% |  |  |  |  |  |  |  |  |  |  |  |  |

Table I. Comparison of top 5 EEP-by-beneficiary subclass combinations across regions, organizations, and tidal wetland types. The numbers in the cells represent the number of documents a particular combination was mentioned in. The colors in the cells represent the percentile thresholds for the top 5 combinations within regions, organizations, and tidal wetland types independently.

| **Beneficiary Subclass/EEP Subclass** | | | | | Fauna (general) | Naturalness | | Open Land for Development | Regulating Services (general) | Water Movement/Navigability | Fauna (general) | Naturalness | Open Land for Development | Regulating Services (general) | Water Movement/Navigability | Fauna (general) | Naturalness | Open Land for Development | Regulating Services (general) | Water Movement/Navigability |
| --- | --- | --- | --- | --- | --- | --- | --- | --- | --- | --- | --- | --- | --- | --- | --- | --- | --- | --- | --- | --- |
|  | | | | | **Mid-Atlantic** | | | | | | **Federal Agencies** | | | | | **Tidal Wetlands (general)** | | | | |
| Experiencers/Viewers | | | | | 22 | 36 | | 13 | 23 | 20 | 7 | 10 | 3 | 2 | 7 | 53 | 89 | 26 | 37 | 52 |
| Government, Municipal, Residential (general) | | | | | 34 | 46 | | 27 | 33 | 22 | 9 | 11 | 9 | 7 | 7 | 80 | 110 | 74 | 79 | 58 |
| People Who Care (Existence) | | | | | 34 | 45 | | 36 | 39 | 28 | 10 | 13 | 8 | 10 | 9 | 96 | 116 | 88 | 100 | 76 |
| People Who Care (Option/Bequest) | | | | | 20 | 38 | | 17 | 25 | 6 | 7 | 9 | 6 | 6 | 2 | 40 | 84 | 33 | 42 | 15 |
| Researchers | | | | | 30 | 38 | | 28 | 24 | 23 | 8 | 13 | 6 | 8 | 8 | 72 | 97 | 60 | 66 | 55 |
|  | | | | | **Gulf of Mexico** | | | | | | **State and Local Agencies** | | | | | **Emergent Wetlands** | | | | |
| Experiencers/Viewers | | | | | 23 | 31 | | 8 | 8 | 19 | 8 | 26 | 6 | 12 | 14 | 21 | 36 | 8 | 14 | 12 |
| Government, Municipal, Residential (general) | | | | | 30 | 36 | | 23 | 21 | 21 | 21 | 37 | 20 | 25 | 18 | 36 | 52 | 17 | 15 | 16 |
| People Who Care (Existence) | | | | | 29 | 39 | | 28 | 32 | 25 | 25 | 37 | 19 | 29 | 17 | 56 | 70 | 35 | 43 | 35 |
| People Who Care (Option/Bequest) | | | | | 15 | 33 | | 11 | 17 | 7 | 6 | 28 | 7 | 16 | 5 | 18 | 40 | 10 | 14 | 3 |
| Researchers | | | | | 23 | 34 | | 21 | 24 | 23 | 13 | 27 | 13 | 18 | 13 | 46 | 54 | 14 | 24 | 26 |
|  | | | | | **Pacific Northwest** | | | | | | **Land Stewards** | | | | | **Forested Wetlands** | | | | |
| Experiencers/Viewers | | | | | 16 | 29 | | 10 | 11 | 24 | 41 | 51 | 20 | 24 | 34 | 13 | 25 | 5 | 3 | 14 |
| Government, Municipal, Residential (general) | | | | | 25 | 34 | | 26 | 26 | 21 | 49 | 54 | 38 | 38 | 32 | 25 | 27 | 13 | 5 | 15 |
| People Who Care (Existence) | | | | | 35 | 37 | | 27 | 30 | 27 | 51 | 55 | 50 | 51 | 45 | 37 | 45 | 22 | 17 | 20 |
| People Who Care (Option/Bequest) | | | | | 15 | 26 | | 11 | 10 | 6 | 31 | 46 | 21 | 24 | 8 | 3 | 15 | 5 | 2 | 5 |
| Researchers | | | | | 26 | 32 | | 14 | 23 | 21 | 47 | 49 | 37 | 38 | 39 | 21 | 28 | 7 | 5 | 16 |
|  | | | | |  | | | | | | **Wetland Conservation Orgs** | | | | | **Scrub-Shrub Wetlands** | | | | |
| Experiencers/Viewers | | | | |  |  | |  |  |  | 5 | 9 | 2 | 4 | 8 | 3 | 5 | 0 | 4 | 0 |
| Government, Municipal, Residential (general) | | | | |  |  | |  |  |  | 10 | 14 | 9 | 10 | 7 | 2 | 5 | 1 | 1 | 2 |
| People Who Care (Existence) | | | | |  |  | |  |  |  | 12 | 16 | 14 | 11 | 9 | 13 | 16 | 4 | 6 | 3 |
| People Who Care (Option/Bequest) | | | | |  |  | |  |  |  | 6 | 14 | 5 | 6 | 4 | 1 | 0 | 1 | 1 | 1 |
| Researchers | | | | |  |  | |  |  |  | 11 | 15 | 7 | 7 | 7 | 6 | 4 | 2 | 1 | 5 |
| **Regions** | | **Organizations** | | **Tidal Wetland Types** | | | | |  |  |  |  |  |  |  |  |  |  |  |  |
| **Percentiles** | **Color** | **Percentiles** | **Color** | **Percentiles** | | | **Color** | |  |  |  |  |  |  |  |  |  |  |  |  |
| 0% |  | 0% |  | 0% | | |  | |  |  |  |  |  |  |  |  |  |  |  |  |
| 0.1-25% |  | 0.1-25% |  | 0.1-25% | | |  | |  |  |  |  |  |  |  |  |  |  |  |  |
| 25.1-50% |  | 25.1-50% |  | 25.1-50% | | |  | |  |  |  |  |  |  |  |  |  |  |  |  |
| 50.1-75% |  | 50.1-75% |  | 50.1-75% | | |  | |  |  |  |  |  |  |  |  |  |  |  |  |
| 75.1-95% |  | 75.1-95% |  | 75.1-95% | | |  | |  |  |  |  |  |  |  |  |  |  |  |  |
| 95.1-100% |  | 95.1-100% |  | 95.1-100% | | |  | |  |  |  |  |  |  |  |  |  |  |  |  |
